# The contribution of avoidable mortality to life expectancy differences and lifespan disparities in the European Union: a population-based study

**DOI:** 10.1016/j.lanepe.2024.101042

**Published:** 2024-08-30

**Authors:** Rok Hrzic, Tobias Vogt

**Affiliations:** a Department of International Health, Care and Public Health Research Institute (CAPHRI), Maastricht University, 6200 MD, Maastricht, the Netherlands; b Population Research Centre, Faculty of Spatial Sciences, University of Groningen, 9700 AV, Groningen, the Netherlands; c Prasanna School of Public Health, Manipal Academy of Higher Education, Karnataka, 576104, India

**Keywords:** Avoidable mortality, European union, Mortality decomposition, Health system performance assessment

## Abstract

**Background:**

Twenty years after the 2004 European Union (EU) enlargement, life expectancy differences between established (EMS) and new member states (NMS) remain large. Contributing to this gap are deaths that can be avoided through preventive services or adequate medical treatment. We estimate the impact of reducing avoidable mortality on life expectancy and lifespan disparities in the enlarged EU.

**Methods:**

Using World Health Organization mortality database data, we analysed the potential of reducing avoidable mortality, as defined by Eurostat and the Organisation for Economic Cooperation and Development, to close the mortality gap between NMS and EMS. We decomposed the changes in life expectancy and lifespan disparity by age and cause using linear integral decomposition.

**Findings:**

Averting all avoidable deaths across the EU from 2005 to 2019 would decrease the average life expectancy gap from 5.8 to 2.4 years in men and 3.3–2 years in women and eliminate the lifespan disparity gap. Had NMS achieved the average EMS avoidable mortality rates during the same period, the average life expectancy gap would have been reduced to 1.8 years in men and 1.6 years in women, and the lifespan disparities gap would have been reversed. Avoidable circulatory and injury-related deaths in middle and older age drove the observed mortality changes.

**Interpretation:**

Our results suggest that the gap in life expectancy and lifespan disparity across the EU could be reduced by strengthening health systems and investing in averting circulatory and injury-related deaths in middle and older age in NMS.

**Funding:**

None.

**Research in context**

**Evidence before this study**

A literature search in June 2024 in PubMed and the Web of Science Core Collection using the search terms “avoidable mortality”, “preventable mortality”, “amenable mortality”, “treatable mortality”, and “European Union”, showed there are 8 existing studies focused on avoidable mortality (including preventable and treatable mortality) in the context of the enlarged European Union (i.e., after 2004). The studies documented levels and trends of avoidable mortality across the EU and highlighted differences in rates between Western and Eastern Europe. However, they did not focus on the potential impact of averting avoidable deaths on the gap in life expectancy and lifespan disparity between new and established member states.

**Added value of this study**

Our study explicitly examined the impact of averting avoidable deaths on life expectancy and lifespan disparity gaps in the enlarged EU up to the COVID-19 pandemic. We constructed two scenarios to analyse the potential of new member states to close the mortality gap with established member states by (1) eliminating or (2) reducing mortality due to avoidable causes of death. We also examined the relative importance of different groups of avoidable causes of death and age categories, which identified aspects of the new member state health systems with the greatest potential for positive impact on the EU mortality gap.

**Implications of all the available evidence**

Our results show that avoidable causes of death comprise a sizeable portion of the EU's mortality gaps. Averting all avoidable deaths between 2005 and 2019 would have reduced the average life expectancy gap in men and women by 3.4 and 1.3 years, respectively, while the lifespan disparity gap would be closed. In a second, more realistic scenario, we show that the EU's life expectancy gap could have been reduced by 4 years in men and 1.7 years in women, and the direction of the lifespan disparity gap reversed if new EU member states had matched the average performance of established member states in averting avoidable deaths. The main driving force for achieving these gains is the reduction of avoidable circulatory and injury-related mortality in middle and older ages. Strengthening new member state health systems to avert these deaths could substantially reduce EU's persistent geographic mortality gaps.

## Introduction

Life expectancy differences between established and new European Union member states remain large. Despite overall mortality improvements in the last decades, new member states did not catch up to the established member states’ level.^1^^,^^2^ Male and female life expectancies in all Eastern European countries, except Slovenia, are below the EU average. Women in Bulgaria had a life expectancy at birth of 78.7 in 2019 (before the COVID-19 pandemic), which is 7.4 years less than women in Spain in the same year. Similarly, men in Lithuania lagged by 9.6 years behind men in Italy.^3^ Past research has thoroughly analysed trends in life expectancy and the underlying age and cause-specific contribution to these persisting inequalities.^4^^,^^5^ Simultaneously, an ever-increasing number of studies seeks to identify the determinants for the comparatively lower life expectancy levels in the new member states. They find that mostly different smoking,^6^ nutrition,^7^ or alcohol consumption patterns,^8^ as well as health policies and healthcare quality,^9^ account for the lower life expectancies and the lagging behind of new member states. These patterns might also reflect the larger socioeconomic inequalities within European countries that account for differences in life expectancy levels across the European Union.^10^ Despite the decline in mortality among lowly educated Eastern Europeans in recent years,^11^ the absolute differences in life expectancy between socioeconomic groups remain large.^12^

Deaths caused by adverse health behaviours or by poor healthcare quality are considered avoidable either through prevention (preventable mortality) or through adequate medical treatment (treatable or amenable mortality). Hence, all deaths that could potentially be avoided are premature or untimely.^13^ The concepts of avoidable, preventable and treatable mortality have been applied in different studies to estimate the number of years of life that are lost prematurely and to determine the extent to which European health and mortality disparities are based on differences in health policies or health behaviours.^14^^,^^15^

Existing research on avoidable causes of death in the EU has mainly focused on trends in life expectancies or age-specific mortality, including differences by socioeconomic status.^16^ Weber and Clerc compared the annual treatable mortality rates in 28 EU countries for 1994–2013 and found that treatable mortality rates across the EU are converging due to the rapid reductions in cardiovascular and cerebrovascular mortality in most new member states.^17^ Karanikolos et al.^14^ and Costa and Santana^18^ found evidence for the association between time trends in avoidable and treatable mortality rates and the 2008 economic crisis at national and regional levels, respectively. There are studies on trends in lifespan disparities^19^ in selected European countries, including Eastern and Central Eastern European countries.^20^^,^^21^ However, no studies to date have focused on the contribution of avoidable mortality to lifespan disparities, particularly in the context of EU enlargement. This particular focus on variations in the average length of life also accounts for potential reductions in mortality inequalities within and across countries since lifespan disparity is inversely associated with socioeconomic position.^22^

Our study adds to this literature by estimating the contribution of avoidable mortality to life expectancy and lifespan disparities in the context of 21st-century EU enlargements up to the COVID-19 pandemic. We construct two scenarios to analyse the potential of new member states to close the gap with established member states by eliminating or reducing avoidable causes of death and examine the relative importance of different groups of avoidable causes of death. Prevention and strengthening health systems are at the core of the EU4Health program, representing two of the four major objectives of this initiative.^23^ This is of central importance in the EU since one of the priorities of European integration is to achieve comparable living standards for all EU citizens, including health.

## Methods

### Concepts and measures

Avoidable mortality is a measure that can serve as a warning signal and help identify areas of poor performance in healthcare systems.^13^ It comprises preventable mortality, which reflects the quality of preventative services, and treatable mortality, which reflects the quality of curative services.^24^ It has been used extensively in comparative health system performance assessment in the European context, including exploring the reasons for the East-West mortality gap.^25^^,^^26^ The core of the concept is a list of diseases and accompanying age ranges, which define deaths due to specific causes and at specific ages that should not occur in a health system that provides access to high-quality preventive and curative services. For example, the case-fatality rate of appendicitis can be reduced through early detection and appropriate treatment, while lung cancer deaths can be largely prevented through prevention measures such as smoking cessation. Expert opinion determines which deaths (due to which causes and at which ages) should be included in the list. The OECD/Eurostat list includes 100 causes of death (47 preventable and 57 treatable, with deaths due to some causes split across both categories) and uses the age cutoff of 74 years. Avoidable mortality has conceptual limitations in a comparative context (see Brand et al.^27^ for a recent review), and we reflect on the most relevant ones for this study in the Limitations section of the Discussion.

Period life expectancy at birth (e_0_) is a summary measure of mortality conditions in a given population at a given time. It transforms observed age-specific death rates into death and survival probabilities, which are then used to calculate the mean age at death for a synthetic cohort corresponding to life expectancy at birth.^28^

Lifespan disparity (or e-dagger, *e*^d^) measures one of the most fundamental inequalities in population health, the disparities in the average length of lives.^19^ It is defined as the average remaining life expectancy at the ages when death occurs and measures life years lost due to death. Lower lifespan disparity indicates a more equal distribution of years of life lived across the population. While averting deaths at any age increases life expectancy, lifespan disparity narrows when the distribution of lifespans is compressed, which normally occurs when lives are saved at earlier rather than later ages.

### Data

For our analysis, we used death and population counts by age group (0, 1–4, … 80–84, 85+) and cause (ICD-10) available from the World Health Organization Mortality Database^29^ to calculate age and cause-specific mortality rates for EU member states for the years 2005–2019 (see Table 1). The WHO mortality database uses deaths registered in national vital registration systems with underlying causes of death coded by the relevant national authority. We excluded Greece due to its comparably late implementation of ICD10 in 2014; for the same reason, Ireland is included from 2007 onwards. The data were otherwise complete. We categorised causes of death as avoidable, treatable, and preventable based on the OECD/Eurostat list of preventable and treatable causes of death.^30^

**Table 1:** Overview of European Union member states and their year of accession.

Country	Year of EU accession
Austria	1995
Belgium	1957
Bulgaria	2007
Croatia	2013
Cyprus	2004
Czech Republic	2004
Denmark	1973
Estonia	2004
Finland	1995
France	1957
Germany	1957
Greece^a^	1974
Hungary	2004
Ireland	1973
Italy	1957
Latvia	2004
Lithuania	2004
Luxembourg	1957
Malta	2004
Netherlands	1957
Poland	2004
Portugal	1986
Romania	2007
Slovakia	2004
Slovenia	2004
Spain	1986
Sweden	1995
United Kingdom	1973 (left in 2020)

Notes: In our analysis, the countries in shaded rows are defined as new member states. The remaining are defined as Established member states.

a Greece was not included in the analysis.

### Statistical analysis

First, we used the all-cause death and population counts in 5-year age groups (0, 1–4, 5–9, … 8–84, 85+) to construct life tables and calculate the period life expectancy at birth (*e*_0_) and the lifespan disparity (*e*^d^) by sex, country, and year. We performed a sensitivity check, comparing our *e*_0_ estimates with those of the Human Mortality Database^3^ and found only small differences between them (Supplementary material Fig. S1). We used the estimated *e*_0_ and *e*^d^ as the baseline in the next steps.

Second, we quantified the extent to which avoidable causes of death contribute to the observed differences in life expectancy and lifespan disparity between new and established member states. We constructed two counterfactual scenarios. In the first scenario, we simulated deleting all avoidable deaths across all included countries. In the second, more realistic scenario, we simulated assigning new member states the average level of avoidable deaths observed in established member states. The second scenario was constructed by reducing the observed age-, sex-, and cause-specific mortality rates in the new member states to match the average rates observed across established member states. We constructed cause-deleted life tables and calculated the counterfactual *e*_0_ and *e*^d^ by sex, country, year, and scenario using standard methods.^28^

Finally, we decomposed the potential gains in life expectancy and changes in lifespan disparity by age and fourteen groups of avoidable causes of death using linear integral decomposition.^31^ Compared to other common decomposition methods, this approach is based on an explicit mathematical model (the line integral model of decomposition), eliminates interaction effects, is more flexible in the number of covariates and their ordering, and is computationally more efficient. The decomposition analysis identified the years of life expectancy gain or lifespan disparity reduction attributable to reducing avoidable mortality in each age and avoidable cause of death group used in this study. As a result, the decomposition highlighted the age groups and causes of death that could be prioritised to reduce the mortality gaps between new and established member states.

All analyses were performed using the R statistical language.^32^ The demographic variables were calculated using modified functions based on the *DemoTools* and *DemoDecomp* packages.^33^^,^^34^ All code is freely available on Github (https://github.com/rhrzic/TLRHE_AvoidMortEU).

### Role of the funding source

None.

## Results

### Changes in life expectancy and lifespan disparity

#### Scenario 1

**Fig. 1:**
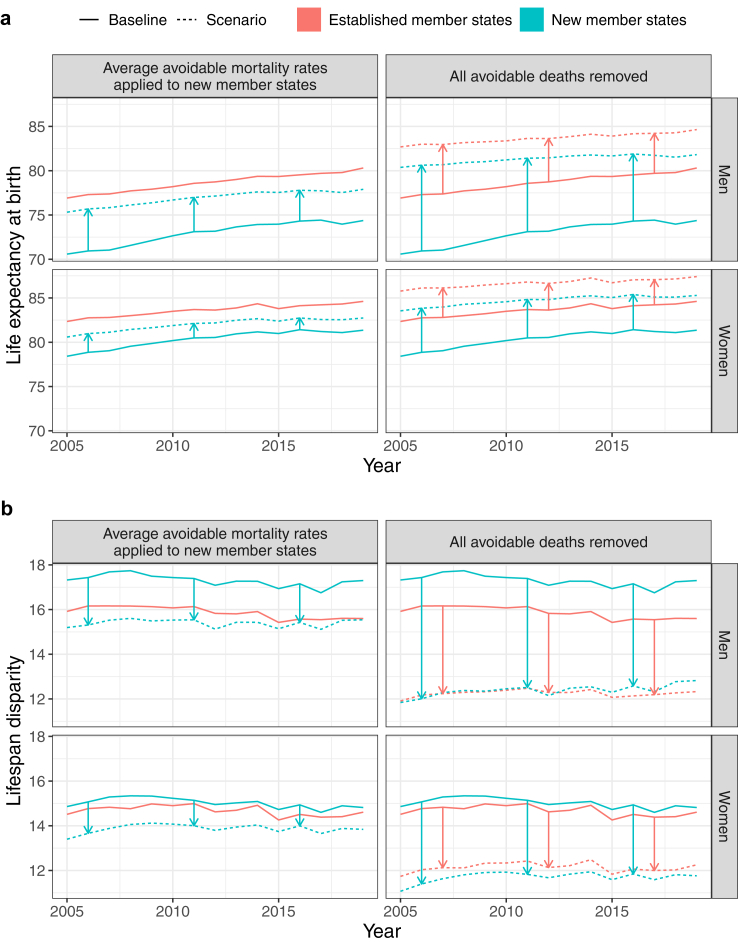
**Trends in life expectancy at birth (a) and lifespan disparity (b) in the established and new member states under different scenarios, 2005–2019, by sex**.

Were all avoidable deaths averted throughout the EU, the gains in life expectancy at birth and the reduction in lifespan dispersion would have been substantially greater in the new member states compared to established member states (see Table 2 and Fig. 1, panel a). Life expectancy in the new member states would be an average of 8.4 years higher in men and 4.4 years in women. The same holds for lifespan disparity, which would be 4.9 years lower in men and 3.3 years in women in the new member states (see Table 2 and Fig. 1, panel b). The Baltic States would benefit most (see Supplementary Tables S1–S4). As a result, the average life expectancy gap between the established and new member states would be 3.4 years lower in men and 1.3 years lower in women, while the gap in lifespan disparity would no longer exist.

**Table 2:** Life expectancy at birth and lifespan disparity in new and established member states under different scenarios, 2005–2019, by sex.

Sex	Scenario	Life expectancy at birth			Lifespan disparity		
		NMS^a^	EMS^b^	Difference	NMS	EMS	Difference
Men	Status quo	72.87	78.65	−5.78	17.30	15.87	1.43
	Average mortality rates for all avoidable causes applied to NMS	76.88	78.65	−1.77	15.39	15.87	−0.48
	Average mortality rates for only preventable causes applied to NMS	75.40	78.65	−3.25	16.14	15.87	0.27
	Average mortality rates for only treatable causes applied to NMS	74.15	78.65	−4.50	16.83	15.87	0.96
	All avoidable deaths removed	81.30	83.66	−2.36	12.38	12.26	0.12
	Only preventable deaths removed	78.17	82.08	−3.91	14.49	13.49	1.00
	Only treatable deaths removed	75.30	80.05	−4.75	16.24	14.97	1.27
Women	Status quo	80.31	83.62	−3.31	15.03	14.68	0.35
	Average mortality rates for all avoidable causes applied to NMS	81.98	83.62	−1.64	13.87	14.68	−0.81
	Average mortality rates for only preventable causes applied to NMS	81.15	83.62	−2.47	14.45	14.68	−0.23
	Average mortality rates for only treatable causes applied to NMS	81.13	83.62	−2.49	14.48	14.68	−0.20
	All avoidable deaths removed	84.68	86.69	−2.01	11.70	12.14	−0.44
	Only preventable deaths removed	82.44	85.23	−2.79	13.50	13.40	0.10
	Only treatable deaths removed	82.38	85.01	−2.63	13.55	13.57	−0.02

a New member states.

b Established member states.

Considering the split of avoidable causes into preventable and treatable (i.e., avoided through preventive services or high-quality clinical care), the improvement in life expectancy would have been derived approximately equally from both in women, while in men greater gains would be derived from avoiding preventable causes of death for both new and established member states.

#### Scenario 2

In the scenario where new member states are assigned the average avoidable mortality rates observed across the established member states, their average life expectancy is 4 years higher in men and 1.7 years higher in women. This is also how much smaller the average life expectancy gap with the established member states would be in this scenario. Considering lifespan disparity, the reduction would be 1.9 years for men and 1.2 years for women. This would give new member states an advantage in lifespan disparity over established member states.

### Decomposition

#### Scenario 1

**Fig. 2:**
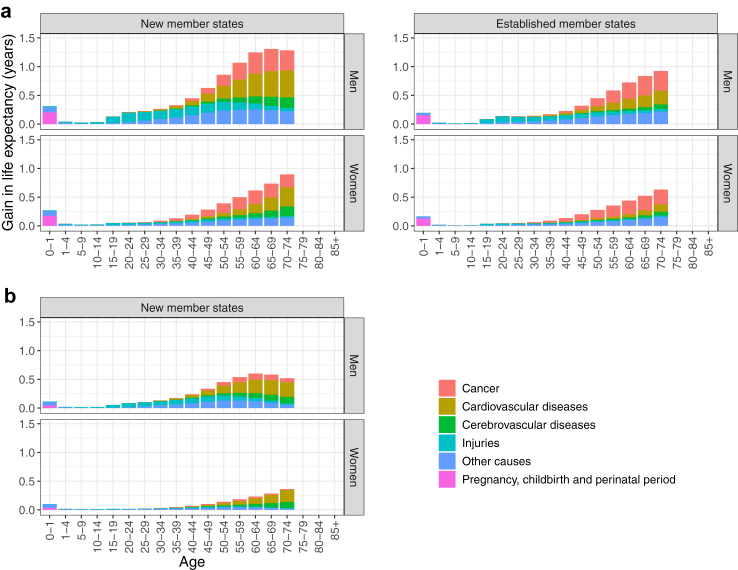
**The average contribution of selected cause groups to the estimated gains in life expectancy at birth (a) if all avoidable deaths were averted and (b) if new member states were assigned average avoidable mortality rates observed across the established member states, 2005–2019, by sex**.

Fig. 2 (panel a) illustrates the decomposition of the gains in life expectancy at birth by age and selected cause groups if all avoidable deaths were averted throughout the EU (see Supplementary Tables S5–S8 for a complete overview). Note that the relative impacts of age and cause categories were the same when decomposing the life expectancy gains and reduction in lifespan disparity in both scenarios. For this reason, we illustrate only the decomposition of life expectancy gains.

In both new and established member states and for both sexes, the reduction in avoidable cardiovascular, cerebrovascular, cancer, and injury deaths in middle and old age drives the observed changes in life expectancy. There are two main differences between the patterns of the two country groups. The first is the absolute number of years gained in each age group, which reflects the higher avoidable mortality rates at every age in new member states. The second is the relative proportions of years gained due to cardiovascular and cancer deaths avoided, where the former category dominates the latter in new member states, while the latter is marginally more important in the established member states, especially in women.

#### Scenario 2

Fig. 2 (panel b) illustrates the decomposition of the gains in life expectancy at birth by age and selected cause groups if new member states were assigned average avoidable mortality rates observed across the established member states. In this scenario, avoidable deaths due to cardiovascular disease in middle and older ages drive the vast majority of the simulated improvements in life expectancy and lifespan dispersion for both sexes. In other words, an average of 42% of the simulated 4 years of life expectancy gained in men and 53% of the 1.7 years in women would have been gained by achieving reductions in avoidable circulatory (i.e., cardiovascular and cerebrovascular) mortality already observed in established member states. The relevant proportions for the reduction in lifespan dispersion were 48% in women and 32% in men. Second place in order of impact for both sexes was reducing deaths due to injuries, responsible for 21% of the simulated gain in life expectancy in men and 11% in women, while they contributed to 27% and 13% of the simulated reduction in lifespan disparity.

However, it is critical to highlight that averaging across new member states hides important variations in the impact of groups of avoidable causes between individual countries. While the impact of averting avoidable cardiovascular and injury-related deaths in middle and older age is indeed critical in most new member states, averting alcohol- and drug-related mortality and cancer mortality would have been as or more impactful for both sexes in Slovenia and Hungary and for Croatian men (see Supplementary Tables S5–S8).

## Discussion

This study sought to quantify the potential health gains of reducing untimely and premature deaths in the European Union context. We analysed the gains in life expectancy and the reduction of lifespan disparity in the EU since the 2004 EU enlargement and how these changes would impact the mortality gaps between established and new EU member states. By applying two different scenarios, we identified three major patterns. First, all EU countries would gain substantial additional life expectancy if all avoidable causes of death were averted. However, the new member states would gain the most additional years of life expectancy, for example, 12.1 years for Lithuanian men and 5.8 years for Romanian women (see Supplementary Tables S1–S4 for country-specific results). Second, in both scenarios, the difference in life expectancy between established and new member states would be narrowed. Were all deaths averted, the life expectancy gap would be reduced by 1.3 years for women and 3.4 years for men. Were new member states as capable of averting avoidable deaths as established member states on average, the gap would be reduced by 1.7 years for women and 4 years for men. Likewise, lifespan disparities would be compressed in all EU member states, and the difference between established and new member states would be eliminated or reversed. Third, the main driving force for achieving these gains would be improvements in avoidable circulatory and injury-related mortality in middle and older ages.

The suggested life expectancy increases in this analysis highlight the central role of health systems’ performance in avoiding untimely and unnecessary deaths. This does not only refer to the appropriate treatment but also to the prevention of selected diseases, particularly for men. Earlier research has underlined the relevance of unfavourable health behaviours contributing to large life expectancy differentials across European countries. Alcohol consumption, smoking and increasing obesity levels are major behavioural risk factors that account for shorter average lifespans in Europe.^7^^,^^35^ A study by Janssen, Trias-Llimos & Kunst estimated that life expectancy at birth in Europe would be 5.8 years higher for men and 2.3 years for women if mortality attributable to smoking, alcohol consumption and obesity could be avoided.^36^ Previous research has suggested that ambitious tobacco control programs are related to lower initiation of smoking among adolescents,^37^ lower prevalence and consumption among adults^38^^,^^39^ and potential gains in life expectancy by reducing cancer and cardiovascular mortality.^40^, ^41^, ^42^ Still, there are pronounced differences in the implementation of such programs across European countries.^43^ Also, the documented successes of alcohol control programs for the reduction of all-cause mortality in a new EU member state like Lithuania suggests untapped potential to avoid premature mortality in new and established EU member states.^44^

Next to public health measures that would help to reduce preventable mortality, equal access to healthcare systems and modern treatments has a great potential to increase the average length of life for EU citizens.^45^ Over the past four decades, the availability of modern treatments has led to a steady decline in amenable mortality across EU countries and socioeconomic groups.^16^ Despite this overall progress, inequalities in levels and trends in treatable mortality remain between EU countries.^14^ Especially Europeans with lower socioeconomic status still exhibit a higher risk of death from treatable causes than those with a higher status.^18^

A central reason for the persisting disparities in life expectancy and avoidable mortality levels might be the differences in health system funding. Previous studies found an inverse relation between increases in healthcare expenditures and amenable mortality over time.^46^^,^^47^ Likewise, implementing successful public programs and campaigns to reduce preventable mortality might hinge on available funding.^48^ The difference in per capita healthcare spending between established and new member states is sizable. All Central and Eastern European member states that joined the EU after 2004 spent less per inhabitant than the EU average in 2021.^49^ These comparatively low numbers mask the increases in healthcare spending in the new member states in the last years. New member states like Estonia, Lithuania, and Romania have slightly narrowed the gap to the EU average by spending more than twice as much per capita on health care in 2021 than ten years earlier.^49^ Still, the absolute difference in this measure ranges from Luxemburg and Denmark, which spent more than €6000 per inhabitant in 2021, to Poland, Romania and Bulgaria, which spent less than €1000 in the same year. The results of our scenario in which the new member states could potentially reduce the gap in life expectancy and lifespan disparity by achieving the avoidable mortality rates of the established member states might partially reflect the effect of reducing the differences in healthcare spending. This seems especially relevant to remove the larger inequalities in average lifespans between countries. Even more important for narrowing life expectancy and health inequalities may be the overall public expenditures for dependent and disadvantaged population subgroups that go beyond the exclusive focus on healthcare.^50^, ^51^, ^52^ Also here, a large gap in public social spending between established and new EU member states exists and may explain why the gap in EU life expectancy is not entirely removed even by avoiding all treatable and preventable deaths.^53^ This suggests that the health care system can only partially narrow prevailing health inequalities in EU countries as the reasons for these inequalities are multifaceted. They range from the combination of lower education, precarious employment situations and lack of income to the living situation in disadvantaged neighbourhoods with higher crime rates, poorer housing and public infrastructure (see English Indices of Deprivation^54^ for an example). Also, classical risk factors for lower life expectancy are more prevalent among lower-educated population subgroups than higher-educated ones throughout Europe.^11^ Tackling these risk factors requires a comprehensive strategy that goes beyond the national healthcare system (see Marmot and Bell 2013^55^ for further details). Still, existing research also suggests that higher healthcare expenditures are related to declining treatable mortality among higher and lower-educated Europeans and contribute to the narrowing of inequalities of treatable mortality among both groups.^16^

Our results also suggest that resources should be targeted at the working and retirement-age population to avoid untimely and unnecessary deaths. Mortality reductions, especially for circulatory diseases, among women and men in these age groups would contribute most to reducing lifespan inequalities and narrowing the gap in life expectancy between EU member states. Circulatory diseases remain the most common cause of death in Europe and a major subject for prevention and treatment.^56^ Earlier research has suggested a combination of preventive cardiology and an increased focus on modifiable lifestyle factors such as nutrition and physical activity and the aforementioned smoking and alcohol consumption to tackle diabetes and hyperlipidaemia as primary risk factors for cardiovascular mortality in Europe.^57^^,^^58^ The slowing down of mortality improvements for these causes of death are responsible for lower life expectancy gains across European countries.^59^ The pattern of limited cardiovascular mortality improvements in working and retirement ages is quite alarming as it is also discussed as the root of stagnating life expectancy levels in the US and UK.^60^, ^61^, ^62^ Thus, addressing avoidable mortality is not only relevant for a continued rise and comparable life expectancy levels across EU member states but also to reduce inequalities in the lengths of lives of EU citizens.

This analysis contributes to existing literature in different ways. It quantifies to what extent life span disparities could be narrowed and life years could be gained by improving the performance of EU health systems. The two scenarios show that all EU member states would benefit from an ambitious goal to avoid all possible causes of death and new member states even from a more realistic goal of achieving the average mortality levels of the established member states. In the EU context, avoidable mortality has been mostly used as an indicator for evaluating trends in specific country settings, for comparisons between population subgroups or for impact assessment of specific healthcare and policy interventions. Its contribution to a potential convergence of average lengths of life and, most importantly, variations herein has received far less attention. Our novel results also show which specific age groups and causes of death should be addressed to meet the EU's objective of facilitating comparable health levels across its member states. Still, our results have certain limitations, primarily due to the characteristics of the avoidable mortality indicator itself. The life expectancy gap between established and new member states that remained even after removing all avoidable deaths may be caused by the upper age threshold of 74 years in the definition of avoidable deaths. During the last decades, large mortality improvements have been achieved in the ages above 74, which contributed substantially to the increases in life expectancy.^63^^,^^64^ Further convergence between member states may be achieved by improving mortality above age 75 in the new member states. Also, avoiding deaths as such may not be the most refined measure of health system performance. Delaying the onset or prolonging the survival times from certain diseases like dementia or cancers are successes of the health systems not captured by our indicator. Including these refinements is certainly a valuable addition for comparing relative health system performances.^65^ Still, our results concisely estimate how health policy could address inequalities between and within countries.

Twenty years after the 2004 EU enlargement, the life expectancy and lifespan disparity gaps between established and new member states remain large. Despite the EU's greater focus on health as a central dimension of living conditions, its role in shaping the finances and management of the healthcare system remains limited. The member states themselves are central in modernising their health system infrastructure and providing equal access to the best treatments. Our study suggests that a sizeable number of life years could be gained by changing political priority setting towards more preventive efforts and better treatment of diseases where premature death is unnecessary. This holds especially true for addressing health inequalities across EU member states. Taking these steps is certainly an ambitious goal at the EU level. However, focusing on facilitating prevention as set out in the EU4Health initiative might be a first step in avoiding untimely and premature deaths in the enlarged European Union.

## Contributors

Both authors contributed equally to all aspects of the study and the manuscript.

## Data sharing statement

Data on deaths by cause are available directly via the WHO mortality database (https://www.who.int/data/data-collection-tools/who-mortality-database).

## Declaration of interests

We declare no competing interests.
