# Supplementary material for: The contribution of avoidable mortality to life expectancy differences and lifespan disparities in the European Union: a population-based study
Source: Lancet Reg Health Eur. 2024 Aug 30;46:101042. doi: 10.1016/j.lanepe.2024.101042 (PMC11402299; doi:10.1016/j.lanepe.2024.101042)
Supplement: Supplementary material [file mmc1.pdf]

SUPPLEMENTARY MATERIAL

The contribution of avoidable mortality to life expectancy differences and lifespan disparities in the European Union

Rok Hrzic and Tobias Vogt

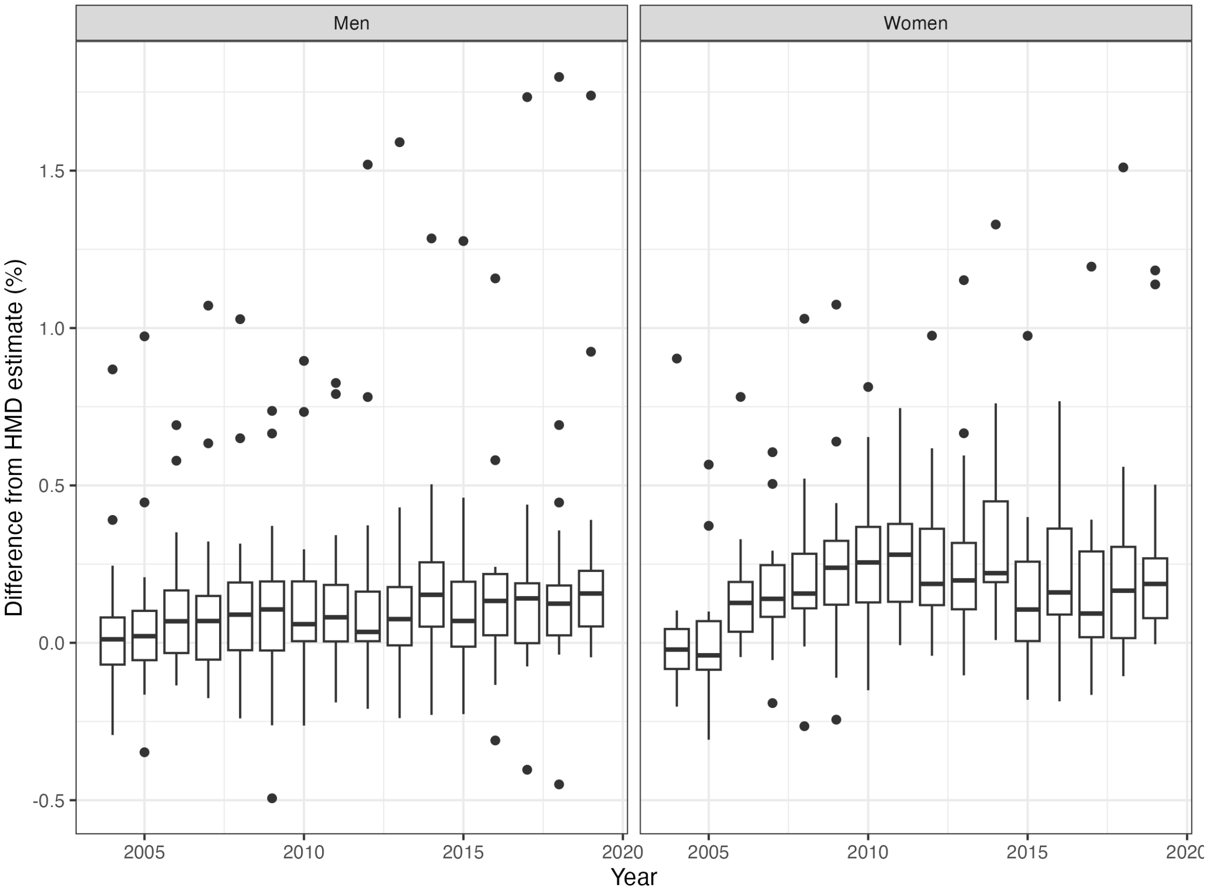

Supplementary Fig. 1: **Comparison of life expectancy at birth estimates in this study with the estimates from the Human Mortality Database.** Note: Most positive outliers are data points from Luxembourg.

Supplementary Table 1: Life expectancy gains in men under different scenarios, 2005-2019

Country	Status Quo	EMS avoidable mortality in NMS[a]	EMS preventable mortality in NMS[a]	EMS treatable mortality in NMS[a]	All avoidable deaths removed	Only preventable deaths removed	Only treatable deaths removed
Austria	78·34	0·00	0·00	0·00	5·47	3·83	1·42
Belgium	77·98	0·00	0·00	0·00	5·19	3·73	1·28
Bulgaria	70·55	4·02	1·95	1·89	8·06	4·43	2·95
Croatia	73·71	3·21	2·11	1·00	7·73	5·00	2·17
Cyprus	79·36	0·27	0·21	0·17	4·68	2·98	1·54
Czech Republic	74·93	2·40	1·38	0·95	6·94	4·31	2·16
Denmark	77·93	0·00	0·00	0·00	4·96	3·43	1·34
Estonia	70·54	5·98	4·25	1·43	10·54	7·10	2·49
Finland	77·46	0·00	0·00	0·00	6·49	4·67	1·52
France	78·46	0·00	0·00	0·00	5·13	3·76	1·21
Germany	78·09	0·00	0·00	0·00	5·20	3·43	1·55
Hungary	71·22	5·40	3·52	1·56	9·83	6·26	2·63
Ireland	78·91	0·00	0·00	0·00	4·93	3·30	1·44
Italy	79·76	0·00	0·00	0·00	4·39	2·95	1·30
Latvia	68·32	7·42	4·83	2·04	11·68	7·38	3·01
Lithuania	68·06	7·78	5·24	1·98	12·11	7·84	2·96
Luxembourg	79·56	0·00	0·00	0·00	4·65	3·23	1·25
Malta	78·69	0·47	0·19	0·39	4·98	3·01	1·77
Netherlands	79·27	0·00	0·00	0·00	4·02	2·67	1·22
Poland	72·56	3·35	2·14	1·10	7·69	4·92	2·24
Portugal	77·28	0·00	0·00	0·00	5·41	3·58	1·59
Romania	70·65	5·79	3·24	2·20	10·21	5·93	3·30
Slovakia	72·29	4·21	2·37	1·63	8·66	5·12	2·77
Slovenia	76·76	1·69	1·43	0·27	6·43	4·64	1·48
Spain	79·27	0·00	0·00	0·00	4·85	3·27	1·40
Sweden	79·86	0·00	0·00	0·00	4·20	2·80	1·27
United Kingdom	78·65	0·00	0·00	0·00	5·39	3·45	1·72
New member states	72·87	4·01	2·53	1·28	8·43	5·30	2·43
Established member states	78·65	0·00	0·00	0·00	5·01	3·43	1·39

[a]NMS = New member states, EMS = Established member states;

Supplementary Table 2: **Life expectancy gains in women under different scenarios, 2005-2019**

Country	Status Quo	EMS avoidable mortality in NMS[a]	EMS preventable mortality in NMS[a]	EMS treatable mortality in NMS[a]	All avoidable deaths removed	Only preventable deaths removed	Only treatable deaths removed
Austria	83·51	0·00	0·00	0·00	3·09	1·70	1·32
Belgium	83·15	0·00	0·00	0·00	3·30	1·79	1·43
Bulgaria	77·67	2·09	0·70	1·34	4·63	1·92	2·52
Croatia	80·24	1·19	0·56	0·64	3·91	1·88	1·89
Cyprus	83·70	0·10	0·08	0·08	2·39	1·06	1·29
Czech Republic	81·08	0·97	0·48	0·48	3·72	1·86	1·73
Denmark	81·99	0·00	0·00	0·00	3·59	2·07	1·41
Estonia	80·55	1·92	1·24	0·68	4·81	2·65	1·97
Finland	83·74	0·00	0·00	0·00	3·18	1·84	1·26
France	85·19	0·00	0·00	0·00	2·84	1·43	1·35
Germany	82·99	0·00	0·00	0·00	3·15	1·61	1·46
Hungary	78·71	2·75	1·62	1·06	5·48	2·93	2·27
Ireland	83·11	0·00	0·00	0·00	3·22	1·67	1·47
Italy	84·79	0·00	0·00	0·00	2·62	1·19	1·38
Latvia	78·44	2·97	1·59	1·31	5·70	2·89	2·52
Lithuania	79·14	2·72	1·54	1·13	5·50	2·88	2·37
Luxembourg	84·21	0·00	0·00	0·00	2·93	1·59	1·28
Malta	83·15	0·41	0·10	0·49	3·05	1·16	1·81
Netherlands	83·01	0·00	0·00	0·00	3·20	1·69	1·43
Poland	80·88	1·21	0·57	0·63	4·03	1·95	1·94
Portugal	83·63	0·00	0·00	0·00	2·82	1·26	1·50
Romania	77·94	3·12	1·40	1·63	5·80	2·67	2·84
Slovakia	79·69	1·74	0·72	1·00	4·47	2·03	2·26
Slovenia	83·18	0·40	0·33	0·13	3·27	1·78	1·40
Spain	85·33	0·00	0·00	0·00	2·43	1·10	1·29
Sweden	83·68	0·00	0·00	0·00	2·89	1·60	1·22
United Kingdom	82·54	0·00	0·00	0·00	3·66	1·88	1·67
New member states	80·31	1·67	0·84	0·82	4·37	2·13	2·07
Established member states	83·62	0·00	0·00	0·00	3·07	1·61	1·39

[a]NMS = New member states, EMS = Established member states;

***Supplementary Table 3:* Lifespan disparity reductions in men under different scenarios, 2005-2019**

Country	Status Quo	EMS avoidable mortality in NMS[a]	EMS preventable mortality in NMS[a]	EMS treatable mortality in NMS[a]	All avoidable deaths removed	Only preventable deaths removed	Only treatable deaths removed
Austria	16·01	0·00	0·00	0·00	-3·94	-2·66	-0·90
Belgium	16·11	0·00	0·00	0·00	-3·69	-2·57	-0·80
Bulgaria	17·28	-1·83	-0·80	-0·79	-4·31	-2·05	-1·29
Croatia	16·16	-1·62	-1·04	-0·42	-4·78	-2·85	-1·05
Cyprus	15·23	-0·22	-0·18	-0·12	-3·53	-2·17	-1·05
Czech Republic	16·20	-1·21	-0·70	-0·40	-4·38	-2·54	-1·09
Denmark	15·51	0·00	0·00	0·00	-3·47	-2·31	-0·83
Estonia	18·49	-2·95	-2·11	-0·43	-6·01	-3·73	-0·88
Finland	16·51	0·00	0·00	0·00	-4·67	-3·25	-0·91
France	17·19	0·00	0·00	0·00	-3·68	-2·62	-0·78
Germany	16·43	0·00	0·00	0·00	-3·60	-2·27	-0·95
Hungary	17·80	-2·37	-1·42	-0·51	-5·36	-2·97	-0·99
Ireland	15·38	0·00	0·00	0·00	-3·64	-2·37	-0·95
Italy	15·40	0·00	0·00	0·00	-3·28	-2·12	-0·89
Latvia	18·75	-3·32	-2·08	-0·54	-6·12	-3·39	-0·91
Lithuania	19·33	-3·57	-2·34	-0·51	-6·41	-3·68	-0·88
Luxembourg	15·30	0·00	0·00	0·00	-3·42	-2·31	-0·82
Malta	15·36	-0·31	-0·12	-0·27	-3·64	-2·08	-1·19
Netherlands	15·12	0·00	0·00	0·00	-2·90	-1·85	-0·83
Poland	18·30	-1·66	-1·05	-0·46	-4·39	-2·59	-1·01
Portugal	16·05	0·00	0·00	0·00	-3·80	-2·42	-0·99
Romania	18·25	-2·84	-1·40	-0·96	-5·84	-2·89	-1·48
Slovakia	17·29	-1·98	-1·06	-0·62	-5·00	-2·64	-1·20
Slovenia	16·41	-0·97	-0·85	-0·12	-4·37	-3·02	-0·82
Spain	16·35	0·00	0·00	0·00	-3·51	-2·27	-0·92
Sweden	14·70	0·00	0·00	0·00	-3·19	-2·07	-0·87
United Kingdom	16·37	0·00	0·00	0·00	-3·94	-2·41	-1·14
New member states	17·30	-1·91	-1·16	-0·47	-4·93	-2·81	-1·06
Established member states	15·87	0·00	0·00	0·00	-3·62	-2·39	-0·90

[a]NMS = New member states, EMS = Established member states;

Supplementary Table 4: **Lifespan disparity reductions in women under different scenarios, 2005-2019**

Country	Status Quo	EMS avoidable mortality in NMS[a]	EMS preventable mortality in NMS[a]	EMS treatable mortality in NMS[a]	All avoidable deaths removed	Only preventable deaths removed	Only treatable deaths removed
Austria	14·26	0·00	0·00	0·00	-2·58	-1·37	-1·06
Belgium	15·02	0·00	0·00	0·00	-2·72	-1·44	-1·13
Bulgaria	14·96	-1·39	-0·43	-0·89	-3·32	-1·26	-1·72
Croatia	13·96	-0·81	-0·36	-0·42	-3·00	-1·36	-1·37
Cyprus	13·37	-0·08	-0·07	-0·07	-2·00	-0·86	-1·05
Czech Republic	14·33	-0·65	-0·33	-0·31	-2·87	-1·37	-1·26
Denmark	15·07	0·00	0·00	0·00	-2·79	-1·54	-1·05
Estonia	15·61	-1·40	-0·92	-0·45	-3·75	-1·99	-1·40
Finland	14·56	0·00	0·00	0·00	-2·67	-1·52	-1·01
France	15·62	0·00	0·00	0·00	-2·43	-1·20	-1·13
Germany	14·41	0·00	0·00	0·00	-2·58	-1·28	-1·15
Hungary	15·82	-1·82	-1·03	-0·66	-3·98	-1·98	-1·51
Ireland	14·64	0·00	0·00	0·00	-2·63	-1·31	-1·16
Italy	14·45	0·00	0·00	0·00	-2·23	-0·98	-1·15
Latvia	15·90	-2·05	-1·09	-0·83	-4·22	-2·02	-1·70
Lithuania	15·86	-1·94	-1·09	-0·74	-4·18	-2·07	-1·64
Luxembourg	14·70	0·00	0·00	0·00	-2·43	-1·28	-1·02
Malta	14·71	-0·33	-0·08	-0·39	-2·49	-0·91	-1·45
Netherlands	14·81	0·00	0·00	0·00	-2·61	-1·32	-1·13
Poland	15·73	-0·85	-0·40	-0·44	-3·08	-1·42	-1·41
Portugal	14·12	0·00	0·00	0·00	-2·36	-1·03	-1·22
Romania	15·55	-2·13	-0·88	-1·09	-4·26	-1·78	-1·96
Slovakia	14·93	-1·17	-0·47	-0·65	-3·34	-1·42	-1·58
Slovenia	14·60	-0·30	-0·25	-0·09	-2·67	-1·41	-1·09
Spain	14·46	0·00	0·00	0·00	-2·10	-0·93	-1·09
Sweden	14·15	0·00	0·00	0·00	-2·40	-1·29	-0·97
United Kingdom	15·30	0·00	0·00	0·00	-2·95	-1·45	-1·30
New member states	15·03	-1·15	-0·57	-0·54	-3·32	-1·53	-1·47
Established member states	14·68	0·00	0·00	0·00	-2·54	-1·28	-1·11

[a]NMS = New member states, EMS = Established member states;

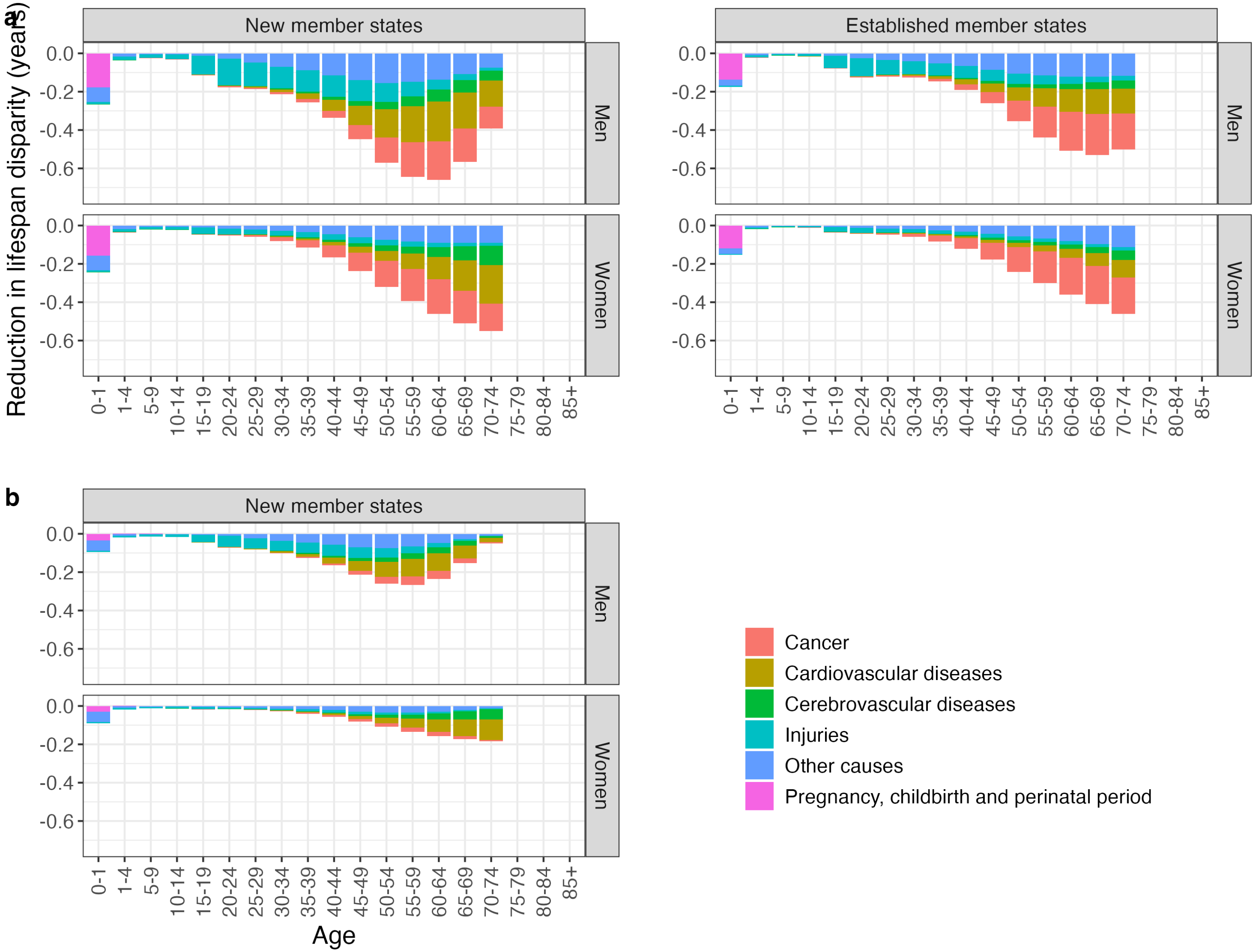

Supplementary Fig. 2: **The average contribution of cause groups to the estimated reductions in lifespan disparity (a) if all avoidable deaths were averted and (b) if new member states were assigned average avoidable mortality rates observed across the established member states, 2005-2019, by sex**

Supplementary Table 5: **The average contribution of cause groups to the estimated gains in male life expectancy by EU member state if all avoidable deaths were averted, 2005-2019**

Country	Cause Group	0-1	1-4	5-9	10-14	15-19	20-24	25-29	30-34	35-39	40-44	45-49	50-54	55-59	60-64	65-69	70-74
Austria	Adverse effects of medical and surgical care	0·00	0·00	0·00	0·00	0·00	0·00	0·00	0·00	0·00	0·00	0·00	0·00	0·00	0·00	0·00	0·00
	Alcohol- related and drug related deaths	0·00	0·00	0·00	0·00	0·02	0·03	0·03	0·03	0·04	0·04	0·07	0·08	0·10	0·10	0·08	0·06
	Cancer	0·00	0·00	0·00	0·00	0·00	0·00	0·01	0·01	0·02	0·03	0·07	0·14	0·23	0·30	0·33	0·32
	Cardiovascular diseases	0·00	0·00	0·00	0·00	0·00	0·00	0·00	0·01	0·02	0·03	0·06	0·10	0·15	0·21	0·26	0·32
	Cerebrovascular diseases	0·00	0·00	0·00	0·00	0·00	0·00	0·00	0·00	0·00	0·01	0·01	0·02	0·02	0·03	0·05	0·07
	Congenital malformations	0·03	0·00	0·00	0·00	0·00	0·00	0·00	0·00	0·00	0·00	0·00	0·00	0·00	0·00	0·00	0·00
	Diseases of the digestive system	0·00	0·00	0·00	0·00	0·00	0·00	0·00	0·00	0·00	0·00	0·00	0·01	0·01	0·01	0·01	0·01
	Diseases of the genitourinary system	0·00	0·00	0·00	0·00	0·00	0·00	0·00	0·00	0·00	0·00	0·00	0·00	0·00	0·01	0·01	0·01
	Diseases of the nervous system	0·00	0·00	0·00	0·00	0·00	0·00	0·00	0·00	0·00	0·00	0·00	0·00	0·00	0·00	0·00	0·00
	Diseases of the respiratory system	0·00	0·00	0·00	0·00	0·00	0·00	0·00	0·00	0·00	0·00	0·01	0·01	0·03	0·05	0·08	0·09
	Endocrine and metabolic diseases	0·00	0·00	0·00	0·00	0·00	0·00	0·00	0·00	0·00	0·00	0·01	0·01	0·02	0·04	0·05	0·06
	Infectious diseases	0·00	0·00	0·00	0·00	0·00	0·00	0·00	0·00	0·01	0·01	0·01	0·01	0·01	0·01	0·01	0·01
	Injuries	0·01	0·02	0·01	0·01	0·10	0·12	0·09	0·08	0·08	0·08	0·09	0·08	0·08	0·07	0·06	0·06
	Nonavoidable deaths	0·00	0·00	0·00	0·00	0·00	0·00	0·00	0·00	0·00	0·00	0·00	0·00	0·00	0·00	0·00	0·00
	Pregnancy, childbirth and perinatal period	0·16	0·00	0·00	0·00	0·00	0·00	0·00	0·00	0·00	0·00	0·00	0·00	0·00	0·00	0·00	0·00
Belgium	Adverse effects of medical and surgical care	0·00	0·00	0·00	0·00	0·00	0·00	0·00	0·00	0·00	0·00	0·00	0·00	0·00	0·00	0·00	0·00
	Alcohol- related and drug related deaths	0·00	0·00	0·00	0·00	0·00	0·01	0·02	0·02	0·03	0·04	0·05	0·06	0·07	0·06	0·05	0·03
	Cancer	0·00	0·00	0·00	0·00	0·00	0·00	0·01	0·01	0·02	0·03	0·07	0·14	0·23	0·32	0·37	0·36
	Cardiovascular diseases	0·00	0·00	0·00	0·00	0·00	0·00	0·00	0·01	0·01	0·02	0·04	0·07	0·10	0·13	0·16	0·18
	Cerebrovascular diseases	0·00	0·00	0·00	0·00	0·00	0·00	0·00	0·00	0·00	0·01	0·01	0·02	0·03	0·04	0·05	0·07
	Congenital malformations	0·03	0·00	0·00	0·00	0·00	0·00	0·00	0·00	0·00	0·00	0·00	0·00	0·00	0·00	0·00	0·00

Country	Cause Group	0-1	1-4	5-9	10-14	15-19	20-24	25-29	30-34	35-39	40-44	45-49	50-54	55-59	60-64	65-69	70-74
	Diseases of the digestive system	0·00	0·00	0·00	0·00	0·00	0·00	0·00	0·00	0·00	0·00	0·00	0·00	0·01	0·01	0·01	0·01
	Diseases of the genitourinary system	0·00	0·00	0·00	0·00	0·00	0·00	0·00	0·00	0·00	0·00	0·00	0·00	0·00	0·01	0·01	0·01
	Diseases of the nervous system	0·00	0·00	0·00	0·00	0·00	0·00	0·00	0·00	0·00	0·00	0·00	0·00	0·00	0·00	0·00	0·00
	Diseases of the respiratory system	0·00	0·00	0·00	0·00	0·00	0·00	0·00	0·00	0·00	0·01	0·01	0·03	0·05	0·07	0·11	0·14
	Endocrine and metabolic diseases	0·00	0·00	0·00	0·00	0·00	0·00	0·00	0·00	0·00	0·00	0·00	0·01	0·01	0·02	0·02	0·02
	Infectious diseases	0·01	0·01	0·00	0·00	0·00	0·00	0·00	0·00	0·00	0·01	0·01	0·01	0·01	0·01	0·02	0·02
	Injuries	0·01	0·02	0·01	0·02	0·08	0·14	0·13	0·12	0·11	0·11	0·11	0·10	0·09	0·07	0·06	0·05
	Nonavoidable deaths	0·00	0·00	0·00	0·00	0·00	0·00	0·00	0·00	0·00	0·00	0·00	0·00	0·00	0·00	0·00	0·00
	Pregnancy, childbirth and perinatal period	0·16	0·00	0·00	0·00	0·00	0·00	0·00	0·00	0·00	0·00	0·00	0·00	0·00	0·00	0·00	0·00
Bulgaria	Adverse effects of medical and surgical care	0·00	0·00	0·00	0·00	0·00	0·00	0·00	0·00	0·00	0·00	0·00	0·00	0·00	0·00	0·00	0·00
	Alcohol- related and drug related deaths	0·00	0·00	0·00	0·00	0·00	0·01	0·01	0·02	0·03	0·05	0·07	0·09	0·09	0·08	0·06	0·03
	Cancer	0·00	0·00	0·00	0·00	0·00	0·01	0·01	0·02	0·03	0·06	0·11	0·20	0·28	0·32	0·31	0·24
	Cardiovascular diseases	0·00	0·00	0·00	0·00	0·00	0·01	0·01	0·02	0·05	0·10	0·17	0·25	0·33	0·39	0·40	0·39
	Cerebrovascular diseases	0·01	0·00	0·00	0·00	0·00	0·00	0·01	0·01	0·02	0·04	0·07	0·11	0·16	0·22	0·28	0·31
	Congenital malformations	0·07	0·01	0·00	0·00	0·00	0·00	0·00	0·00	0·00	0·00	0·00	0·00	0·00	0·00	0·00	0·00
	Diseases of the digestive system	0·00	0·00	0·00	0·00	0·00	0·00	0·00	0·00	0·01	0·01	0·01	0·01	0·01	0·01	0·01	0·01
	Diseases of the genitourinary system	0·00	0·00	0·00	0·00	0·00	0·00	0·00	0·00	0·01	0·01	0·01	0·01	0·02	0·02	0·02	0·02
	Diseases of the nervous system	0·00	0·00	0·00	0·00	0·00	0·00	0·00	0·00	0·00	0·00	0·00	0·00	0·00	0·00	0·00	0·00
	Diseases of the respiratory system	0·08	0·02	0·01	0·01	0·01	0·01	0·01	0·01	0·01	0·02	0·03	0·04	0·05	0·06	0·06	0·06
	Endocrine and metabolic diseases	0·00	0·00	0·00	0·00	0·00	0·00	0·00	0·00	0·00	0·01	0·01	0·01	0·02	0·03	0·03	0·03
	Infectious diseases	0·02	0·01	0·00	0·00	0·00	0·00	0·01	0·01	0·01	0·01	0·01	0·01	0·02	0·01	0·01	0·01
	Injuries	0·02	0·02	0·02	0·03	0·10	0·13	0·11	0·10	0·09	0·09	0·09	0·09	0·08	0·06	0·04	0·03

Country	Cause Group	0-1	1-4	5-9	10-14	15-19	20-24	25-29	30-34	35-39	40-44	45-49	50-54	55-59	60-64	65-69	70-74
	Nonavoidable deaths	0·00	0·00	0·00	0·00	0·00	0·00	0·00	0·00	0·00	0·00	0·00	0·00	0·00	0·00	0·00	0·00
	Pregnancy, childbirth and perinatal period	0·30	0·00	0·00	0·00	0·00	0·00	0·00	0·00	0·00	0·00	0·00	0·00	0·00	0·00	0·00	0·00
Croatia	Adverse effects of medical and surgical care	0·00	0·00	0·00	0·00	0·00	0·00	0·00	0·00	0·00	0·00	0·00	0·00	0·00	0·00	0·00	0·00
	Alcohol- related and drug related deaths	0·00	0·00	0·00	0·00	0·00	0·01	0·02	0·03	0·04	0·06	0·09	0·11	0·12	0·11	0·10	0·07
	Cancer	0·00	0·00	0·00	0·00	0·00	0·01	0·01	0·02	0·03	0·06	0·12	0·24	0·36	0·44	0·45	0·41
	Cardiovascular diseases	0·00	0·00	0·00	0·00	0·00	0·00	0·00	0·01	0·03	0·06	0·12	0·18	0·25	0·31	0·36	0·39
	Cerebrovascular diseases	0·00	0·00	0·00	0·00	0·00	0·00	0·00	0·00	0·01	0·02	0·03	0·06	0·09	0·13	0·17	0·22
	Congenital malformations	0·04	0·00	0·00	0·00	0·00	0·00	0·00	0·00	0·00	0·00	0·00	0·00	0·00	0·00	0·00	0·00
	Diseases of the digestive system	0·00	0·00	0·00	0·00	0·00	0·00	0·00	0·00	0·00	0·00	0·01	0·01	0·01	0·01	0·01	0·02
	Diseases of the genitourinary system	0·00	0·00	0·00	0·00	0·00	0·00	0·00	0·00	0·00	0·00	0·00	0·00	0·01	0·01	0·01	0·01
	Diseases of the nervous system	0·00	0·00	0·00	0·00	0·00	0·00	0·00	0·00	0·00	0·01	0·01	0·01	0·00	0·00	0·00	0·00
	Diseases of the respiratory system	0·00	0·00	0·00	0·00	0·00	0·00	0·00	0·00	0·00	0·01	0·02	0·02	0·03	0·05	0·07	0·09
	Endocrine and metabolic diseases	0·00	0·00	0·00	0·00	0·00	0·00	0·00	0·00	0·00	0·00	0·01	0·01	0·02	0·04	0·05	0·05
	Infectious diseases	0·00	0·01	0·00	0·00	0·00	0·00	0·00	0·00	0·01	0·01	0·01	0·01	0·01	0·01	0·01	0·01
	Injuries	0·01	0·02	0·02	0·02	0·11	0·16	0·12	0·10	0·10	0·10	0·11	0·11	0·10	0·08	0·07	0·06
	Nonavoidable deaths	0·00	0·00	0·00	0·00	0·00	0·00	0·00	0·00	0·00	0·00	0·00	0·00	0·00	0·00	0·00	0·00
	Pregnancy, childbirth and perinatal period	0·21	0·00	0·00	0·00	0·00	0·00	0·00	0·00	0·00	0·00	0·00	0·00	0·00	0·00	0·00	0·00
Cyprus	Adverse effects of medical and surgical care	0·00	0·00	0·00	0·00	0·00	0·00	0·00	0·00	0·00	0·00	0·00	0·00	0·00	0·00	0·00	0·00
	Alcohol- related and drug related deaths	0·00	0·00	0·00	0·00	0·00	0·01	0·01	0·02	0·02	0·02	0·01	0·02	0·02	0·02	0·02	0·02
	Cancer	0·00	0·00	0·00	0·00	0·00	0·01	0·00	0·01	0·02	0·02	0·05	0·08	0·13	0·22	0·26	0·27
	Cardiovascular diseases	0·00	0·00	0·00	0·00	0·00	0·00	0·01	0·02	0·03	0·08	0·10	0·15	0·19	0·22	0·25	0·29
	Cerebrovascular diseases	0·00	0·00	0·00	0·00	0·00	0·00	0·00	0·01	0·00	0·01	0·01	0·02	0·02	0·04	0·05	0·08

Country	Cause Group	0-1	1-4	5-9	10-14	15-19	20-24	25-29	30-34	35-39	40-44	45-49	50-54	55-59	60-64	65-69	70-74
	Congenital malformations	0·02	0·00	0·00	0·00	0·00	0·00	0·00	0·00	0·00	0·00	0·00	0·00	0·00	0·00	0·00	0·00
	Diseases of the digestive system	0·00	0·00	0·00	0·00	0·00	0·00	0·00	0·00	0·00	0·00	0·00	0·00	0·00	0·01	0·01	0·01
	Diseases of the genitourinary system	0·00	0·00	0·00	0·00	0·00	0·00	0·00	0·00	0·00	0·00	0·00	0·00	0·01	0·01	0·02	0·02
	Diseases of the nervous system	0·00	0·00	0·00	0·00	0·00	0·00	0·00	0·00	0·00	0·00	0·00	0·00	0·00	0·00	0·00	0·00
	Diseases of the respiratory system	0·00	0·00	0·00	0·00	0·00	0·00	0·00	0·00	0·00	0·01	0·01	0·01	0·01	0·02	0·04	0·06
	Endocrine and metabolic diseases	0·00	0·00	0·00	0·00	0·00	0·00	0·00	0·00	0·00	0·00	0·01	0·01	0·03	0·04	0·07	0·10
	Infectious diseases	0·01	0·00	0·00	0·00	0·00	0·00	0·00	0·00	0·00	0·01	0·00	0·01	0·01	0·01	0·01	0·02
	Injuries	0·01	0·01	0·01	0·01	0·10	0·16	0·12	0·10	0·09	0·08	0·07	0·05	0·04	0·04	0·04	0·04
	Nonavoidable deaths	0·00	0·00	0·00	0·00	0·00	0·00	0·00	0·00	0·00	0·00	0·00	0·00	0·00	0·00	0·00	0·00
	Pregnancy, childbirth and perinatal period	0·17	0·00	0·00	0·00	0·00	0·00	0·00	0·00	0·00	0·00	0·00	0·00	0·00	0·00	0·00	0·00
Czech Republic	Adverse effects of medical and surgical care	0·00	0·00	0·00	0·00	0·00	0·00	0·00	0·00	0·00	0·00	0·00	0·00	0·00	0·00	0·00	0·00
	Alcohol- related and drug related deaths	0·00	0·00	0·00	0·00	0·00	0·01	0·01	0·02	0·03	0·05	0·08	0·09	0·09	0·08	0·06	0·03
	Cancer	0·00	0·00	0·00	0·00	0·00	0·00	0·01	0·01	0·02	0·04	0·08	0·15	0·26	0·36	0·40	0·36
	Cardiovascular diseases	0·00	0·00	0·00	0·00	0·00	0·00	0·00	0·01	0·02	0·05	0·10	0·17	0·26	0·36	0·43	0·48
	Cerebrovascular diseases	0·00	0·00	0·00	0·00	0·00	0·00	0·00	0·00	0·01	0·01	0·02	0·03	0·05	0·07	0·10	0·13
	Congenital malformations	0·02	0·00	0·00	0·00	0·00	0·00	0·00	0·00	0·00	0·00	0·00	0·00	0·00	0·00	0·00	0·00
	Diseases of the digestive system	0·00	0·00	0·00	0·00	0·00	0·00	0·00	0·00	0·00	0·01	0·01	0·01	0·02	0·02	0·02	0·02
	Diseases of the genitourinary system	0·00	0·00	0·00	0·00	0·00	0·00	0·00	0·00	0·00	0·00	0·00	0·00	0·01	0·01	0·01	0·01
	Diseases of the nervous system	0·00	0·00	0·00	0·00	0·00	0·00	0·00	0·00	0·00	0·00	0·00	0·00	0·00	0·00	0·00	0·00
	Diseases of the respiratory system	0·01	0·00	0·00	0·00	0·00	0·01	0·01	0·01	0·01	0·01	0·02	0·04	0·06	0·08	0·10	0·11
	Endocrine and metabolic diseases	0·00	0·00	0·00	0·00	0·00	0·00	0·00	0·00	0·00	0·00	0·01	0·01	0·02	0·03	0·04	0·05
	Infectious diseases	0·00	0·00	0·00	0·00	0·00	0·00	0·00	0·00	0·00	0·00	0·01	0·01	0·01	0·01	0·02	0·02

Country	Cause Group	0-1	1-4	5-9	10-14	15-19	20-24	25-29	30-34	35-39	40-44	45-49	50-54	55-59	60-64	65-69	70-74
	Injuries	0·01	0·01	0·01	0·02	0·10	0·15	0·13	0·11	0·11	0·11	0·11	0·12	0·10	0·08	0·06	0·05
	Nonavoidable deaths	0·00	0·00	0·00	0·00	0·00	0·00	0·00	0·00	0·00	0·00	0·00	0·00	0·00	0·00	0·00	0·00
	Pregnancy, childbirth and perinatal period	0·14	0·00	0·00	0·00	0·00	0·00	0·00	0·00	0·00	0·00	0·00	0·00	0·00	0·00	0·00	0·00
Denmark	Adverse effects of medical and surgical care	0·00	0·00	0·00	0·00	0·00	0·00	0·00	0·00	0·00	0·00	0·00	0·00	0·00	0·00	0·00	0·00
	Alcohol- related and drug related deaths	0·00	0·00	0·00	0·00	0·01	0·02	0·03	0·04	0·05	0·07	0·10	0·14	0·14	0·12	0·08	0·05
	Cancer	0·00	0·00	0·00	0·00	0·00	0·00	0·01	0·01	0·02	0·03	0·06	0·12	0·20	0·28	0·34	0·35
	Cardiovascular diseases	0·00	0·00	0·00	0·00	0·00	0·00	0·00	0·01	0·01	0·02	0·04	0·07	0·09	0·13	0·16	0·20
	Cerebrovascular diseases	0·00	0·00	0·00	0·00	0·00	0·00	0·00	0·00	0·00	0·01	0·01	0·02	0·03	0·04	0·06	0·08
	Congenital malformations	0·02	0·00	0·00	0·00	0·00	0·00	0·00	0·00	0·00	0·00	0·00	0·00	0·00	0·00	0·00	0·00
	Diseases of the digestive system	0·00	0·00	0·00	0·00	0·00	0·00	0·00	0·00	0·00	0·00	0·00	0·01	0·01	0·01	0·01	0·01
	Diseases of the genitourinary system	0·00	0·00	0·00	0·00	0·00	0·00	0·00	0·00	0·00	0·00	0·00	0·00	0·00	0·01	0·01	0·01
	Diseases of the nervous system	0·00	0·00	0·00	0·00	0·00	0·00	0·00	0·00	0·00	0·00	0·00	0·00	0·00	0·00	0·00	0·00
	Diseases of the respiratory system	0·00	0·00	0·00	0·00	0·00	0·00	0·00	0·00	0·00	0·01	0·01	0·03	0·04	0·07	0·09	0·12
	Endocrine and metabolic diseases	0·00	0·00	0·00	0·00	0·00	0·00	0·00	0·00	0·00	0·01	0·01	0·02	0·03	0·03	0·04	0·05
	Infectious diseases	0·00	0·00	0·00	0·00	0·00	0·00	0·00	0·00	0·00	0·00	0·01	0·01	0·01	0·01	0·01	0·01
	Injuries	0·01	0·01	0·01	0·01	0·06	0·07	0·06	0·05	0·05	0·06	0·06	0·06	0·05	0·04	0·03	0·03
	Nonavoidable deaths	0·00	0·00	0·00	0·00	0·00	0·00	0·00	0·00	0·00	0·00	0·00	0·00	0·00	0·00	0·00	0·00
	Pregnancy, childbirth and perinatal period	0·16	0·00	0·00	0·00	0·00	0·00	0·00	0·00	0·00	0·00	0·00	0·00	0·00	0·00	0·00	0·00
Estonia	Adverse effects of medical and surgical care	0·00	0·00	0·00	0·00	0·00	0·00	0·00	0·00	0·00	0·00	0·00	0·00	0·00	0·00	0·00	0·00
	Alcohol- related and drug related deaths	0·00	0·00	0·00	0·00	0·02	0·09	0·17	0·19	0·16	0·19	0·22	0·26	0·24	0·18	0·12	0·06
	Cancer	0·00	0·00	0·00	0·00	0·00	0·00	0·01	0·01	0·02	0·04	0·09	0·16	0·26	0·36	0·40	0·38
	Cardiovascular diseases	0·00	0·00	0·00	0·00	0·00	0·01	0·01	0·02	0·03	0·07	0·16	0·26	0·40	0·55	0·62	0·67

Country	Cause Group	0-1	1-4	5-9	10-14	15-19	20-24	25-29	30-34	35-39	40-44	45-49	50-54	55-59	60-64	65-69	70-74
	Cerebrovascular diseases	0·00	0·00	0·00	0·00	0·00	0·01	0·01	0·01	0·01	0·02	0·04	0·05	0·07	0·11	0·14	0·15
	Congenital malformations	0·04	0·01	0·00	0·00	0·00	0·00	0·00	0·00	0·00	0·00	0·00	0·00	0·00	0·00	0·00	0·00
	Diseases of the digestive system	0·00	0·00	0·00	0·00	0·00	0·00	0·00	0·01	0·01	0·01	0·02	0·02	0·03	0·02	0·02	0·02
	Diseases of the genitourinary system	0·00	0·00	0·00	0·00	0·00	0·00	0·00	0·00	0·00	0·00	0·00	0·00	0·00	0·00	0·01	0·01
	Diseases of the nervous system	0·00	0·00	0·00	0·00	0·01	0·00	0·00	0·01	0·01	0·01	0·01	0·02	0·01	0·01	0·00	0·00
	Diseases of the respiratory system	0·01	0·01	0·00	0·00	0·00	0·00	0·01	0·02	0·01	0·03	0·04	0·05	0·06	0·07	0·07	0·07
	Endocrine and metabolic diseases	0·00	0·00	0·00	0·00	0·00	0·00	0·00	0·00	0·01	0·01	0·01	0·01	0·01	0·02	0·02	0·02
	Infectious diseases	0·01	0·00	0·00	0·00	0·00	0·01	0·04	0·05	0·04	0·03	0·03	0·02	0·02	0·01	0·01	0·01
	Injuries	0·03	0·02	0·03	0·03	0·14	0·23	0·20	0·18	0·18	0·20	0·21	0·21	0·19	0·13	0·09	0·06
	Nonavoidable deaths	0·00	0·00	0·00	0·00	0·00	0·00	0·00	0·00	0·00	0·00	0·00	0·00	0·00	0·00	0·00	0·00
	Pregnancy, childbirth and perinatal period	0·13	0·00	0·00	0·00	0·00	0·00	0·00	0·00	0·00	0·00	0·00	0·00	0·00	0·00	0·00	0·00
Finland	Adverse effects of medical and surgical care	0·00	0·00	0·00	0·00	0·00	0·00	0·00	0·00	0·00	0·00	0·00	0·00	0·00	0·00	0·00	0·00
	Alcohol- related and drug related deaths	0·00	0·00	0·00	0·00	0·02	0·06	0·07	0·09	0·11	0·13	0·18	0·21	0·22	0·18	0·13	0·09
	Cancer	0·00	0·00	0·00	0·00	0·00	0·01	0·01	0·01	0·01	0·03	0·04	0·08	0·15	0·22	0·27	0·28
	Cardiovascular diseases	0·00	0·00	0·00	0·00	0·00	0·00	0·00	0·01	0·02	0·04	0·07	0·13	0·21	0·29	0·36	0·40
	Cerebrovascular diseases	0·00	0·00	0·00	0·00	0·00	0·00	0·00	0·00	0·01	0·01	0·02	0·03	0·04	0·06	0·08	0·10
	Congenital malformations	0·02	0·00	0·00	0·00	0·00	0·00	0·00	0·00	0·00	0·00	0·00	0·00	0·00	0·00	0·00	0·00
	Diseases of the digestive system	0·00	0·00	0·00	0·00	0·00	0·00	0·00	0·00	0·01	0·01	0·01	0·02	0·02	0·02	0·02	0·01
	Diseases of the genitourinary system	0·00	0·00	0·00	0·00	0·00	0·00	0·00	0·00	0·00	0·00	0·00	0·00	0·00	0·00	0·00	0·00
	Diseases of the nervous system	0·00	0·00	0·00	0·00	0·00	0·00	0·00	0·00	0·00	0·00	0·01	0·01	0·00	0·00	0·00	0·00
	Diseases of the respiratory system	0·00	0·00	0·00	0·00	0·00	0·00	0·00	0·00	0·00	0·01	0·01	0·01	0·03	0·04	0·06	0·07
	Endocrine and metabolic diseases	0·00	0·00	0·00	0·00	0·00	0·00	0·00	0·00	0·00	0·01	0·01	0·01	0·01	0·01	0·02	0·02

Country	Cause Group	0-1	1-4	5-9	10-14	15-19	20-24	25-29	30-34	35-39	40-44	45-49	50-54	55-59	60-64	65-69	70-74
	Infectious diseases	0·00	0·00	0·00	0·00	0·00	0·00	0·00	0·00	0·00	0·00	0·00	0·00	0·00	0·01	0·01	0·01
	Injuries	0·00	0·01	0·01	0·01	0·11	0·15	0·13	0·11	0·10	0·10	0·11	0·11	0·10	0·09	0·08	0·07
	Nonavoidable deaths	0·00	0·00	0·00	0·00	0·00	0·00	0·00	0·00	0·00	0·00	0·00	0·00	0·00	0·00	0·00	0·00
	Pregnancy, childbirth and perinatal period	0·09	0·00	0·00	0·00	0·00	0·00	0·00	0·00	0·00	0·00	0·00	0·00	0·00	0·00	0·00	0·00
France	Adverse effects of medical and surgical care	0·00	0·00	0·00	0·00	0·00	0·00	0·00	0·00	0·00	0·00	0·00	0·00	0·00	0·00	0·00	0·00
	Alcohol- related and drug related deaths	0·00	0·00	0·00	0·00	0·00	0·01	0·01	0·02	0·03	0·05	0·07	0·08	0·08	0·07	0·05	0·04
	Cancer	0·00	0·00	0·00	0·00	0·00	0·00	0·01	0·01	0·02	0·05	0·11	0·22	0·31	0·37	0·38	0·37
	Cardiovascular diseases	0·00	0·00	0·00	0·00	0·00	0·00	0·00	0·01	0·01	0·03	0·04	0·06	0·09	0·10	0·12	0·14
	Cerebrovascular diseases	0·00	0·00	0·00	0·00	0·00	0·00	0·00	0·00	0·01	0·01	0·01	0·02	0·03	0·03	0·04	0·06
	Congenital malformations	0·03	0·00	0·00	0·00	0·00	0·00	0·00	0·00	0·00	0·00	0·00	0·00	0·00	0·00	0·00	0·00
	Diseases of the digestive system	0·00	0·00	0·00	0·00	0·00	0·00	0·00	0·00	0·00	0·00	0·00	0·00	0·01	0·01	0·01	0·01
	Diseases of the genitourinary system	0·00	0·00	0·00	0·00	0·00	0·00	0·00	0·00	0·00	0·00	0·00	0·00	0·00	0·01	0·01	0·01
	Diseases of the nervous system	0·00	0·00	0·00	0·00	0·00	0·00	0·00	0·00	0·00	0·00	0·00	0·00	0·00	0·00	0·00	0·00
	Diseases of the respiratory system	0·00	0·00	0·00	0·00	0·00	0·00	0·00	0·00	0·00	0·01	0·01	0·02	0·02	0·03	0·04	0·06
	Endocrine and metabolic diseases	0·00	0·00	0·00	0·00	0·00	0·00	0·00	0·00	0·00	0·00	0·01	0·01	0·01	0·02	0·03	0·03
	Infectious diseases	0·01	0·00	0·00	0·00	0·00	0·00	0·00	0·00	0·01	0·01	0·01	0·01	0·01	0·01	0·02	0·02
	Injuries	0·01	0·02	0·01	0·01	0·08	0·13	0·11	0·10	0·10	0·11	0·11	0·10	0·08	0·06	0·06	0·06
	Nonavoidable deaths	0·00	0·00	0·00	0·00	0·00	0·00	0·00	0·00	0·00	0·00	0·00	0·00	0·00	0·00	0·00	0·00
	Pregnancy, childbirth and perinatal period	0·16	0·00	0·00	0·00	0·00	0·00	0·00	0·00	0·00	0·00	0·00	0·00	0·00	0·00	0·00	0·00
Germany	Adverse effects of medical and surgical care	0·00	0·00	0·00	0·00	0·00	0·00	0·00	0·00	0·00	0·00	0·00	0·00	0·00	0·00	0·00	0·00
	Alcohol- related and drug related deaths	0·00	0·00	0·00	0·00	0·00	0·01	0·02	0·03	0·04	0·05	0·07	0·09	0·09	0·09	0·07	0·05
	Cancer	0·00	0·00	0·00	0·00	0·00	0·00	0·01	0·01	0·02	0·03	0·07	0·14	0·23	0·30	0·33	0·32

Country	Cause Group	0-1	1-4	5-9	10-14	15-19	20-24	25-29	30-34	35-39	40-44	45-49	50-54	55-59	60-64	65-69	70-74
	Cardiovascular diseases	0·00	0·00	0·00	0·00	0·00	0·00	0·00	0·01	0·02	0·04	0·07	0·11	0·16	0·21	0·25	0·30
	Cerebrovascular diseases	0·00	0·00	0·00	0·00	0·00	0·00	0·00	0·00	0·00	0·01	0·01	0·02	0·03	0·04	0·06	0·08
	Congenital malformations	0·03	0·00	0·00	0·00	0·00	0·00	0·00	0·00	0·00	0·00	0·00	0·00	0·00	0·00	0·00	0·00
	Diseases of the digestive system	0·00	0·00	0·00	0·00	0·00	0·00	0·00	0·00	0·00	0·00	0·00	0·01	0·01	0·01	0·01	0·01
	Diseases of the genitourinary system	0·00	0·00	0·00	0·00	0·00	0·00	0·00	0·00	0·00	0·00	0·00	0·00	0·00	0·01	0·01	0·02
	Diseases of the nervous system	0·00	0·00	0·00	0·00	0·00	0·00	0·00	0·00	0·00	0·00	0·00	0·00	0·00	0·00	0·00	0·00
	Diseases of the respiratory system	0·00	0·00	0·00	0·00	0·00	0·00	0·00	0·00	0·00	0·01	0·01	0·02	0·04	0·06	0·09	0·11
	Endocrine and metabolic diseases	0·00	0·00	0·00	0·00	0·00	0·00	0·00	0·00	0·00	0·00	0·01	0·01	0·02	0·03	0·03	0·04
	Infectious diseases	0·00	0·00	0·00	0·00	0·00	0·00	0·00	0·00	0·01	0·01	0·01	0·01	0·01	0·02	0·02	0·02
	Injuries	0·01	0·01	0·01	0·01	0·07	0·09	0·07	0·06	0·05	0·06	0·06	0·06	0·05	0·05	0·04	0·04
	Nonavoidable deaths	0·00	0·00	0·00	0·00	0·00	0·00	0·00	0·00	0·00	0·00	0·00	0·00	0·00	0·00	0·00	0·00
	Pregnancy, childbirth and perinatal period	0·16	0·00	0·00	0·00	0·00	0·00	0·00	0·00	0·00	0·00	0·00	0·00	0·00	0·00	0·00	0·00
Hungary	Adverse effects of medical and surgical care	0·00	0·00	0·00	0·00	0·00	0·00	0·00	0·00	0·00	0·00	0·00	0·00	0·00	0·00	0·00	0·00
	Alcohol- related and drug related deaths	0·00	0·00	0·00	0·00	0·00	0·01	0·01	0·03	0·05	0·10	0·17	0·21	0·19	0·17	0·12	0·07
	Cancer	0·00	0·00	0·00	0·00	0·00	0·01	0·01	0·01	0·03	0·07	0·18	0·35	0·49	0·54	0·50	0·40
	Cardiovascular diseases	0·00	0·00	0·00	0·00	0·00	0·00	0·01	0·02	0·04	0·09	0·18	0·29	0·40	0·49	0·53	0·55
	Cerebrovascular diseases	0·00	0·00	0·00	0·00	0·00	0·00	0·00	0·00	0·01	0·03	0·04	0·07	0·10	0·13	0·15	0·17
	Congenital malformations	0·05	0·00	0·00	0·00	0·00	0·00	0·00	0·00	0·00	0·00	0·00	0·00	0·00	0·00	0·00	0·00
	Diseases of the digestive system	0·00	0·00	0·00	0·00	0·00	0·00	0·00	0·00	0·01	0·01	0·02	0·02	0·03	0·03	0·03	0·02
	Diseases of the genitourinary system	0·00	0·00	0·00	0·00	0·00	0·00	0·00	0·00	0·00	0·00	0·00	0·00	0·00	0·00	0·01	0·01
	Diseases of the nervous system	0·00	0·00	0·00	0·00	0·00	0·00	0·00	0·00	0·00	0·00	0·00	0·00	0·00	0·00	0·00	0·00
	Diseases of the respiratory system	0·01	0·01	0·00	0·00	0·00	0·00	0·01	0·01	0·01	0·02	0·04	0·06	0·09	0·11	0·11	0·12

Country	Cause Group	0-1	1-4	5-9	10-14	15-19	20-24	25-29	30-34	35-39	40-44	45-49	50-54	55-59	60-64	65-69	70-74
	Endocrine and metabolic diseases	0·00	0·00	0·00	0·00	0·00	0·00	0·00	0·00	0·00	0·01	0·01	0·02	0·03	0·04	0·04	0·04
	Infectious diseases	0·01	0·00	0·00	0·00	0·00	0·00	0·00	0·00	0·00	0·01	0·01	0·01	0·01	0·01	0·01	0·01
	Injuries	0·01	0·02	0·01	0·02	0·07	0·10	0·10	0·11	0·11	0·13	0·15	0·15	0·13	0·10	0·08	0·06
	Nonavoidable deaths	0·00	0·00	0·00	0·00	0·00	0·00	0·00	0·00	0·00	0·00	0·00	0·00	0·00	0·00	0·00	0·00
	Pregnancy, childbirth and perinatal period	0·23	0·00	0·00	0·00	0·00	0·00	0·00	0·00	0·00	0·00	0·00	0·00	0·00	0·00	0·00	0·00
Ireland	Adverse effects of medical and surgical care	0·00	0·00	0·00	0·00	0·00	0·00	0·00	0·00	0·00	0·00	0·00	0·00	0·00	0·00	0·00	0·00
	Alcohol- related and drug related deaths	0·00	0·00	0·00	0·00	0·01	0·03	0·04	0·05	0·05	0·06	0·05	0·05	0·05	0·04	0·03	0·02
	Cancer	0·00	0·00	0·00	0·00	0·00	0·00	0·01	0·01	0·02	0·04	0·06	0·11	0·18	0·26	0·32	0·35
	Cardiovascular diseases	0·00	0·00	0·00	0·00	0·00	0·00	0·00	0·01	0·02	0·04	0·07	0·11	0·16	0·20	0·26	0·31
	Cerebrovascular diseases	0·00	0·00	0·00	0·00	0·00	0·00	0·00	0·00	0·01	0·01	0·01	0·02	0·02	0·03	0·04	0·06
	Congenital malformations	0·04	0·00	0·00	0·00	0·00	0·00	0·00	0·00	0·00	0·00	0·00	0·00	0·00	0·00	0·00	0·00
	Diseases of the digestive system	0·00	0·00	0·00	0·00	0·00	0·00	0·00	0·00	0·00	0·00	0·00	0·00	0·01	0·01	0·01	0·01
	Diseases of the genitourinary system	0·00	0·00	0·00	0·00	0·00	0·00	0·00	0·00	0·00	0·00	0·00	0·00	0·00	0·01	0·01	0·01
	Diseases of the nervous system	0·00	0·00	0·00	0·00	0·00	0·00	0·00	0·00	0·00	0·00	0·00	0·00	0·00	0·00	0·00	0·00
	Diseases of the respiratory system	0·00	0·00	0·00	0·00	0·00	0·00	0·00	0·00	0·00	0·01	0·01	0·02	0·03	0·05	0·07	0·12
	Endocrine and metabolic diseases	0·00	0·00	0·00	0·00	0·00	0·00	0·00	0·00	0·00	0·00	0·00	0·00	0·01	0·01	0·02	0·03
	Infectious diseases	0·00	0·00	0·00	0·00	0·00	0·00	0·00	0·00	0·00	0·01	0·01	0·00	0·01	0·01	0·01	0·01
	Injuries	0·00	0·01	0·00	0·02	0·08	0·13	0·10	0·08	0·08	0·08	0·07	0·07	0·06	0·05	0·04	0·03
	Nonavoidable deaths	0·00	0·00	0·00	0·00	0·00	0·00	0·00	0·00	0·00	0·00	0·00	0·00	0·00	0·00	0·00	0·00
	Pregnancy, childbirth and perinatal period	0·13	0·00	0·00	0·00	0·00	0·00	0·00	0·00	0·00	0·00	0·00	0·00	0·00	0·00	0·00	0·00
Italy	Adverse effects of medical and surgical care	0·00	0·00	0·00	0·00	0·00	0·00	0·00	0·00	0·00	0·00	0·00	0·00	0·00	0·00	0·00	0·00
	Alcohol- related and drug related deaths	0·00	0·00	0·00	0·00	0·00	0·00	0·01	0·01	0·01	0·02	0·02	0·03	0·03	0·03	0·03	0·03

Country	Cause Group	0-1	1-4	5-9	10-14	15-19	20-24	25-29	30-34	35-39	40-44	45-49	50-54	55-59	60-64	65-69	70-74
	Cancer	0·00	0·00	0·00	0·00	0·00	0·01	0·01	0·01	0·02	0·04	0·07	0·13	0·21	0·30	0·37	0·39
	Cardiovascular diseases	0·00	0·00	0·00	0·00	0·00	0·00	0·00	0·01	0·01	0·03	0·05	0·07	0·11	0·14	0·17	0·21
	Cerebrovascular diseases	0·00	0·00	0·00	0·00	0·00	0·00	0·00	0·00	0·00	0·01	0·01	0·02	0·02	0·04	0·05	0·08
	Congenital malformations	0·04	0·00	0·00	0·00	0·00	0·00	0·00	0·00	0·00	0·00	0·00	0·00	0·00	0·00	0·00	0·00
	Diseases of the digestive system	0·00	0·00	0·00	0·00	0·00	0·00	0·00	0·00	0·00	0·00	0·00	0·00	0·00	0·01	0·01	0·01
	Diseases of the genitourinary system	0·00	0·00	0·00	0·00	0·00	0·00	0·00	0·00	0·00	0·00	0·00	0·00	0·00	0·01	0·01	0·01
	Diseases of the nervous system	0·00	0·00	0·00	0·00	0·00	0·00	0·00	0·00	0·00	0·00	0·00	0·00	0·00	0·00	0·00	0·00
	Diseases of the respiratory system	0·00	0·00	0·00	0·00	0·00	0·00	0·00	0·00	0·00	0·01	0·01	0·01	0·02	0·02	0·04	0·06
	Endocrine and metabolic diseases	0·00	0·00	0·00	0·00	0·00	0·00	0·00	0·00	0·00	0·00	0·01	0·01	0·02	0·03	0·04	0·05
	Infectious diseases	0·00	0·00	0·00	0·00	0·00	0·00	0·00	0·00	0·01	0·01	0·02	0·02	0·02	0·01	0·02	0·02
	Injuries	0·00	0·01	0·01	0·01	0·07	0·10	0·09	0·08	0·06	0·06	0·06	0·05	0·04	0·04	0·03	0·03
	Nonavoidable deaths	0·00	0·00	0·00	0·00	0·00	0·00	0·00	0·00	0·00	0·00	0·00	0·00	0·00	0·00	0·00	0·00
	Pregnancy, childbirth and perinatal period	0·16	0·00	0·00	0·00	0·00	0·00	0·00	0·00	0·00	0·00	0·00	0·00	0·00	0·00	0·00	0·00
Latvia	Adverse effects of medical and surgical care	0·00	0·00	0·00	0·00	0·00	0·00	0·00	0·00	0·00	0·00	0·00	0·00	0·00	0·00	0·00	0·00
	Alcohol- related and drug related deaths	0·00	0·00	0·00	0·00	0·00	0·03	0·07	0·12	0·16	0·20	0·22	0·22	0·18	0·14	0·08	0·04
	Cancer	0·00	0·00	0·00	0·00	0·00	0·01	0·01	0·02	0·02	0·05	0·10	0·19	0·29	0·37	0·38	0·34
	Cardiovascular diseases	0·00	0·00	0·00	0·00	0·00	0·00	0·01	0·03	0·05	0·12	0·21	0·35	0·49	0·62	0·66	0·62
	Cerebrovascular diseases	0·00	0·00	0·00	0·00	0·00	0·00	0·01	0·01	0·02	0·03	0·06	0·09	0·14	0·20	0·25	0·27
	Congenital malformations	0·04	0·00	0·00	0·00	0·00	0·00	0·00	0·00	0·00	0·00	0·00	0·00	0·00	0·00	0·00	0·00
	Diseases of the digestive system	0·00	0·00	0·00	0·00	0·00	0·00	0·01	0·01	0·01	0·02	0·02	0·03	0·02	0·02	0·02	0·02
	Diseases of the genitourinary system	0·00	0·00	0·00	0·00	0·00	0·00	0·00	0·00	0·00	0·00	0·00	0·00	0·01	0·01	0·01	0·01
	Diseases of the nervous system	0·00	0·00	0·00	0·00	0·00	0·00	0·01	0·01	0·01	0·01	0·01	0·01	0·01	0·00	0·00	0·00

Country	Cause Group	0-1	1-4	5-9	10-14	15-19	20-24	25-29	30-34	35-39	40-44	45-49	50-54	55-59	60-64	65-69	70-74
	Diseases of the respiratory system	0·01	0·00	0·00	0·00	0·01	0·01	0·01	0·02	0·03	0·04	0·05	0·06	0·07	0·08	0·07	0·07
	Endocrine and metabolic diseases	0·00	0·00	0·00	0·00	0·00	0·00	0·00	0·01	0·01	0·01	0·01	0·02	0·02	0·02	0·03	0·03
	Infectious diseases	0·00	0·00	0·00	0·00	0·00	0·00	0·02	0·04	0·05	0·05	0·04	0·04	0·03	0·02	0·02	0·01
	Injuries	0·01	0·03	0·03	0·04	0·15	0·22	0·25	0·24	0·26	0·27	0·27	0·25	0·21	0·15	0·10	0·06
	Nonavoidable deaths	0·00	0·00	0·00	0·00	0·00	0·00	0·00	0·00	0·00	0·00	0·00	0·00	0·00	0·00	0·00	0·00
	Pregnancy, childbirth and perinatal period	0·22	0·00	0·00	0·00	0·00	0·00	0·00	0·00	0·00	0·00	0·00	0·00	0·00	0·00	0·00	0·00
Lithuania	Adverse effects of medical and surgical care	0·00	0·00	0·00	0·00	0·00	0·00	0·00	0·00	0·00	0·00	0·00	0·00	0·00	0·00	0·00	0·00
	Alcohol- related and drug related deaths	0·00	0·00	0·00	0·00	0·01	0·03	0·07	0·12	0·16	0·19	0·20	0·19	0·15	0·12	0·07	0·03
	Cancer	0·00	0·00	0·00	0·00	0·00	0·01	0·01	0·02	0·03	0·05	0·10	0·18	0·29	0·38	0·38	0·32
	Cardiovascular diseases	0·00	0·00	0·00	0·00	0·00	0·01	0·01	0·03	0·07	0·13	0·22	0·33	0·48	0·62	0·67	0·64
	Cerebrovascular diseases	0·00	0·00	0·00	0·00	0·00	0·00	0·01	0·01	0·02	0·03	0·05	0·07	0·11	0·15	0·19	0·20
	Congenital malformations	0·05	0·00	0·00	0·00	0·00	0·00	0·00	0·00	0·00	0·00	0·00	0·00	0·00	0·00	0·00	0·00
	Diseases of the digestive system	0·00	0·00	0·00	0·00	0·00	0·00	0·01	0·01	0·02	0·02	0·03	0·03	0·03	0·03	0·03	0·02
	Diseases of the genitourinary system	0·00	0·00	0·00	0·00	0·00	0·00	0·00	0·00	0·00	0·00	0·00	0·00	0·01	0·00	0·01	0·01
	Diseases of the nervous system	0·00	0·00	0·00	0·00	0·00	0·00	0·01	0·01	0·01	0·01	0·01	0·01	0·01	0·00	0·00	0·00
	Diseases of the respiratory system	0·01	0·00	0·00	0·00	0·00	0·01	0·01	0·02	0·03	0·04	0·05	0·06	0·07	0·09	0·09	0·09
	Endocrine and metabolic diseases	0·00	0·00	0·00	0·00	0·00	0·00	0·00	0·00	0·01	0·01	0·01	0·01	0·01	0·01	0·02	0·02
	Infectious diseases	0·01	0·01	0·00	0·00	0·00	0·00	0·01	0·03	0·04	0·05	0·05	0·05	0·04	0·04	0·03	0·02
	Injuries	0·02	0·04	0·04	0·05	0·20	0·30	0·30	0·31	0·33	0·34	0·34	0·33	0·28	0·22	0·14	0·08
	Nonavoidable deaths	0·00	0·00	0·00	0·00	0·00	0·00	0·00	0·00	0·00	0·00	0·00	0·00	0·00	0·00	0·00	0·00
	Pregnancy, childbirth and perinatal period	0·15	0·00	0·00	0·00	0·00	0·00	0·00	0·00	0·00	0·00	0·00	0·00	0·00	0·00	0·00	0·00
Luxembourg	Adverse effects of medical and surgical care	0·00	0·00	0·00	0·00	0·00	0·00	0·00	0·00	0·00	0·00	0·00	0·00	0·01	0·00	0·00	0·01

Country	Cause Group	0-1	1-4	5-9	10-14	15-19	20-24	25-29	30-34	35-39	40-44	45-49	50-54	55-59	60-64	65-69	70-74
	Alcohol- related and drug related deaths	0·00	0·00	0·00	0·00	0·01	0·02	0·02	0·02	0·04	0·04	0·06	0·08	0·07	0·08	0·06	0·04
	Cancer	0·00	0·00	0·00	0·00	0·00	0·00	0·01	0·01	0·01	0·03	0·07	0·13	0·19	0·27	0·32	0·36
	Cardiovascular diseases	0·00	0·00	0·00	0·00	0·00	0·00	0·00	0·00	0·02	0·03	0·05	0·08	0·12	0·14	0·18	0·21
	Cerebrovascular diseases	0·00	0·00	0·00	0·00	0·00	0·00	0·00	0·00	0·00	0·01	0·01	0·01	0·02	0·03	0·06	0·08
	Congenital malformations	0·01	0·00	0·00	0·00	0·00	0·00	0·00	0·00	0·00	0·00	0·00	0·00	0·00	0·00	0·00	0·00
	Diseases of the digestive system	0·00	0·00	0·00	0·00	0·00	0·00	0·00	0·00	0·00	0·00	0·00	0·00	0·00	0·01	0·00	0·01
	Diseases of the genitourinary system	0·00	0·00	0·00	0·00	0·00	0·00	0·00	0·00	0·00	0·00	0·00	0·00	0·00	0·01	0·01	0·02
	Diseases of the nervous system	0·00	0·00	0·00	0·00	0·00	0·00	0·00	0·00	0·00	0·00	0·00	0·01	0·01	0·00	0·01	0·00
	Diseases of the respiratory system	0·00	0·00	0·00	0·00	0·00	0·00	0·00	0·00	0·00	0·00	0·01	0·02	0·03	0·05	0·08	0·11
	Endocrine and metabolic diseases	0·00	0·00	0·00	0·00	0·00	0·00	0·00	0·00	0·00	0·00	0·00	0·01	0·01	0·02	0·02	0·02
	Infectious diseases	0·00	0·00	0·00	0·00	0·00	0·00	0·00	0·01	0·00	0·00	0·01	0·01	0·02	0·01	0·02	0·02
	Injuries	0·01	0·03	0·01	0·01	0·06	0·10	0·07	0·08	0·08	0·07	0·06	0·07	0·05	0·05	0·05	0·05
	Nonavoidable deaths	0·00	0·00	0·00	0·00	0·00	0·00	0·00	0·00	0·00	0·00	0·00	0·00	0·00	0·00	0·00	0·00
	Pregnancy, childbirth and perinatal period	0·13	0·00	0·00	0·00	0·00	0·00	0·00	0·00	0·00	0·00	0·00	0·00	0·00	0·00	0·00	0·00
Malta	Adverse effects of medical and surgical care	0·00	0·00	0·00	0·00	0·00	0·00	0·00	0·00	0·00	0·00	0·00	0·00	0·00	0·00	0·00	0·00
	Alcohol- related and drug related deaths	0·00	0·00	0·00	0·00	0·00	0·01	0·02	0·03	0·02	0·02	0·03	0·02	0·02	0·02	0·02	0·03
	Cancer	0·00	0·00	0·00	0·00	0·00	0·01	0·01	0·01	0·02	0·03	0·05	0·11	0·18	0·26	0·31	0·35
	Cardiovascular diseases	0·00	0·00	0·00	0·00	0·00	0·00	0·01	0·02	0·02	0·04	0·08	0·12	0·18	0·23	0·32	0·37
	Cerebrovascular diseases	0·01	0·00	0·00	0·00	0·00	0·01	0·00	0·00	0·01	0·01	0·01	0·02	0·03	0·04	0·06	0·09
	Congenital malformations	0·07	0·00	0·00	0·00	0·00	0·00	0·00	0·00	0·00	0·00	0·00	0·00	0·00	0·00	0·00	0·00
	Diseases of the digestive system	0·00	0·00	0·00	0·00	0·00	0·00	0·00	0·00	0·00	0·00	0·00	0·00	0·00	0·01	0·01	0·01
	Diseases of the genitourinary system	0·00	0·00	0·00	0·00	0·00	0·00	0·00	0·00	0·00	0·00	0·00	0·00	0·00	0·01	0·01	0·02

Country	Cause Group	0-1	1-4	5-9	10-14	15-19	20-24	25-29	30-34	35-39	40-44	45-49	50-54	55-59	60-64	65-69	70-74
	Diseases of the nervous system	0·00	0·00	0·00	0·00	0·00	0·00	0·00	0·00	0·00	0·00	0·00	0·00	0·00	0·00	0·00	0·00
	Diseases of the respiratory system	0·01	0·01	0·00	0·00	0·01	0·00	0·00	0·01	0·01	0·01	0·01	0·02	0·02	0·04	0·07	0·11
	Endocrine and metabolic diseases	0·00	0·00	0·00	0·00	0·00	0·00	0·00	0·00	0·00	0·01	0·01	0·02	0·03	0·04	0·05	0·05
	Infectious diseases	0·00	0·01	0·00	0·00	0·00	0·00	0·00	0·00	0·01	0·00	0·01	0·01	0·00	0·01	0·01	0·00
	Injuries	0·00	0·01	0·00	0·02	0·06	0·07	0·08	0·08	0·07	0·08	0·05	0·05	0·04	0·03	0·02	0·03
	Nonavoidable deaths	0·00	0·00	0·00	0·00	0·00	0·00	0·00	0·00	0·00	0·00	0·00	0·00	0·00	0·00	0·00	0·00
	Pregnancy, childbirth and perinatal period	0·25	0·00	0·00	0·00	0·00	0·00	0·00	0·00	0·00	0·00	0·00	0·00	0·00	0·00	0·00	0·00
Netherlands	Adverse effects of medical and surgical care	0·00	0·00	0·00	0·00	0·00	0·00	0·00	0·00	0·00	0·00	0·00	0·00	0·00	0·00	0·00	0·00
	Alcohol- related and drug related deaths	0·00	0·00	0·00	0·00	0·00	0·01	0·01	0·01	0·02	0·02	0·03	0·03	0·03	0·03	0·02	0·02
	Cancer	0·00	0·00	0·00	0·00	0·00	0·00	0·01	0·01	0·02	0·04	0·07	0·13	0·21	0·29	0·36	0·38
	Cardiovascular diseases	0·00	0·00	0·00	0·00	0·00	0·00	0·00	0·01	0·01	0·02	0·04	0·06	0·08	0·11	0·14	0·17
	Cerebrovascular diseases	0·00	0·00	0·00	0·00	0·00	0·00	0·00	0·00	0·00	0·01	0·01	0·02	0·02	0·03	0·05	0·07
	Congenital malformations	0·03	0·00	0·00	0·00	0·00	0·00	0·00	0·00	0·00	0·00	0·00	0·00	0·00	0·00	0·00	0·00
	Diseases of the digestive system	0·00	0·00	0·00	0·00	0·00	0·00	0·00	0·00	0·00	0·00	0·00	0·00	0·00	0·01	0·01	0·01
	Diseases of the genitourinary system	0·00	0·00	0·00	0·00	0·00	0·00	0·00	0·00	0·00	0·00	0·00	0·00	0·00	0·00	0·01	0·01
	Diseases of the nervous system	0·00	0·00	0·00	0·00	0·00	0·00	0·00	0·00	0·00	0·00	0·00	0·00	0·00	0·00	0·00	0·00
	Diseases of the respiratory system	0·00	0·00	0·00	0·00	0·00	0·00	0·00	0·00	0·00	0·00	0·01	0·01	0·03	0·04	0·07	0·11
	Endocrine and metabolic diseases	0·00	0·00	0·00	0·00	0·00	0·00	0·00	0·00	0·00	0·00	0·01	0·01	0·02	0·02	0·03	0·03
	Infectious diseases	0·00	0·00	0·00	0·00	0·00	0·00	0·00	0·00	0·00	0·00	0·01	0·01	0·01	0·01	0·01	0·02
	Injuries	0·01	0·01	0·01	0·01	0·05	0·07	0·06	0·05	0·05	0·05	0·05	0·05	0·04	0·03	0·03	0·03
	Nonavoidable deaths	0·00	0·00	0·00	0·00	0·00	0·00	0·00	0·00	0·00	0·00	0·00	0·00	0·00	0·00	0·00	0·00
	Pregnancy, childbirth and perinatal period	0·19	0·00	0·00	0·00	0·00	0·00	0·00	0·00	0·00	0·00	0·00	0·00	0·00	0·00	0·00	0·00

Country	Cause Group	0-1	1-4	5-9	10-14	15-19	20-24	25-29	30-34	35-39	40-44	45-49	50-54	55-59	60-64	65-69	70-74
Poland	Adverse effects of medical and surgical care	0·00	0·00	0·00	0·00	0·00	0·00	0·00	0·00	0·00	0·00	0·00	0·00	0·00	0·00	0·00	0·00
	Alcohol- related and drug related deaths	0·00	0·00	0·00	0·00	0·00	0·01	0·02	0·04	0·07	0·09	0·12	0·13	0·11	0·09	0·06	0·03
	Cancer	0·00	0·00	0·00	0·00	0·00	0·01	0·01	0·01	0·02	0·04	0·10	0·19	0·30	0·40	0·44	0·40
	Cardiovascular diseases	0·00	0·00	0·00	0·00	0·00	0·00	0·00	0·01	0·02	0·05	0·10	0·16	0·22	0·28	0·33	0·35
	Cerebrovascular diseases	0·00	0·00	0·00	0·00	0·00	0·00	0·00	0·01	0·01	0·02	0·04	0·06	0·08	0·10	0·13	0·14
	Congenital malformations	0·06	0·01	0·00	0·00	0·00	0·00	0·00	0·00	0·00	0·00	0·00	0·00	0·00	0·00	0·00	0·00
	Diseases of the digestive system	0·00	0·00	0·00	0·00	0·00	0·00	0·01	0·01	0·01	0·01	0·02	0·02	0·02	0·02	0·02	0·02
	Diseases of the genitourinary system	0·00	0·00	0·00	0·00	0·00	0·00	0·00	0·00	0·00	0·00	0·00	0·01	0·01	0·01	0·01	0·01
	Diseases of the nervous system	0·00	0·00	0·00	0·00	0·00	0·00	0·00	0·00	0·01	0·01	0·01	0·01	0·01	0·00	0·00	0·00
	Diseases of the respiratory system	0·01	0·01	0·00	0·00	0·01	0·01	0·01	0·01	0·01	0·02	0·03	0·04	0·05	0·07	0·09	0·11
	Endocrine and metabolic diseases	0·00	0·00	0·00	0·00	0·00	0·00	0·00	0·00	0·00	0·01	0·01	0·02	0·02	0·03	0·03	0·03
	Infectious diseases	0·01	0·00	0·00	0·00	0·00	0·00	0·00	0·01	0·01	0·01	0·01	0·01	0·01	0·01	0·01	0·01
	Injuries	0·01	0·01	0·01	0·02	0·13	0·19	0·15	0·14	0·14	0·15	0·15	0·15	0·12	0·09	0·06	0·05
	Nonavoidable deaths	0·00	0·00	0·00	0·00	0·00	0·00	0·00	0·00	0·00	0·00	0·00	0·00	0·00	0·00	0·00	0·00
	Pregnancy, childbirth and perinatal period	0·22	0·00	0·00	0·00	0·00	0·00	0·00	0·00	0·00	0·00	0·00	0·00	0·00	0·00	0·00	0·00
Portugal	Adverse effects of medical and surgical care	0·00	0·00	0·00	0·00	0·00	0·00	0·00	0·00	0·00	0·00	0·00	0·00	0·00	0·00	0·00	0·00
	Alcohol- related and drug related deaths	0·00	0·00	0·00	0·00	0·00	0·00	0·01	0·01	0·02	0·04	0·05	0·06	0·05	0·05	0·04	0·03
	Cancer	0·00	0·00	0·00	0·00	0·00	0·01	0·01	0·02	0·03	0·07	0·13	0·22	0·29	0·33	0·34	0·32
	Cardiovascular diseases	0·00	0·00	0·00	0·00	0·00	0·00	0·01	0·01	0·02	0·03	0·05	0·08	0·10	0·12	0·13	0·16
	Cerebrovascular diseases	0·00	0·00	0·00	0·00	0·00	0·00	0·00	0·00	0·01	0·02	0·02	0·04	0·05	0·07	0·09	0·14
	Congenital malformations	0·03	0·00	0·00	0·00	0·00	0·00	0·00	0·00	0·00	0·00	0·00	0·00	0·00	0·00	0·00	0·00
	Diseases of the digestive system	0·00	0·00	0·00	0·00	0·00	0·00	0·00	0·00	0·00	0·00	0·01	0·01	0·01	0·01	0·01	0·01

Country	Cause Group	0-1	1-4	5-9	10-14	15-19	20-24	25-29	30-34	35-39	40-44	45-49	50-54	55-59	60-64	65-69	70-74
	Diseases of the genitourinary system	0·00	0·00	0·00	0·00	0·00	0·00	0·00	0·00	0·00	0·00	0·00	0·00	0·00	0·01	0·01	0·01
	Diseases of the nervous system	0·00	0·00	0·00	0·00	0·00	0·00	0·00	0·00	0·00	0·00	0·00	0·00	0·00	0·00	0·00	0·00
	Diseases of the respiratory system	0·00	0·00	0·00	0·00	0·00	0·00	0·00	0·01	0·01	0·01	0·02	0·03	0·04	0·05	0·07	0·10
	Endocrine and metabolic diseases	0·00	0·00	0·00	0·00	0·00	0·00	0·00	0·00	0·00	0·00	0·01	0·01	0·02	0·03	0·04	0·06
	Infectious diseases	0·00	0·00	0·00	0·00	0·00	0·00	0·01	0·02	0·04	0·04	0·04	0·04	0·03	0·02	0·02	0·02
	Injuries	0·00	0·02	0·01	0·01	0·06	0·09	0·09	0·08	0·08	0·08	0·08	0·09	0·07	0·06	0·05	0·05
	Nonavoidable deaths	0·00	0·00	0·00	0·00	0·00	0·00	0·00	0·00	0·00	0·00	0·00	0·00	0·00	0·00	0·00	0·00
	Pregnancy, childbirth and perinatal period	0·16	0·00	0·00	0·00	0·00	0·00	0·00	0·00	0·00	0·00	0·00	0·00	0·00	0·00	0·00	0·00
Romania	Adverse effects of medical and surgical care	0·00	0·00	0·00	0·00	0·00	0·00	0·00	0·00	0·00	0·00	0·00	0·00	0·00	0·00	0·00	0·00
	Alcohol- related and drug related deaths	0·00	0·00	0·00	0·00	0·00	0·01	0·01	0·02	0·04	0·08	0·12	0·15	0·16	0·14	0·11	0·07
	Cancer	0·00	0·00	0·00	0·00	0·01	0·01	0·01	0·02	0·03	0·07	0·15	0·26	0·37	0·40	0·38	0·30
	Cardiovascular diseases	0·00	0·00	0·00	0·00	0·00	0·01	0·01	0·02	0·05	0·10	0·17	0·27	0·37	0·45	0·51	0·56
	Cerebrovascular diseases	0·00	0·00	0·00	0·00	0·00	0·00	0·00	0·01	0·02	0·03	0·06	0·10	0·15	0·21	0·27	0·32
	Congenital malformations	0·10	0·01	0·00	0·00	0·00	0·00	0·00	0·00	0·00	0·00	0·00	0·00	0·00	0·00	0·00	0·00
	Diseases of the digestive system	0·00	0·00	0·00	0·00	0·00	0·00	0·00	0·01	0·01	0·01	0·02	0·02	0·02	0·02	0·01	0·01
	Diseases of the genitourinary system	0·00	0·00	0·00	0·00	0·00	0·00	0·00	0·00	0·00	0·00	0·01	0·01	0·01	0·02	0·02	0·02
	Diseases of the nervous system	0·00	0·00	0·00	0·00	0·00	0·00	0·00	0·00	0·00	0·00	0·00	0·00	0·00	0·00	0·00	0·00
	Diseases of the respiratory system	0·20	0·03	0·01	0·01	0·01	0·01	0·01	0·02	0·03	0·04	0·06	0·07	0·09	0·10	0·11	0·11
	Endocrine and metabolic diseases	0·00	0·00	0·00	0·00	0·00	0·00	0·00	0·00	0·00	0·00	0·01	0·01	0·01	0·02	0·02	0·02
	Infectious diseases	0·02	0·01	0·00	0·00	0·01	0·01	0·01	0·02	0·02	0·03	0·04	0·04	0·03	0·03	0·02	0·02
	Injuries	0·03	0·04	0·04	0·05	0·11	0·14	0·12	0·12	0·13	0·15	0·16	0·15	0·13	0·10	0·07	0·05
	Nonavoidable deaths	0·00	0·00	0·00	0·00	0·00	0·00	0·00	0·00	0·00	0·00	0·00	0·00	0·00	0·00	0·00	0·00

Country	Cause Group	0-1	1-4	5-9	10-14	15-19	20-24	25-29	30-34	35-39	40-44	45-49	50-54	55-59	60-64	65-69	70-74
	Pregnancy, childbirth and perinatal period	0·30	0·00	0·00	0·00	0·00	0·00	0·00	0·00	0·00	0·00	0·00	0·00	0·00	0·00	0·00	0·00
Slovakia	Adverse effects of medical and surgical care	0·00	0·00	0·00	0·00	0·00	0·00	0·00	0·00	0·00	0·00	0·00	0·00	0·00	0·00	0·00	0·00
	Alcohol- related and drug related deaths	0·00	0·00	0·00	0·00	0·00	0·01	0·02	0·03	0·06	0·09	0·12	0·15	0·14	0·14	0·09	0·05
	Cancer	0·00	0·00	0·00	0·00	0·00	0·01	0·01	0·01	0·02	0·04	0·11	0·21	0·31	0·39	0·42	0·37
	Cardiovascular diseases	0·00	0·00	0·00	0·00	0·00	0·00	0·01	0·01	0·03	0·07	0·14	0·22	0·33	0·45	0·52	0·57
	Cerebrovascular diseases	0·00	0·00	0·00	0·00	0·00	0·00	0·00	0·00	0·01	0·02	0·03	0·06	0·08	0·12	0·16	0·18
	Congenital malformations	0·06	0·01	0·00	0·00	0·00	0·00	0·00	0·00	0·00	0·00	0·00	0·00	0·00	0·00	0·00	0·00
	Diseases of the digestive system	0·00	0·00	0·00	0·00	0·00	0·00	0·00	0·00	0·01	0·01	0·01	0·02	0·02	0·02	0·02	0·02
	Diseases of the genitourinary system	0·00	0·00	0·00	0·00	0·00	0·00	0·00	0·00	0·00	0·00	0·00	0·01	0·01	0·01	0·02	0·02
	Diseases of the nervous system	0·00	0·00	0·00	0·00	0·00	0·00	0·00	0·00	0·01	0·01	0·01	0·01	0·01	0·01	0·00	0·00
	Diseases of the respiratory system	0·03	0·01	0·00	0·00	0·01	0·01	0·01	0·01	0·01	0·02	0·03	0·04	0·06	0·08	0·09	0·11
	Endocrine and metabolic diseases	0·00	0·00	0·00	0·00	0·00	0·00	0·00	0·00	0·00	0·01	0·01	0·01	0·02	0·02	0·03	0·03
	Infectious diseases	0·01	0·00	0·00	0·00	0·00	0·00	0·00	0·00	0·00	0·00	0·01	0·01	0·01	0·01	0·01	0·01
	Injuries	0·01	0·02	0·02	0·02	0·09	0·14	0·11	0·11	0·12	0·14	0·15	0·16	0·14	0·11	0·08	0·06
	Nonavoidable deaths	0·00	0·00	0·00	0·00	0·00	0·00	0·00	0·00	0·00	0·00	0·00	0·00	0·00	0·00	0·00	0·00
	Pregnancy, childbirth and perinatal period	0·21	0·00	0·00	0·00	0·00	0·00	0·00	0·00	0·00	0·00	0·00	0·00	0·00	0·00	0·00	0·00
Slovenia	Adverse effects of medical and surgical care	0·00	0·00	0·00	0·00	0·00	0·00	0·00	0·00	0·00	0·00	0·00	0·01	0·01	0·01	0·01	0·01
	Alcohol- related and drug related deaths	0·00	0·00	0·00	0·00	0·01	0·02	0·03	0·03	0·05	0·07	0·11	0·15	0·16	0·17	0·15	0·12
	Cancer	0·00	0·00	0·00	0·00	0·00	0·01	0·01	0·01	0·02	0·04	0·09	0·18	0·30	0·38	0·42	0·41
	Cardiovascular diseases	0·00	0·00	0·00	0·00	0·00	0·00	0·00	0·01	0·02	0·03	0·07	0·11	0·15	0·20	0·22	0·26
	Cerebrovascular diseases	0·00	0·00	0·00	0·00	0·00	0·00	0·00	0·00	0·01	0·01	0·02	0·03	0·04	0·06	0·10	0·13
	Congenital malformations	0·02	0·00	0·00	0·00	0·00	0·00	0·00	0·00	0·00	0·00	0·00	0·00	0·00	0·00	0·00	0·00

Country	Cause Group	0-1	1-4	5-9	10-14	15-19	20-24	25-29	30-34	35-39	40-44	45-49	50-54	55-59	60-64	65-69	70-74
	Diseases of the digestive system	0·00	0·00	0·00	0·00	0·00	0·00	0·00	0·00	0·00	0·00	0·01	0·01	0·01	0·01	0·01	0·01
	Diseases of the genitourinary system	0·00	0·00	0·00	0·00	0·00	0·00	0·00	0·00	0·00	0·00	0·00	0·00	0·00	0·00	0·01	0·01
	Diseases of the nervous system	0·00	0·00	0·00	0·00	0·00	0·00	0·00	0·00	0·00	0·00	0·00	0·00	0·00	0·00	0·00	0·00
	Diseases of the respiratory system	0·00	0·00	0·00	0·00	0·00	0·00	0·00	0·00	0·00	0·00	0·01	0·01	0·02	0·03	0·05	0·08
	Endocrine and metabolic diseases	0·00	0·00	0·00	0·00	0·00	0·00	0·00	0·00	0·00	0·00	0·00	0·01	0·01	0·02	0·02	0·03
	Infectious diseases	0·00	0·00	0·00	0·00	0·00	0·00	0·00	0·00	0·00	0·00	0·00	0·00	0·00	0·00	0·01	0·01
	Injuries	0·00	0·01	0·01	0·02	0·10	0·15	0·13	0·11	0·10	0·12	0·12	0·12	0·11	0·10	0·08	0·08
	Nonavoidable deaths	0·00	0·00	0·00	0·00	0·00	0·00	0·00	0·00	0·00	0·00	0·00	0·00	0·00	0·00	0·00	0·00
	Pregnancy, childbirth and perinatal period	0·12	0·00	0·00	0·00	0·00	0·00	0·00	0·00	0·00	0·00	0·00	0·00	0·00	0·00	0·00	0·00
Spain	Adverse effects of medical and surgical care	0·00	0·00	0·00	0·00	0·00	0·00	0·00	0·00	0·00	0·00	0·00	0·00	0·00	0·00	0·00	0·00
	Alcohol- related and drug related deaths	0·00	0·00	0·00	0·00	0·00	0·00	0·01	0·01	0·02	0·03	0·04	0·04	0·05	0·04	0·04	0·03
	Cancer	0·00	0·00	0·00	0·00	0·00	0·00	0·01	0·01	0·02	0·04	0·10	0·19	0·29	0·37	0·40	0·40
	Cardiovascular diseases	0·00	0·00	0·00	0·00	0·00	0·00	0·00	0·01	0·01	0·03	0·05	0·08	0·11	0·14	0·16	0·19
	Cerebrovascular diseases	0·00	0·00	0·00	0·00	0·00	0·00	0·00	0·00	0·00	0·01	0·02	0·02	0·03	0·04	0·05	0·08
	Congenital malformations	0·03	0·00	0·00	0·00	0·00	0·00	0·00	0·00	0·00	0·00	0·00	0·00	0·00	0·00	0·00	0·00
	Diseases of the digestive system	0·00	0·00	0·00	0·00	0·00	0·00	0·00	0·00	0·00	0·00	0·00	0·00	0·01	0·01	0·01	0·01
	Diseases of the genitourinary system	0·00	0·00	0·00	0·00	0·00	0·00	0·00	0·00	0·00	0·00	0·00	0·00	0·00	0·01	0·01	0·02
	Diseases of the nervous system	0·00	0·00	0·00	0·00	0·00	0·00	0·00	0·00	0·00	0·00	0·00	0·00	0·00	0·00	0·00	0·00
	Diseases of the respiratory system	0·00	0·00	0·00	0·00	0·00	0·00	0·00	0·01	0·01	0·01	0·02	0·02	0·03	0·05	0·07	0·11
	Endocrine and metabolic diseases	0·00	0·00	0·00	0·00	0·00	0·00	0·00	0·00	0·00	0·00	0·00	0·01	0·01	0·02	0·02	0·03
	Infectious diseases	0·00	0·00	0·00	0·00	0·00	0·00	0·00	0·01	0·01	0·02	0·03	0·02	0·02	0·01	0·01	0·02
	Injuries	0·00	0·01	0·01	0·01	0·06	0·08	0·07	0·07	0·06	0·06	0·06	0·06	0·05	0·04	0·04	0·04

Country	Cause Group	0-1	1-4	5-9	10-14	15-19	20-24	25-29	30-34	35-39	40-44	45-49	50-54	55-59	60-64	65-69	70-74
	Nonavoidable deaths	0·00	0·00	0·00	0·00	0·00	0·00	0·00	0·00	0·00	0·00	0·00	0·00	0·00	0·00	0·00	0·00
	Pregnancy, childbirth and perinatal period	0·15	0·00	0·00	0·00	0·00	0·00	0·00	0·00	0·00	0·00	0·00	0·00	0·00	0·00	0·00	0·00
Sweden	Adverse effects of medical and surgical care	0·00	0·00	0·00	0·00	0·00	0·00	0·00	0·00	0·00	0·00	0·00	0·00	0·00	0·00	0·00	0·00
	Alcohol- related and drug related deaths	0·00	0·00	0·00	0·00	0·01	0·05	0·06	0·05	0·04	0·04	0·05	0·06	0·06	0·06	0·05	0·03
	Cancer	0·00	0·00	0·00	0·00	0·00	0·00	0·01	0·01	0·02	0·02	0·04	0·08	0·12	0·19	0·24	0·26
	Cardiovascular diseases	0·00	0·00	0·00	0·00	0·00	0·00	0·00	0·00	0·01	0·02	0·04	0·08	0·13	0·19	0·24	0·28
	Cerebrovascular diseases	0·00	0·00	0·00	0·00	0·00	0·00	0·00	0·00	0·00	0·01	0·01	0·02	0·02	0·04	0·05	0·08
	Congenital malformations	0·02	0·00	0·00	0·00	0·00	0·00	0·00	0·00	0·00	0·00	0·00	0·00	0·00	0·00	0·00	0·00
	Diseases of the digestive system	0·00	0·00	0·00	0·00	0·00	0·00	0·00	0·00	0·00	0·00	0·00	0·00	0·01	0·01	0·01	0·01
	Diseases of the genitourinary system	0·00	0·00	0·00	0·00	0·00	0·00	0·00	0·00	0·00	0·00	0·00	0·00	0·00	0·00	0·01	0·01
	Diseases of the nervous system	0·00	0·00	0·00	0·00	0·00	0·00	0·00	0·00	0·00	0·00	0·00	0·00	0·00	0·00	0·00	0·00
	Diseases of the respiratory system	0·00	0·00	0·00	0·00	0·00	0·00	0·00	0·00	0·00	0·00	0·01	0·01	0·02	0·03	0·05	0·06
	Endocrine and metabolic diseases	0·00	0·00	0·00	0·00	0·00	0·00	0·00	0·00	0·00	0·00	0·01	0·01	0·01	0·02	0·03	0·03
	Infectious diseases	0·00	0·00	0·00	0·00	0·00	0·00	0·00	0·00	0·00	0·00	0·01	0·01	0·01	0·01	0·01	0·02
	Injuries	0·00	0·01	0·01	0·01	0·06	0·10	0·08	0·06	0·05	0·06	0·06	0·06	0·06	0·05	0·05	0·04
	Nonavoidable deaths	0·00	0·00	0·00	0·00	0·00	0·00	0·00	0·00	0·00	0·00	0·00	0·00	0·00	0·00	0·00	0·00
	Pregnancy, childbirth and perinatal period	0·10	0·00	0·00	0·00	0·00	0·00	0·00	0·00	0·00	0·00	0·00	0·00	0·00	0·00	0·00	0·00
United Kingdom	Adverse effects of medical and surgical care	0·00	0·00	0·00	0·00	0·00	0·00	0·00	0·00	0·00	0·00	0·00	0·00	0·00	0·00	0·00	0·00
	Alcohol- related and drug related deaths	0·00	0·00	0·00	0·00	0·01	0·03	0·04	0·06	0·08	0·09	0·08	0·08	0·07	0·05	0·04	0·02
	Cancer	0·00	0·00	0·00	0·00	0·00	0·00	0·01	0·01	0·02	0·03	0·06	0·11	0·19	0·27	0·33	0·35
	Cardiovascular diseases	0·00	0·00	0·00	0·00	0·00	0·00	0·01	0·01	0·03	0·05	0·09	0·13	0·17	0·22	0·27	0·31
	Cerebrovascular diseases	0·00	0·00	0·00	0·00	0·00	0·00	0·00	0·00	0·01	0·01	0·02	0·02	0·03	0·04	0·05	0·08

Country	Cause Group	0-1	1-4	5-9	10-14	15-19	20-24	25-29	30-34	35-39	40-44	45-49	50-54	55-59	60-64	65-69	70-74
	Congenital malformations	0·03	0·00	0·00	0·00	0·00	0·00	0·00	0·00	0·00	0·00	0·00	0·00	0·00	0·00	0·00	0·00
	Diseases of the digestive system	0·00	0·00	0·00	0·00	0·00	0·00	0·00	0·00	0·00	0·00	0·01	0·01	0·01	0·01	0·01	0·02
	Diseases of the genitourinary system	0·00	0·00	0·00	0·00	0·00	0·00	0·00	0·00	0·00	0·00	0·00	0·00	0·00	0·00	0·00	0·01
	Diseases of the nervous system	0·00	0·00	0·00	0·00	0·00	0·00	0·00	0·00	0·00	0·00	0·00	0·00	0·00	0·00	0·00	0·00
	Diseases of the respiratory system	0·01	0·01	0·00	0·00	0·00	0·00	0·00	0·01	0·01	0·01	0·02	0·03	0·05	0·08	0·11	0·15
	Endocrine and metabolic diseases	0·00	0·00	0·00	0·00	0·00	0·00	0·00	0·00	0·00	0·00	0·01	0·01	0·01	0·01	0·01	0·02
	Infectious diseases	0·01	0·00	0·00	0·00	0·00	0·00	0·00	0·00	0·00	0·01	0·01	0·01	0·01	0·01	0·01	0·01
	Injuries	0·01	0·01	0·01	0·01	0·07	0·09	0·08	0·07	0·07	0·07	0·06	0·05	0·04	0·03	0·02	0·02
	Nonavoidable deaths	0·00	0·00	0·00	0·00	0·00	0·00	0·00	0·00	0·00	0·00	0·00	0·00	0·00	0·00	0·00	0·00
	Pregnancy, childbirth and perinatal period	0·22	0·00	0·00	0·00	0·00	0·00	0·00	0·00	0·00	0·00	0·00	0·00	0·00	0·00	0·00	0·00
Established member states	Adverse effects of medical and surgical care	0·00	0·00	0·00	0·00	0·00	0·00	0·00	0·00	0·00	0·00	0·00	0·00	0·00	0·00	0·00	0·00
	Alcohol- related and drug related deaths	0·00	0·00	0·00	0·00	0·01	0·02	0·03	0·03	0·04	0·05	0·07	0·08	0·08	0·07	0·06	0·04
	Cancer	0·00	0·00	0·00	0·00	0·00	0·00	0·01	0·01	0·02	0·04	0·07	0·14	0·21	0·29	0·34	0·34
	Cardiovascular diseases	0·00	0·00	0·00	0·00	0·00	0·00	0·00	0·01	0·02	0·03	0·06	0·09	0·13	0·17	0·21	0·24
	Cerebrovascular diseases	0·00	0·00	0·00	0·00	0·00	0·00	0·00	0·00	0·01	0·01	0·01	0·02	0·03	0·04	0·06	0·08
	Congenital malformations	0·03	0·00	0·00	0·00	0·00	0·00	0·00	0·00	0·00	0·00	0·00	0·00	0·00	0·00	0·00	0·00
	Diseases of the digestive system	0·00	0·00	0·00	0·00	0·00	0·00	0·00	0·00	0·00	0·00	0·00	0·01	0·01	0·01	0·01	0·01
	Diseases of the genitourinary system	0·00	0·00	0·00	0·00	0·00	0·00	0·00	0·00	0·00	0·00	0·00	0·00	0·00	0·01	0·01	0·01
	Diseases of the nervous system	0·00	0·00	0·00	0·00	0·00	0·00	0·00	0·00	0·00	0·00	0·00	0·00	0·00	0·00	0·00	0·00
	Diseases of the respiratory system	0·00	0·00	0·00	0·00	0·00	0·00	0·00	0·00	0·00	0·01	0·01	0·02	0·03	0·05	0·07	0·10
	Endocrine and metabolic diseases	0·00	0·00	0·00	0·00	0·00	0·00	0·00	0·00	0·00	0·00	0·01	0·01	0·02	0·02	0·03	0·03
	Infectious diseases	0·00	0·00	0·00	0·00	0·00	0·00	0·00	0·00	0·01	0·01	0·01	0·01	0·01	0·01	0·01	0·02

Country	Cause Group	0-1	1-4	5-9	10-14	15-19	20-24	25-29	30-34	35-39	40-44	45-49	50-54	55-59	60-64	65-69	70-74
	Injuries	0·01	0·01	0·01	0·01	0·07	0·10	0·09	0·08	0·07	0·07	0·07	0·07	0·06	0·05	0·05	0·04
	Nonavoidable deaths	0·00	0·00	0·00	0·00	0·00	0·00	0·00	0·00	0·00	0·00	0·00	0·00	0·00	0·00	0·00	0·00
	Pregnancy, childbirth and perinatal period	0·15	0·00	0·00	0·00	0·00	0·00	0·00	0·00	0·00	0·00	0·00	0·00	0·00	0·00	0·00	0·00
New member states	Adverse effects of medical and surgical care	0·00	0·00	0·00	0·00	0·00	0·00	0·00	0·00	0·00	0·00	0·00	0·00	0·00	0·00	0·00	0·00
	Alcohol- related and drug related deaths	0·00	0·00	0·00	0·00	0·00	0·02	0·04	0·05	0·07	0·09	0·12	0·14	0·13	0·11	0·08	0·05
	Cancer	0·00	0·00	0·00	0·00	0·00	0·01	0·01	0·01	0·02	0·05	0·10	0·19	0·29	0·37	0·39	0·35
	Cardiovascular diseases	0·00	0·00	0·00	0·00	0·00	0·00	0·01	0·02	0·04	0·08	0·14	0·22	0·31	0·40	0·45	0·47
	Cerebrovascular diseases	0·00	0·00	0·00	0·00	0·00	0·00	0·00	0·01	0·01	0·02	0·04	0·06	0·09	0·12	0·16	0·19
	Congenital malformations	0·05	0·00	0·00	0·00	0·00	0·00	0·00	0·00	0·00	0·00	0·00	0·00	0·00	0·00	0·00	0·00
	Diseases of the digestive system	0·00	0·00	0·00	0·00	0·00	0·00	0·00	0·00	0·01	0·01	0·01	0·02	0·02	0·02	0·02	0·02
	Diseases of the genitourinary system	0·00	0·00	0·00	0·00	0·00	0·00	0·00	0·00	0·00	0·00	0·00	0·01	0·01	0·01	0·01	0·01
	Diseases of the nervous system	0·00	0·00	0·00	0·00	0·00	0·00	0·00	0·00	0·00	0·01	0·01	0·01	0·00	0·00	0·00	0·00
	Diseases of the respiratory system	0·03	0·01	0·00	0·00	0·00	0·00	0·01	0·01	0·01	0·02	0·03	0·04	0·05	0·07	0·08	0·09
	Endocrine and metabolic diseases	0·00	0·00	0·00	0·00	0·00	0·00	0·00	0·00	0·00	0·01	0·01	0·01	0·02	0·03	0·03	0·04
	Infectious diseases	0·01	0·00	0·00	0·00	0·00	0·00	0·01	0·01	0·01	0·02	0·02	0·02	0·02	0·02	0·01	0·01
	Injuries	0·01	0·02	0·02	0·03	0·11	0·16	0·15	0·14	0·14	0·15	0·15	0·15	0·13	0·10	0·07	0·06
	Nonavoidable deaths	0·00	0·00	0·00	0·00	0·00	0·00	0·00	0·00	0·00	0·00	0·00	0·00	0·00	0·00	0·00	0·00
	Pregnancy, childbirth and perinatal period	0·20	0·00	0·00	0·00	0·00	0·00	0·00	0·00	0·00	0·00	0·00	0·00	0·00	0·00	0·00	0·00

Supplementary Table 6: **The average contribution of cause groups to the estimated gains in female life expectancy by EU member state if all avoidable deaths were averted, 2005-2019**

Country	Cause Group	0-1	1-4	5-9	10-14	15-19	20-24	25-29	30-34	35-39	40-44	45-49	50-54	55-59	60-64	65-69	70-74
Austria	Adverse effects of medical and surgical care	0·00	0·00	0·00	0·00	0·00	0·00	0·00	0·00	0·00	0·00	0·00	0·00	0·00	0·00	0·00	0·00
	Alcohol- related and drug related deaths	0·00	0·00	0·00	0·00	0·01	0·01	0·01	0·01	0·01	0·02	0·02	0·03	0·03	0·03	0·03	0·02
	Cancer	0·00	0·00	0·00	0·00	0·00	0·00	0·01	0·02	0·03	0·06	0·09	0·14	0·19	0·23	0·25	0·24
	Cardiovascular diseases	0·00	0·00	0·00	0·00	0·00	0·00	0·00	0·00	0·01	0·01	0·02	0·03	0·04	0·07	0·11	0·18
	Cerebrovascular diseases	0·00	0·00	0·00	0·00	0·00	0·00	0·00	0·00	0·00	0·01	0·01	0·01	0·02	0·02	0·03	0·06
	Congenital malformations	0·03	0·00	0·00	0·00	0·00	0·00	0·00	0·00	0·00	0·00	0·00	0·00	0·00	0·00	0·00	0·00
	Diseases of the digestive system	0·00	0·00	0·00	0·00	0·00	0·00	0·00	0·00	0·00	0·00	0·00	0·00	0·00	0·00	0·01	0·01
	Diseases of the genitourinary system	0·00	0·00	0·00	0·00	0·00	0·00	0·00	0·00	0·00	0·00	0·00	0·00	0·00	0·00	0·01	0·01
	Diseases of the nervous system	0·00	0·00	0·00	0·00	0·00	0·00	0·00	0·00	0·00	0·00	0·00	0·00	0·00	0·00	0·00	0·00
	Diseases of the respiratory system	0·00	0·00	0·00	0·00	0·00	0·00	0·00	0·00	0·00	0·00	0·01	0·01	0·02	0·03	0·05	0·06
	Endocrine and metabolic diseases	0·00	0·00	0·00	0·00	0·00	0·00	0·00	0·00	0·00	0·00	0·00	0·01	0·01	0·02	0·03	0·04
	Infectious diseases	0·00	0·00	0·00	0·00	0·00	0·00	0·00	0·00	0·00	0·00	0·00	0·00	0·00	0·00	0·01	0·01
	Injuries	0·00	0·01	0·00	0·01	0·03	0·03	0·02	0·02	0·02	0·02	0·02	0·03	0·03	0·02	0·02	0·03
	Nonavoidable deaths	0·00	0·00	0·00	0·00	0·00	0·00	0·00	0·00	0·00	0·00	0·00	0·00	0·00	0·00	0·00	0·00
	Pregnancy, childbirth and perinatal period	0·14	0·00	0·00	0·00	0·00	0·00	0·00	0·00	0·00	0·00	0·00	0·00	0·00	0·00	0·00	0·00
Belgium	Adverse effects of medical and surgical care	0·00	0·00	0·00	0·00	0·00	0·00	0·00	0·00	0·00	0·00	0·00	0·00	0·00	0·00	0·00	0·00
	Alcohol- related and drug related deaths	0·00	0·00	0·00	0·00	0·00	0·00	0·01	0·01	0·02	0·02	0·03	0·04	0·04	0·03	0·03	0·02
	Cancer	0·00	0·00	0·00	0·00	0·00	0·00	0·01	0·02	0·04	0·06	0·10	0·16	0·21	0·25	0·26	0·25
	Cardiovascular diseases	0·00	0·00	0·00	0·00	0·00	0·00	0·00	0·00	0·00	0·01	0·02	0·02	0·04	0·05	0·07	0·10
	Cerebrovascular diseases	0·00	0·00	0·00	0·00	0·00	0·00	0·00	0·00	0·01	0·01	0·01	0·02	0·02	0·03	0·04	0·06

Country	Cause Group	0-1	1-4	5-9	10-14	15-19	20-24	25-29	30-34	35-39	40-44	45-49	50-54	55-59	60-64	65-69	70-74
	Congenital malformations	0·02	0·00	0·00	0·00	0·00	0·00	0·00	0·00	0·00	0·00	0·00	0·00	0·00	0·00	0·00	0·00
	Diseases of the digestive system	0·00	0·00	0·00	0·00	0·00	0·00	0·00	0·00	0·00	0·00	0·00	0·00	0·00	0·00	0·01	0·01
	Diseases of the genitourinary system	0·00	0·00	0·00	0·00	0·00	0·00	0·00	0·00	0·00	0·00	0·00	0·00	0·00	0·00	0·01	0·01
	Diseases of the nervous system	0·00	0·00	0·00	0·00	0·00	0·00	0·00	0·00	0·00	0·00	0·00	0·00	0·00	0·00	0·00	0·00
	Diseases of the respiratory system	0·00	0·00	0·00	0·00	0·00	0·00	0·00	0·00	0·00	0·01	0·01	0·02	0·03	0·05	0·07	0·08
	Endocrine and metabolic diseases	0·00	0·00	0·00	0·00	0·00	0·00	0·00	0·00	0·00	0·00	0·00	0·00	0·01	0·01	0·01	0·02
	Infectious diseases	0·00	0·00	0·00	0·00	0·00	0·00	0·00	0·00	0·00	0·00	0·00	0·01	0·01	0·01	0·01	0·02
	Injuries	0·01	0·01	0·01	0·01	0·03	0·03	0·03	0·03	0·03	0·04	0·04	0·04	0·04	0·04	0·03	0·04
	Nonavoidable deaths	0·00	0·00	0·00	0·00	0·00	0·00	0·00	0·00	0·00	0·00	0·00	0·00	0·00	0·00	0·00	0·00
	Pregnancy, childbirth and perinatal period	0·13	0·00	0·00	0·00	0·00	0·00	0·00	0·00	0·00	0·00	0·00	0·00	0·00	0·00	0·00	0·00
Bulgaria	Adverse effects of medical and surgical care	0·00	0·00	0·00	0·00	0·00	0·00	0·00	0·00	0·00	0·00	0·00	0·00	0·00	0·00	0·00	0·00
	Alcohol- related and drug related deaths	0·00	0·00	0·00	0·00	0·00	0·00	0·00	0·01	0·01	0·01	0·02	0·02	0·02	0·02	0·02	0·02
	Cancer	0·00	0·00	0·00	0·00	0·00	0·01	0·01	0·03	0·06	0·09	0·13	0·17	0·19	0·20	0·19	0·17
	Cardiovascular diseases	0·00	0·00	0·00	0·00	0·00	0·00	0·01	0·01	0·01	0·03	0·05	0·08	0·12	0·17	0·23	0·32
	Cerebrovascular diseases	0·01	0·00	0·00	0·00	0·00	0·00	0·00	0·01	0·01	0·02	0·04	0·06	0·09	0·13	0·20	0·29
	Congenital malformations	0·06	0·01	0·00	0·00	0·00	0·00	0·00	0·00	0·00	0·00	0·00	0·00	0·00	0·00	0·00	0·00
	Diseases of the digestive system	0·00	0·00	0·00	0·00	0·00	0·00	0·00	0·00	0·00	0·00	0·00	0·00	0·00	0·01	0·01	0·01
	Diseases of the genitourinary system	0·00	0·00	0·00	0·00	0·00	0·00	0·00	0·00	0·00	0·01	0·01	0·01	0·01	0·01	0·02	0·02
	Diseases of the nervous system	0·00	0·00	0·00	0·00	0·00	0·00	0·00	0·00	0·00	0·00	0·00	0·00	0·00	0·00	0·00	0·00
	Diseases of the respiratory system	0·07	0·02	0·01	0·01	0·01	0·01	0·01	0·01	0·01	0·01	0·01	0·01	0·02	0·02	0·03	0·03
	Endocrine and metabolic diseases	0·00	0·00	0·00	0·00	0·00	0·00	0·00	0·00	0·00	0·00	0·01	0·01	0·01	0·02	0·03	0·04
	Infectious diseases	0·02	0·01	0·00	0·00	0·00	0·00	0·00	0·00	0·00	0·00	0·01	0·00	0·01	0·01	0·01	0·01

Country	Cause Group	0-1	1-4	5-9	10-14	15-19	20-24	25-29	30-34	35-39	40-44	45-49	50-54	55-59	60-64	65-69	70-74
	Injuries	0·01	0·02	0·01	0·02	0·04	0·03	0·02	0·02	0·02	0·02	0·02	0·02	0·02	0·02	0·01	0·01
	Nonavoidable deaths	0·00	0·00	0·00	0·00	0·00	0·00	0·00	0·00	0·00	0·00	0·00	0·00	0·00	0·00	0·00	0·00
	Pregnancy, childbirth and perinatal period	0·24	0·00	0·00	0·00	0·00	0·00	0·00	0·00	0·00	0·00	0·00	0·00	0·00	0·00	0·00	0·00
Croatia	Adverse effects of medical and surgical care	0·00	0·00	0·00	0·00	0·00	0·00	0·00	0·00	0·00	0·00	0·00	0·00	0·00	0·00	0·00	0·00
	Alcohol- related and drug related deaths	0·00	0·00	0·00	0·00	0·00	0·00	0·00	0·00	0·01	0·01	0·02	0·03	0·03	0·03	0·03	0·02
	Cancer	0·00	0·00	0·00	0·00	0·00	0·00	0·01	0·02	0·04	0·07	0·12	0·17	0·22	0·24	0·26	0·24
	Cardiovascular diseases	0·00	0·00	0·00	0·00	0·00	0·00	0·00	0·00	0·01	0·01	0·03	0·04	0·07	0·11	0·18	0·28
	Cerebrovascular diseases	0·00	0·00	0·00	0·00	0·00	0·00	0·00	0·00	0·00	0·01	0·02	0·03	0·04	0·07	0·12	0·20
	Congenital malformations	0·03	0·00	0·00	0·00	0·00	0·00	0·00	0·00	0·00	0·00	0·00	0·00	0·00	0·00	0·00	0·00
	Diseases of the digestive system	0·00	0·00	0·00	0·00	0·00	0·00	0·00	0·00	0·00	0·00	0·00	0·00	0·01	0·01	0·01	0·01
	Diseases of the genitourinary system	0·00	0·00	0·00	0·00	0·00	0·00	0·00	0·00	0·00	0·00	0·00	0·00	0·00	0·01	0·01	0·01
	Diseases of the nervous system	0·00	0·00	0·00	0·00	0·00	0·00	0·00	0·00	0·00	0·00	0·00	0·00	0·00	0·00	0·00	0·00
	Diseases of the respiratory system	0·01	0·00	0·00	0·00	0·00	0·00	0·00	0·00	0·00	0·01	0·01	0·01	0·02	0·02	0·03	0·04
	Endocrine and metabolic diseases	0·00	0·00	0·00	0·00	0·00	0·00	0·00	0·00	0·00	0·00	0·00	0·01	0·01	0·02	0·03	0·06
	Infectious diseases	0·00	0·00	0·00	0·00	0·00	0·00	0·00	0·00	0·00	0·00	0·00	0·00	0·00	0·01	0·01	0·01
	Injuries	0·01	0·01	0·01	0·01	0·03	0·03	0·02	0·02	0·02	0·02	0·03	0·03	0·03	0·03	0·03	0·03
	Nonavoidable deaths	0·00	0·00	0·00	0·00	0·00	0·00	0·00	0·00	0·00	0·00	0·00	0·00	0·00	0·00	0·00	0·00
	Pregnancy, childbirth and perinatal period	0·19	0·00	0·00	0·00	0·00	0·00	0·00	0·00	0·00	0·00	0·00	0·00	0·00	0·00	0·00	0·00
Cyprus	Adverse effects of medical and surgical care	0·00	0·00	0·00	0·00	0·00	0·00	0·00	0·00	0·00	0·00	0·00	0·00	0·00	0·00	0·00	0·00
	Alcohol- related and drug related deaths	0·00	0·00	0·00	0·00	0·00	0·00	0·00	0·00	0·00	0·01	0·00	0·01	0·01	0·01	0·01	0·01
	Cancer	0·00	0·00	0·00	0·00	0·00	0·00	0·01	0·02	0·02	0·05	0·07	0·11	0·15	0·17	0·17	0·17
	Cardiovascular diseases	0·00	0·00	0·00	0·00	0·00	0·00	0·00	0·01	0·01	0·02	0·02	0·04	0·04	0·06	0·09	0·12

Country	Cause Group	0-1	1-4	5-9	10-14	15-19	20-24	25-29	30-34	35-39	40-44	45-49	50-54	55-59	60-64	65-69	70-74
	Cerebrovascular diseases	0·00	0·00	0·00	0·00	0·00	0·00	0·00	0·00	0·00	0·01	0·01	0·01	0·01	0·02	0·03	0·06
	Congenital malformations	0·02	0·00	0·00	0·00	0·00	0·00	0·00	0·00	0·00	0·00	0·00	0·00	0·00	0·00	0·00	0·00
	Diseases of the digestive system	0·00	0·00	0·00	0·00	0·00	0·00	0·00	0·00	0·00	0·00	0·00	0·00	0·00	0·00	0·00	0·01
	Diseases of the genitourinary system	0·00	0·00	0·00	0·00	0·00	0·00	0·00	0·00	0·00	0·00	0·00	0·00	0·00	0·00	0·01	0·01
	Diseases of the nervous system	0·00	0·00	0·00	0·00	0·00	0·00	0·00	0·00	0·00	0·00	0·00	0·00	0·00	0·00	0·00	0·00
	Diseases of the respiratory system	0·00	0·00	0·00	0·00	0·00	0·00	0·00	0·00	0·00	0·00	0·00	0·01	0·01	0·01	0·02	0·03
	Endocrine and metabolic diseases	0·00	0·00	0·00	0·00	0·00	0·00	0·00	0·00	0·00	0·00	0·00	0·00	0·01	0·02	0·04	0·08
	Infectious diseases	0·01	0·00	0·00	0·00	0·00	0·00	0·00	0·00	0·00	0·00	0·00	0·00	0·00	0·01	0·01	0·01
	Injuries	0·01	0·01	0·02	0·01	0·02	0·02	0·03	0·03	0·02	0·02	0·02	0·01	0·01	0·02	0·02	0·02
	Nonavoidable deaths	0·00	0·00	0·00	0·00	0·00	0·00	0·00	0·00	0·00	0·00	0·00	0·00	0·00	0·00	0·00	0·00
	Pregnancy, childbirth and perinatal period	0·11	0·00	0·00	0·00	0·00	0·00	0·00	0·00	0·00	0·00	0·00	0·00	0·00	0·00	0·00	0·00
Czech Republic	Adverse effects of medical and surgical care	0·00	0·00	0·00	0·00	0·00	0·00	0·00	0·00	0·00	0·00	0·00	0·00	0·00	0·00	0·00	0·00
	Alcohol- related and drug related deaths	0·00	0·00	0·00	0·00	0·00	0·00	0·01	0·01	0·02	0·03	0·04	0·04	0·04	0·04	0·02	0·01
	Cancer	0·00	0·00	0·00	0·00	0·00	0·00	0·01	0·02	0·03	0·06	0·09	0·13	0·18	0·24	0·26	0·25
	Cardiovascular diseases	0·00	0·00	0·00	0·00	0·00	0·00	0·00	0·00	0·01	0·02	0·03	0·05	0·08	0·13	0·21	0·32
	Cerebrovascular diseases	0·00	0·00	0·00	0·00	0·00	0·00	0·00	0·00	0·00	0·01	0·01	0·02	0·02	0·04	0·07	0·11
	Congenital malformations	0·02	0·00	0·00	0·00	0·00	0·00	0·00	0·00	0·00	0·00	0·00	0·00	0·00	0·00	0·00	0·00
	Diseases of the digestive system	0·00	0·00	0·00	0·00	0·00	0·00	0·00	0·00	0·00	0·00	0·00	0·00	0·01	0·01	0·01	0·02
	Diseases of the genitourinary system	0·00	0·00	0·00	0·00	0·00	0·00	0·00	0·00	0·00	0·00	0·00	0·00	0·00	0·01	0·01	0·01
	Diseases of the nervous system	0·00	0·00	0·00	0·00	0·00	0·00	0·00	0·00	0·00	0·00	0·00	0·00	0·00	0·00	0·00	0·00
	Diseases of the respiratory system	0·00	0·00	0·00	0·00	0·00	0·00	0·00	0·00	0·01	0·01	0·01	0·02	0·03	0·04	0·05	0·06
	Endocrine and metabolic diseases	0·00	0·00	0·00	0·00	0·00	0·00	0·00	0·00	0·00	0·00	0·00	0·01	0·01	0·02	0·03	0·04

Country	Cause Group	0-1	1-4	5-9	10-14	15-19	20-24	25-29	30-34	35-39	40-44	45-49	50-54	55-59	60-64	65-69	70-74
	Infectious diseases	0·00	0·00	0·00	0·00	0·00	0·00	0·00	0·00	0·00	0·00	0·00	0·00	0·01	0·01	0·01	0·02
	Injuries	0·01	0·01	0·01	0·01	0·04	0·03	0·02	0·02	0·02	0·03	0·03	0·03	0·03	0·02	0·02	0·02
	Nonavoidable deaths	0·00	0·00	0·00	0·00	0·00	0·00	0·00	0·00	0·00	0·00	0·00	0·00	0·00	0·00	0·00	0·00
	Pregnancy, childbirth and perinatal period	0·11	0·00	0·00	0·00	0·00	0·00	0·00	0·00	0·00	0·00	0·00	0·00	0·00	0·00	0·00	0·00
Denmark	Adverse effects of medical and surgical care	0·00	0·00	0·00	0·00	0·00	0·00	0·00	0·00	0·00	0·00	0·00	0·00	0·00	0·00	0·00	0·00
	Alcohol- related and drug related deaths	0·00	0·00	0·00	0·00	0·00	0·01	0·01	0·01	0·02	0·03	0·05	0·06	0·06	0·05	0·04	0·03
	Cancer	0·00	0·00	0·00	0·00	0·00	0·00	0·01	0·02	0·03	0·06	0·10	0·17	0·23	0·30	0·34	0·36
	Cardiovascular diseases	0·00	0·00	0·00	0·00	0·00	0·00	0·00	0·00	0·00	0·01	0·01	0·02	0·03	0·05	0·07	0·11
	Cerebrovascular diseases	0·00	0·00	0·00	0·00	0·00	0·00	0·00	0·00	0·01	0·01	0·01	0·02	0·02	0·03	0·05	0·07
	Congenital malformations	0·02	0·00	0·00	0·00	0·00	0·00	0·00	0·00	0·00	0·00	0·00	0·00	0·00	0·00	0·00	0·00
	Diseases of the digestive system	0·00	0·00	0·00	0·00	0·00	0·00	0·00	0·00	0·00	0·00	0·00	0·00	0·01	0·01	0·01	0·01
	Diseases of the genitourinary system	0·00	0·00	0·00	0·00	0·00	0·00	0·00	0·00	0·00	0·00	0·00	0·00	0·00	0·00	0·01	0·01
	Diseases of the nervous system	0·00	0·00	0·00	0·00	0·00	0·00	0·00	0·00	0·00	0·00	0·00	0·00	0·00	0·00	0·00	0·00
	Diseases of the respiratory system	0·00	0·00	0·00	0·00	0·00	0·00	0·00	0·00	0·00	0·00	0·01	0·02	0·04	0·07	0·09	0·14
	Endocrine and metabolic diseases	0·00	0·00	0·00	0·00	0·00	0·00	0·00	0·00	0·00	0·00	0·00	0·01	0·01	0·02	0·02	0·03
	Infectious diseases	0·00	0·00	0·00	0·00	0·00	0·00	0·00	0·00	0·00	0·00	0·00	0·00	0·01	0·01	0·01	0·01
	Injuries	0·00	0·01	0·01	0·01	0·02	0·02	0·02	0·01	0·01	0·02	0·02	0·02	0·01	0·02	0·01	0·02
	Nonavoidable deaths	0·00	0·00	0·00	0·00	0·00	0·00	0·00	0·00	0·00	0·00	0·00	0·00	0·00	0·00	0·00	0·00
	Pregnancy, childbirth and perinatal period	0·14	0·00	0·00	0·00	0·00	0·00	0·00	0·00	0·00	0·00	0·00	0·00	0·00	0·00	0·00	0·00
Estonia	Adverse effects of medical and surgical care	0·00	0·00	0·00	0·00	0·00	0·00	0·00	0·00	0·00	0·00	0·00	0·00	0·00	0·00	0·00	0·00
	Alcohol- related and drug related deaths	0·00	0·00	0·00	0·00	0·01	0·03	0·04	0·05	0·05	0·06	0·08	0·10	0·10	0·08	0·05	0·03
	Cancer	0·00	0·00	0·00	0·00	0·01	0·00	0·01	0·02	0·04	0·07	0·11	0·16	0·19	0·24	0·24	0·24

Country	Cause Group	0-1	1-4	5-9	10-14	15-19	20-24	25-29	30-34	35-39	40-44	45-49	50-54	55-59	60-64	65-69	70-74
	Cardiovascular diseases	0·00	0·00	0·00	0·00	0·00	0·00	0·00	0·00	0·01	0·02	0·03	0·07	0·12	0·19	0·29	0·45
	Cerebrovascular diseases	0·00	0·00	0·00	0·00	0·00	0·00	0·00	0·01	0·01	0·01	0·02	0·03	0·04	0·06	0·09	0·12
	Congenital malformations	0·03	0·01	0·00	0·00	0·00	0·00	0·00	0·00	0·00	0·00	0·00	0·00	0·00	0·00	0·00	0·00
	Diseases of the digestive system	0·00	0·00	0·00	0·00	0·00	0·00	0·00	0·00	0·00	0·00	0·00	0·01	0·01	0·01	0·01	0·01
	Diseases of the genitourinary system	0·00	0·00	0·00	0·00	0·00	0·00	0·00	0·00	0·00	0·00	0·00	0·00	0·00	0·00	0·00	0·00
	Diseases of the nervous system	0·00	0·00	0·00	0·00	0·00	0·00	0·00	0·00	0·00	0·00	0·01	0·00	0·00	0·00	0·00	0·00
	Diseases of the respiratory system	0·01	0·00	0·00	0·00	0·00	0·00	0·00	0·01	0·01	0·01	0·01	0·01	0·02	0·02	0·02	0·03
	Endocrine and metabolic diseases	0·00	0·00	0·00	0·00	0·00	0·00	0·00	0·00	0·00	0·00	0·01	0·01	0·01	0·02	0·02	0·02
	Infectious diseases	0·00	0·00	0·00	0·00	0·00	0·01	0·02	0·01	0·01	0·01	0·01	0·01	0·01	0·01	0·00	0·01
	Injuries	0·02	0·03	0·01	0·03	0·05	0·04	0·03	0·04	0·03	0·04	0·04	0·05	0·05	0·04	0·03	0·02
	Nonavoidable deaths	0·00	0·00	0·00	0·00	0·00	0·00	0·00	0·00	0·00	0·00	0·00	0·00	0·00	0·00	0·00	0·00
	Pregnancy, childbirth and perinatal period	0·11	0·00	0·00	0·00	0·00	0·00	0·00	0·00	0·00	0·00	0·00	0·00	0·00	0·00	0·00	0·00
Finland	Adverse effects of medical and surgical care	0·00	0·00	0·00	0·00	0·00	0·00	0·00	0·00	0·00	0·00	0·00	0·00	0·00	0·00	0·00	0·00
	Alcohol- related and drug related deaths	0·00	0·00	0·00	0·00	0·01	0·03	0·02	0·03	0·03	0·05	0·06	0·09	0·09	0·08	0·06	0·04
	Cancer	0·00	0·00	0·00	0·00	0·00	0·00	0·01	0·01	0·03	0·05	0·08	0·11	0·15	0·19	0·21	0·22
	Cardiovascular diseases	0·00	0·00	0·00	0·00	0·00	0·00	0·00	0·00	0·00	0·01	0·02	0·03	0·05	0·07	0·12	0·18
	Cerebrovascular diseases	0·00	0·00	0·00	0·00	0·00	0·00	0·00	0·00	0·01	0·01	0·01	0·02	0·03	0·03	0·05	0·08
	Congenital malformations	0·02	0·00	0·00	0·00	0·00	0·00	0·00	0·00	0·00	0·00	0·00	0·00	0·00	0·00	0·00	0·00
	Diseases of the digestive system	0·00	0·00	0·00	0·00	0·00	0·00	0·00	0·00	0·00	0·00	0·00	0·01	0·01	0·01	0·01	0·01
	Diseases of the genitourinary system	0·00	0·00	0·00	0·00	0·00	0·00	0·00	0·00	0·00	0·00	0·00	0·00	0·00	0·00	0·00	0·00
	Diseases of the nervous system	0·00	0·00	0·00	0·00	0·00	0·00	0·00	0·00	0·00	0·00	0·00	0·00	0·00	0·00	0·00	0·00
	Diseases of the respiratory system	0·00	0·00	0·00	0·00	0·00	0·00	0·00	0·00	0·00	0·00	0·01	0·01	0·01	0·02	0·03	0·04

Country	Cause Group	0-1	1-4	5-9	10-14	15-19	20-24	25-29	30-34	35-39	40-44	45-49	50-54	55-59	60-64	65-69	70-74
	Endocrine and metabolic diseases	0·00	0·00	0·00	0·00	0·00	0·00	0·00	0·00	0·00	0·00	0·01	0·01	0·01	0·01	0·01	0·01
	Infectious diseases	0·00	0·00	0·00	0·00	0·00	0·00	0·00	0·00	0·00	0·00	0·00	0·00	0·00	0·00	0·00	0·01
	Injuries	0·00	0·01	0·01	0·01	0·04	0·04	0·03	0·02	0·02	0·02	0·02	0·03	0·03	0·03	0·03	0·03
	Nonavoidable deaths	0·00	0·00	0·00	0·00	0·00	0·00	0·00	0·00	0·00	0·00	0·00	0·00	0·00	0·00	0·00	0·00
	Pregnancy, childbirth and perinatal period	0·08	0·00	0·00	0·00	0·00	0·00	0·00	0·00	0·00	0·00	0·00	0·00	0·00	0·00	0·00	0·00
France	Adverse effects of medical and surgical care	0·00	0·00	0·00	0·00	0·00	0·00	0·00	0·00	0·00	0·00	0·00	0·00	0·00	0·00	0·00	0·00
	Alcohol- related and drug related deaths	0·00	0·00	0·00	0·00	0·00	0·00	0·00	0·01	0·01	0·02	0·03	0·04	0·04	0·03	0·02	0·02
	Cancer	0·00	0·00	0·00	0·00	0·00	0·00	0·01	0·02	0·04	0·08	0·12	0·17	0·21	0·22	0·23	0·23
	Cardiovascular diseases	0·00	0·00	0·00	0·00	0·00	0·00	0·00	0·00	0·00	0·01	0·01	0·02	0·02	0·03	0·04	0·07
	Cerebrovascular diseases	0·00	0·00	0·00	0·00	0·00	0·00	0·00	0·00	0·00	0·01	0·01	0·01	0·02	0·02	0·03	0·05
	Congenital malformations	0·03	0·00	0·00	0·00	0·00	0·00	0·00	0·00	0·00	0·00	0·00	0·00	0·00	0·00	0·00	0·00
	Diseases of the digestive system	0·00	0·00	0·00	0·00	0·00	0·00	0·00	0·00	0·00	0·00	0·00	0·00	0·00	0·00	0·00	0·01
	Diseases of the genitourinary system	0·00	0·00	0·00	0·00	0·00	0·00	0·00	0·00	0·00	0·00	0·00	0·00	0·00	0·00	0·00	0·01
	Diseases of the nervous system	0·00	0·00	0·00	0·00	0·00	0·00	0·00	0·00	0·00	0·00	0·00	0·00	0·00	0·00	0·00	0·00
	Diseases of the respiratory system	0·00	0·00	0·00	0·00	0·00	0·00	0·00	0·00	0·00	0·00	0·01	0·01	0·01	0·02	0·02	0·03
	Endocrine and metabolic diseases	0·00	0·00	0·00	0·00	0·00	0·00	0·00	0·00	0·00	0·00	0·00	0·00	0·01	0·01	0·02	0·02
	Infectious diseases	0·01	0·00	0·00	0·00	0·00	0·00	0·00	0·00	0·00	0·00	0·01	0·01	0·01	0·01	0·01	0·01
	Injuries	0·01	0·01	0·01	0·01	0·03	0·03	0·02	0·02	0·03	0·03	0·03	0·03	0·03	0·03	0·03	0·03
	Nonavoidable deaths	0·00	0·00	0·00	0·00	0·00	0·00	0·00	0·00	0·00	0·00	0·00	0·00	0·00	0·00	0·00	0·00
	Pregnancy, childbirth and perinatal period	0·14	0·00	0·00	0·00	0·00	0·00	0·00	0·00	0·00	0·00	0·00	0·00	0·00	0·00	0·00	0·00
Germany	Adverse effects of medical and surgical care	0·00	0·00	0·00	0·00	0·00	0·00	0·00	0·00	0·00	0·00	0·00	0·00	0·00	0·00	0·00	0·00
	Alcohol- related and drug related deaths	0·00	0·00	0·00	0·00	0·00	0·00	0·01	0·01	0·01	0·02	0·03	0·04	0·04	0·04	0·03	0·02

Country	Cause Group	0-1	1-4	5-9	10-14	15-19	20-24	25-29	30-34	35-39	40-44	45-49	50-54	55-59	60-64	65-69	70-74
	Cancer	0·00	0·00	0·00	0·00	0·00	0·00	0·01	0·02	0·03	0·06	0·10	0·15	0·20	0·24	0·25	0·24
	Cardiovascular diseases	0·00	0·00	0·00	0·00	0·00	0·00	0·00	0·00	0·01	0·01	0·02	0·03	0·05	0·07	0·11	0·16
	Cerebrovascular diseases	0·00	0·00	0·00	0·00	0·00	0·00	0·00	0·00	0·00	0·01	0·01	0·02	0·02	0·02	0·04	0·06
	Congenital malformations	0·03	0·00	0·00	0·00	0·00	0·00	0·00	0·00	0·00	0·00	0·00	0·00	0·00	0·00	0·00	0·00
	Diseases of the digestive system	0·00	0·00	0·00	0·00	0·00	0·00	0·00	0·00	0·00	0·00	0·00	0·00	0·00	0·00	0·01	0·01
	Diseases of the genitourinary system	0·00	0·00	0·00	0·00	0·00	0·00	0·00	0·00	0·00	0·00	0·00	0·00	0·00	0·01	0·01	0·01
	Diseases of the nervous system	0·00	0·00	0·00	0·00	0·00	0·00	0·00	0·00	0·00	0·00	0·00	0·00	0·00	0·00	0·00	0·00
	Diseases of the respiratory system	0·00	0·00	0·00	0·00	0·00	0·00	0·00	0·00	0·00	0·00	0·01	0·01	0·03	0·04	0·05	0·07
	Endocrine and metabolic diseases	0·00	0·00	0·00	0·00	0·00	0·00	0·00	0·00	0·00	0·00	0·00	0·01	0·01	0·01	0·02	0·03
	Infectious diseases	0·00	0·00	0·00	0·00	0·00	0·00	0·00	0·00	0·00	0·00	0·00	0·01	0·01	0·01	0·01	0·02
	Injuries	0·00	0·01	0·01	0·01	0·02	0·02	0·02	0·02	0·01	0·02	0·02	0·02	0·02	0·02	0·02	0·02
	Nonavoidable deaths	0·00	0·00	0·00	0·00	0·00	0·00	0·00	0·00	0·00	0·00	0·00	0·00	0·00	0·00	0·00	0·00
	Pregnancy, childbirth and perinatal period	0·13	0·00	0·00	0·00	0·00	0·00	0·00	0·00	0·00	0·00	0·00	0·00	0·00	0·00	0·00	0·00
Hungary	Adverse effects of medical and surgical care	0·00	0·00	0·00	0·00	0·00	0·00	0·00	0·00	0·00	0·00	0·00	0·00	0·00	0·00	0·00	0·00
	Alcohol- related and drug related deaths	0·00	0·00	0·00	0·00	0·00	0·00	0·00	0·01	0·02	0·03	0·06	0·07	0·08	0·07	0·05	0·03
	Cancer	0·00	0·00	0·00	0·00	0·00	0·00	0·01	0·03	0·05	0·09	0·17	0·26	0·32	0·35	0·33	0·29
	Cardiovascular diseases	0·00	0·00	0·00	0·00	0·00	0·00	0·00	0·01	0·01	0·03	0·06	0·10	0·15	0·21	0·30	0·42
	Cerebrovascular diseases	0·00	0·00	0·00	0·00	0·00	0·00	0·00	0·00	0·01	0·02	0·03	0·04	0·05	0·07	0·10	0·14
	Congenital malformations	0·05	0·00	0·00	0·00	0·00	0·00	0·00	0·00	0·00	0·00	0·00	0·00	0·00	0·00	0·00	0·00
	Diseases of the digestive system	0·00	0·00	0·00	0·00	0·00	0·00	0·00	0·00	0·00	0·00	0·01	0·01	0·01	0·01	0·02	0·02
	Diseases of the genitourinary system	0·00	0·00	0·00	0·00	0·00	0·00	0·00	0·00	0·00	0·00	0·00	0·00	0·00	0·00	0·01	0·01
	Diseases of the nervous system	0·00	0·00	0·00	0·00	0·00	0·00	0·00	0·00	0·00	0·00	0·00	0·00	0·00	0·00	0·00	0·00

Country	Cause Group	0-1	1-4	5-9	10-14	15-19	20-24	25-29	30-34	35-39	40-44	45-49	50-54	55-59	60-64	65-69	70-74
	Diseases of the respiratory system	0·01	0·00	0·00	0·00	0·00	0·00	0·00	0·00	0·01	0·01	0·02	0·04	0·05	0·06	0·07	0·07
	Endocrine and metabolic diseases	0·00	0·00	0·00	0·00	0·00	0·00	0·00	0·00	0·00	0·00	0·01	0·01	0·02	0·03	0·04	0·05
	Infectious diseases	0·00	0·00	0·00	0·00	0·00	0·00	0·00	0·00	0·00	0·00	0·00	0·01	0·01	0·01	0·01	0·01
	Injuries	0·01	0·01	0·01	0·01	0·03	0·03	0·02	0·02	0·03	0·03	0·04	0·04	0·03	0·03	0·03	0·03
	Nonavoidable deaths	0·00	0·00	0·00	0·00	0·00	0·00	0·00	0·00	0·00	0·00	0·00	0·00	0·00	0·00	0·00	0·00
	Pregnancy, childbirth and perinatal period	0·21	0·00	0·00	0·00	0·00	0·00	0·00	0·00	0·00	0·00	0·00	0·00	0·00	0·00	0·00	0·00
Ireland	Adverse effects of medical and surgical care	0·00	0·00	0·00	0·00	0·00	0·00	0·00	0·00	0·00	0·00	0·00	0·00	0·00	0·00	0·00	0·00
	Alcohol- related and drug related deaths	0·00	0·00	0·00	0·00	0·00	0·01	0·01	0·02	0·02	0·02	0·03	0·03	0·03	0·03	0·02	0·01
	Cancer	0·00	0·00	0·00	0·00	0·00	0·00	0·01	0·02	0·04	0·07	0·11	0·15	0·21	0·26	0·29	0·33
	Cardiovascular diseases	0·00	0·00	0·00	0·00	0·00	0·00	0·00	0·01	0·01	0·01	0·02	0·03	0·04	0·07	0·10	0·15
	Cerebrovascular diseases	0·00	0·00	0·00	0·00	0·00	0·00	0·00	0·00	0·00	0·01	0·01	0·02	0·02	0·02	0·04	0·06
	Congenital malformations	0·04	0·00	0·00	0·00	0·00	0·00	0·00	0·00	0·00	0·00	0·00	0·00	0·00	0·00	0·00	0·00
	Diseases of the digestive system	0·00	0·00	0·00	0·00	0·00	0·00	0·00	0·00	0·00	0·00	0·00	0·00	0·00	0·00	0·01	0·01
	Diseases of the genitourinary system	0·00	0·00	0·00	0·00	0·00	0·00	0·00	0·00	0·00	0·00	0·00	0·00	0·00	0·00	0·01	0·01
	Diseases of the nervous system	0·00	0·00	0·00	0·00	0·00	0·00	0·00	0·00	0·00	0·00	0·00	0·00	0·00	0·00	0·00	0·00
	Diseases of the respiratory system	0·00	0·00	0·00	0·00	0·00	0·00	0·00	0·00	0·00	0·00	0·01	0·01	0·03	0·04	0·07	0·11
	Endocrine and metabolic diseases	0·00	0·00	0·00	0·00	0·00	0·00	0·00	0·00	0·00	0·00	0·00	0·00	0·01	0·01	0·01	0·02
	Infectious diseases	0·00	0·00	0·00	0·00	0·00	0·00	0·00	0·00	0·00	0·00	0·00	0·00	0·00	0·00	0·00	0·01
	Injuries	0·00	0·01	0·00	0·01	0·03	0·03	0·02	0·02	0·02	0·02	0·02	0·02	0·02	0·01	0·02	0·02
	Nonavoidable deaths	0·00	0·00	0·00	0·00	0·00	0·00	0·00	0·00	0·00	0·00	0·00	0·00	0·00	0·00	0·00	0·00
	Pregnancy, childbirth and perinatal period	0·11	0·00	0·00	0·00	0·00	0·00	0·00	0·00	0·00	0·00	0·00	0·00	0·00	0·00	0·00	0·00
Italy	Adverse effects of medical and surgical care	0·00	0·00	0·00	0·00	0·00	0·00	0·00	0·00	0·00	0·00	0·00	0·00	0·00	0·00	0·00	0·00

Country	Cause Group	0-1	1-4	5-9	10-14	15-19	20-24	25-29	30-34	35-39	40-44	45-49	50-54	55-59	60-64	65-69	70-74
	Alcohol- related and drug related deaths	0·00	0·00	0·00	0·00	0·00	0·00	0·00	0·00	0·00	0·01	0·01	0·01	0·01	0·01	0·01	0·02
	Cancer	0·00	0·00	0·00	0·00	0·00	0·00	0·01	0·02	0·04	0·06	0·10	0·14	0·18	0·21	0·24	0·24
	Cardiovascular diseases	0·00	0·00	0·00	0·00	0·00	0·00	0·00	0·00	0·00	0·01	0·01	0·02	0·03	0·05	0·08	0·12
	Cerebrovascular diseases	0·00	0·00	0·00	0·00	0·00	0·00	0·00	0·00	0·00	0·01	0·01	0·01	0·02	0·02	0·04	0·07
	Congenital malformations	0·03	0·00	0·00	0·00	0·00	0·00	0·00	0·00	0·00	0·00	0·00	0·00	0·00	0·00	0·00	0·00
	Diseases of the digestive system	0·00	0·00	0·00	0·00	0·00	0·00	0·00	0·00	0·00	0·00	0·00	0·00	0·00	0·00	0·00	0·01
	Diseases of the genitourinary system	0·00	0·00	0·00	0·00	0·00	0·00	0·00	0·00	0·00	0·00	0·00	0·00	0·00	0·00	0·01	0·01
	Diseases of the nervous system	0·00	0·00	0·00	0·00	0·00	0·00	0·00	0·00	0·00	0·00	0·00	0·00	0·00	0·00	0·00	0·00
	Diseases of the respiratory system	0·00	0·00	0·00	0·00	0·00	0·00	0·00	0·00	0·00	0·00	0·00	0·01	0·01	0·01	0·02	0·03
	Endocrine and metabolic diseases	0·00	0·00	0·00	0·00	0·00	0·00	0·00	0·00	0·00	0·00	0·00	0·01	0·01	0·02	0·03	0·04
	Infectious diseases	0·00	0·00	0·00	0·00	0·00	0·00	0·00	0·00	0·00	0·01	0·01	0·01	0·01	0·01	0·01	0·02
	Injuries	0·00	0·01	0·00	0·01	0·02	0·03	0·02	0·02	0·01	0·01	0·01	0·01	0·01	0·01	0·01	0·02
	Nonavoidable deaths	0·00	0·00	0·00	0·00	0·00	0·00	0·00	0·00	0·00	0·00	0·00	0·00	0·00	0·00	0·00	0·00
	Pregnancy, childbirth and perinatal period	0·14	0·00	0·00	0·00	0·00	0·00	0·00	0·00	0·00	0·00	0·00	0·00	0·00	0·00	0·00	0·00
Latvia	Adverse effects of medical and surgical care	0·00	0·00	0·00	0·00	0·00	0·00	0·00	0·00	0·00	0·00	0·00	0·00	0·00	0·00	0·00	0·00
	Alcohol- related and drug related deaths	0·00	0·00	0·00	0·00	0·01	0·01	0·01	0·03	0·05	0·07	0·08	0·09	0·08	0·07	0·04	0·02
	Cancer	0·00	0·00	0·00	0·00	0·00	0·01	0·01	0·03	0·06	0·09	0·13	0·16	0·21	0·23	0·23	0·22
	Cardiovascular diseases	0·00	0·00	0·00	0·00	0·00	0·00	0·00	0·00	0·01	0·03	0·05	0·09	0·16	0·24	0·33	0·44
	Cerebrovascular diseases	0·00	0·00	0·00	0·00	0·00	0·00	0·00	0·00	0·01	0·02	0·03	0·05	0·07	0·12	0·18	0·26
	Congenital malformations	0·04	0·00	0·00	0·00	0·00	0·00	0·00	0·00	0·00	0·00	0·00	0·00	0·00	0·00	0·00	0·00
	Diseases of the digestive system	0·00	0·00	0·00	0·00	0·00	0·00	0·00	0·00	0·00	0·01	0·01	0·01	0·01	0·01	0·01	0·01
	Diseases of the genitourinary system	0·00	0·00	0·00	0·00	0·00	0·00	0·00	0·00	0·00	0·00	0·00	0·00	0·00	0·01	0·01	0·01

Country	Cause Group	0-1	1-4	5-9	10-14	15-19	20-24	25-29	30-34	35-39	40-44	45-49	50-54	55-59	60-64	65-69	70-74
	Diseases of the nervous system	0·00	0·00	0·00	0·00	0·00	0·00	0·01	0·00	0·00	0·00	0·00	0·00	0·00	0·00	0·00	0·00
	Diseases of the respiratory system	0·01	0·00	0·00	0·00	0·00	0·00	0·01	0·01	0·01	0·02	0·02	0·02	0·02	0·03	0·02	0·02
	Endocrine and metabolic diseases	0·00	0·00	0·00	0·00	0·00	0·00	0·00	0·00	0·00	0·01	0·01	0·01	0·02	0·02	0·03	0·04
	Infectious diseases	0·01	0·00	0·00	0·00	0·00	0·00	0·01	0·02	0·02	0·02	0·02	0·01	0·01	0·01	0·01	0·01
	Injuries	0·02	0·03	0·02	0·02	0·05	0·04	0·04	0·05	0·05	0·06	0·06	0·06	0·06	0·05	0·04	0·03
	Nonavoidable deaths	0·00	0·00	0·00	0·00	0·00	0·00	0·00	0·00	0·00	0·00	0·00	0·00	0·00	0·00	0·00	0·00
	Pregnancy, childbirth and perinatal period	0·22	0·00	0·00	0·00	0·00	0·00	0·00	0·00	0·00	0·00	0·00	0·00	0·00	0·00	0·00	0·00
Lithuania	Adverse effects of medical and surgical care	0·00	0·00	0·00	0·00	0·00	0·00	0·00	0·00	0·00	0·00	0·00	0·00	0·00	0·00	0·00	0·00
	Alcohol- related and drug related deaths	0·00	0·00	0·00	0·00	0·00	0·01	0·02	0·04	0·05	0·06	0·08	0·08	0·09	0·08	0·04	0·02
	Cancer	0·00	0·00	0·00	0·00	0·00	0·00	0·01	0·03	0·06	0·09	0·13	0·17	0·20	0·21	0·22	0·20
	Cardiovascular diseases	0·00	0·00	0·00	0·00	0·00	0·00	0·01	0·01	0·02	0·03	0·05	0·09	0·15	0·23	0·33	0·44
	Cerebrovascular diseases	0·00	0·00	0·00	0·00	0·00	0·00	0·00	0·01	0·01	0·02	0·03	0·04	0·06	0·09	0·14	0·21
	Congenital malformations	0·05	0·01	0·00	0·00	0·00	0·00	0·00	0·00	0·00	0·00	0·00	0·00	0·00	0·00	0·00	0·00
	Diseases of the digestive system	0·00	0·00	0·00	0·00	0·00	0·00	0·00	0·00	0·01	0·01	0·01	0·01	0·01	0·01	0·02	0·02
	Diseases of the genitourinary system	0·00	0·00	0·00	0·00	0·00	0·00	0·00	0·00	0·00	0·00	0·00	0·00	0·00	0·00	0·01	0·01
	Diseases of the nervous system	0·00	0·00	0·00	0·00	0·00	0·00	0·00	0·00	0·00	0·00	0·00	0·00	0·00	0·00	0·00	0·00
	Diseases of the respiratory system	0·02	0·00	0·00	0·00	0·00	0·00	0·00	0·01	0·01	0·01	0·01	0·02	0·02	0·02	0·03	0·03
	Endocrine and metabolic diseases	0·00	0·00	0·00	0·00	0·00	0·00	0·00	0·00	0·00	0·00	0·01	0·00	0·01	0·02	0·02	0·02
	Infectious diseases	0·01	0·01	0·00	0·00	0·00	0·00	0·01	0·01	0·01	0·01	0·02	0·01	0·01	0·01	0·02	0·02
	Injuries	0·02	0·02	0·02	0·03	0·06	0·05	0·05	0·05	0·06	0·08	0·08	0·09	0·08	0·06	0·05	0·04
	Nonavoidable deaths	0·00	0·00	0·00	0·00	0·00	0·00	0·00	0·00	0·00	0·00	0·00	0·00	0·00	0·00	0·00	0·00
	Pregnancy, childbirth and perinatal period	0·14	0·00	0·00	0·00	0·00	0·00	0·00	0·00	0·00	0·00	0·00	0·00	0·00	0·00	0·00	0·00

Country	Cause Group	0-1	1-4	5-9	10-14	15-19	20-24	25-29	30-34	35-39	40-44	45-49	50-54	55-59	60-64	65-69	70-74
Luxembourg	Adverse effects of medical and surgical care	0·00	0·00	0·00	0·00	0·00	0·00	0·00	0·00	0·00	0·00	0·00	0·00	0·00	0·00	0·00	0·00
	Alcohol- related and drug related deaths	0·00	0·00	0·00	0·00	0·00	0·00	0·01	0·01	0·01	0·02	0·04	0·04	0·04	0·03	0·04	0·03
	Cancer	0·00	0·00	0·00	0·00	0·00	0·00	0·01	0·01	0·03	0·05	0·09	0·13	0·18	0·24	0·24	0·25
	Cardiovascular diseases	0·00	0·00	0·00	0·00	0·00	0·00	0·00	0·00	0·01	0·01	0·02	0·03	0·04	0·06	0·09	0·11
	Cerebrovascular diseases	0·00	0·00	0·00	0·00	0·00	0·00	0·00	0·00	0·00	0·01	0·01	0·02	0·02	0·03	0·03	0·07
	Congenital malformations	0·01	0·00	0·00	0·00	0·00	0·00	0·00	0·00	0·00	0·00	0·00	0·00	0·00	0·00	0·00	0·00
	Diseases of the digestive system	0·00	0·00	0·00	0·00	0·00	0·00	0·00	0·00	0·00	0·00	0·00	0·00	0·00	0·00	0·00	0·01
	Diseases of the genitourinary system	0·00	0·00	0·00	0·00	0·00	0·00	0·00	0·00	0·00	0·00	0·00	0·00	0·00	0·00	0·01	0·01
	Diseases of the nervous system	0·00	0·00	0·00	0·00	0·00	0·00	0·00	0·00	0·00	0·00	0·00	0·00	0·00	0·00	0·00	0·00
	Diseases of the respiratory system	0·00	0·00	0·00	0·00	0·00	0·00	0·00	0·00	0·00	0·01	0·00	0·01	0·01	0·04	0·06	0·07
	Endocrine and metabolic diseases	0·00	0·00	0·00	0·00	0·00	0·00	0·00	0·00	0·00	0·00	0·00	0·00	0·00	0·01	0·01	0·02
	Infectious diseases	0·00	0·00	0·00	0·00	0·00	0·00	0·00	0·00	0·00	0·00	0·00	0·01	0·01	0·01	0·01	0·02
	Injuries	0·00	0·01	0·00	0·00	0·03	0·03	0·02	0·02	0·02	0·02	0·03	0·02	0·03	0·03	0·02	0·03
	Nonavoidable deaths	0·00	0·00	0·00	0·00	0·00	0·00	0·00	0·00	0·00	0·00	0·00	0·00	0·00	0·00	0·00	0·00
	Pregnancy, childbirth and perinatal period	0·09	0·00	0·00	0·00	0·00	0·00	0·00	0·00	0·00	0·00	0·00	0·00	0·00	0·00	0·00	0·00
Malta	Adverse effects of medical and surgical care	0·00	0·00	0·00	0·00	0·00	0·00	0·00	0·00	0·00	0·00	0·00	0·00	0·00	0·00	0·00	0·00
	Alcohol- related and drug related deaths	0·00	0·00	0·00	0·00	0·00	0·01	0·01	0·01	0·01	0·00	0·00	0·01	0·01	0·01	0·00	0·00
	Cancer	0·00	0·00	0·00	0·00	0·00	0·00	0·01	0·02	0·03	0·05	0·09	0·14	0·18	0·22	0·23	0·22
	Cardiovascular diseases	0·00	0·00	0·00	0·00	0·00	0·00	0·00	0·00	0·00	0·01	0·03	0·03	0·05	0·10	0·14	0·23
	Cerebrovascular diseases	0·00	0·00	0·00	0·01	0·00	0·00	0·00	0·00	0·00	0·01	0·01	0·01	0·02	0·03	0·05	0·09
	Congenital malformations	0·08	0·00	0·00	0·00	0·00	0·00	0·00	0·00	0·00	0·00	0·00	0·00	0·00	0·00	0·00	0·00
	Diseases of the digestive system	0·00	0·00	0·00	0·00	0·00	0·00	0·00	0·00	0·00	0·00	0·00	0·00	0·00	0·00	0·00	0·01

Country	Cause Group	0-1	1-4	5-9	10-14	15-19	20-24	25-29	30-34	35-39	40-44	45-49	50-54	55-59	60-64	65-69	70-74
	Diseases of the genitourinary system	0·00	0·00	0·00	0·00	0·00	0·00	0·00	0·00	0·00	0·00	0·00	0·00	0·00	0·01	0·01	0·01
	Diseases of the nervous system	0·00	0·00	0·00	0·00	0·00	0·00	0·00	0·00	0·00	0·00	0·00	0·00	0·00	0·00	0·00	0·00
	Diseases of the respiratory system	0·01	0·00	0·00	0·00	0·00	0·00	0·00	0·00	0·00	0·00	0·01	0·01	0·01	0·02	0·03	0·05
	Endocrine and metabolic diseases	0·00	0·00	0·00	0·00	0·00	0·00	0·00	0·00	0·00	0·00	0·01	0·01	0·01	0·02	0·04	0·05
	Infectious diseases	0·01	0·01	0·00	0·00	0·00	0·00	0·00	0·00	0·00	0·00	0·00	0·00	0·00	0·00	0·01	0·01
	Injuries	0·01	0·01	0·00	0·02	0·02	0·02	0·01	0·01	0·02	0·01	0·01	0·01	0·01	0·01	0·02	0·02
	Nonavoidable deaths	0·00	0·00	0·00	0·00	0·00	0·00	0·00	0·00	0·00	0·00	0·00	0·00	0·00	0·00	0·00	0·00
	Pregnancy, childbirth and perinatal period	0·24	0·00	0·00	0·00	0·00	0·00	0·00	0·00	0·00	0·00	0·00	0·00	0·00	0·00	0·00	0·00
Netherlands	Adverse effects of medical and surgical care	0·00	0·00	0·00	0·00	0·00	0·00	0·00	0·00	0·00	0·00	0·00	0·00	0·00	0·00	0·00	0·00
	Alcohol- related and drug related deaths	0·00	0·00	0·00	0·00	0·00	0·00	0·01	0·01	0·01	0·01	0·01	0·02	0·02	0·02	0·01	0·01
	Cancer	0·00	0·00	0·00	0·00	0·00	0·00	0·01	0·02	0·04	0·08	0·12	0·19	0·25	0·30	0·31	0·31
	Cardiovascular diseases	0·00	0·00	0·00	0·00	0·00	0·00	0·00	0·00	0·01	0·01	0·02	0·02	0·03	0·04	0·06	0·09
	Cerebrovascular diseases	0·00	0·00	0·00	0·00	0·00	0·00	0·00	0·00	0·00	0·01	0·01	0·02	0·02	0·03	0·04	0·06
	Congenital malformations	0·03	0·00	0·00	0·00	0·00	0·00	0·00	0·00	0·00	0·00	0·00	0·00	0·00	0·00	0·00	0·00
	Diseases of the digestive system	0·00	0·00	0·00	0·00	0·00	0·00	0·00	0·00	0·00	0·00	0·00	0·00	0·00	0·00	0·00	0·01
	Diseases of the genitourinary system	0·00	0·00	0·00	0·00	0·00	0·00	0·00	0·00	0·00	0·00	0·00	0·00	0·00	0·00	0·01	0·01
	Diseases of the nervous system	0·00	0·00	0·00	0·00	0·00	0·00	0·00	0·00	0·00	0·00	0·00	0·00	0·00	0·00	0·00	0·00
	Diseases of the respiratory system	0·00	0·00	0·00	0·00	0·00	0·00	0·00	0·00	0·00	0·00	0·01	0·02	0·03	0·05	0·07	0·09
	Endocrine and metabolic diseases	0·00	0·00	0·00	0·00	0·00	0·00	0·00	0·00	0·00	0·00	0·00	0·01	0·01	0·01	0·02	0·02
	Infectious diseases	0·00	0·00	0·00	0·00	0·00	0·00	0·00	0·00	0·00	0·00	0·00	0·00	0·01	0·01	0·01	0·01
	Injuries	0·01	0·01	0·00	0·01	0·02	0·02	0·02	0·02	0·02	0·02	0·02	0·02	0·02	0·02	0·02	0·02
	Nonavoidable deaths	0·00	0·00	0·00	0·00	0·00	0·00	0·00	0·00	0·00	0·00	0·00	0·00	0·00	0·00	0·00	0·00

Country	Cause Group	0-1	1-4	5-9	10-14	15-19	20-24	25-29	30-34	35-39	40-44	45-49	50-54	55-59	60-64	65-69	70-74
	Pregnancy, childbirth and perinatal period	0·16	0·00	0·00	0·00	0·00	0·00	0·00	0·00	0·00	0·00	0·00	0·00	0·00	0·00	0·00	0·00
Poland	Adverse effects of medical and surgical care	0·00	0·00	0·00	0·00	0·00	0·00	0·00	0·00	0·00	0·00	0·00	0·00	0·00	0·00	0·00	0·00
	Alcohol- related and drug related deaths	0·00	0·00	0·00	0·00	0·00	0·00	0·01	0·01	0·02	0·03	0·03	0·04	0·04	0·03	0·02	0·01
	Cancer	0·00	0·00	0·00	0·00	0·00	0·00	0·01	0·02	0·04	0·07	0·12	0·18	0·25	0·29	0·30	0·27
	Cardiovascular diseases	0·00	0·00	0·00	0·00	0·00	0·00	0·00	0·00	0·01	0·01	0·03	0·04	0·07	0·11	0·16	0·24
	Cerebrovascular diseases	0·00	0·00	0·00	0·00	0·00	0·00	0·00	0·01	0·01	0·01	0·02	0·03	0·04	0·06	0·09	0·13
	Congenital malformations	0·06	0·01	0·00	0·00	0·00	0·00	0·00	0·00	0·00	0·00	0·00	0·00	0·00	0·00	0·00	0·00
	Diseases of the digestive system	0·00	0·00	0·00	0·00	0·00	0·00	0·00	0·00	0·00	0·00	0·01	0·01	0·01	0·01	0·01	0·02
	Diseases of the genitourinary system	0·00	0·00	0·00	0·00	0·00	0·00	0·00	0·00	0·00	0·00	0·00	0·00	0·00	0·01	0·01	0·01
	Diseases of the nervous system	0·00	0·00	0·00	0·00	0·00	0·00	0·00	0·00	0·00	0·00	0·00	0·00	0·00	0·00	0·00	0·00
	Diseases of the respiratory system	0·01	0·00	0·00	0·00	0·00	0·00	0·00	0·00	0·01	0·01	0·01	0·02	0·02	0·03	0·05	0·06
	Endocrine and metabolic diseases	0·00	0·00	0·00	0·00	0·00	0·00	0·00	0·00	0·00	0·00	0·00	0·01	0·01	0·02	0·03	0·04
	Infectious diseases	0·00	0·00	0·00	0·00	0·00	0·00	0·00	0·00	0·00	0·00	0·00	0·00	0·00	0·01	0·01	0·01
	Injuries	0·01	0·01	0·01	0·02	0·04	0·03	0·02	0·02	0·02	0·03	0·03	0·03	0·03	0·02	0·02	0·02
	Nonavoidable deaths	0·00	0·00	0·00	0·00	0·00	0·00	0·00	0·00	0·00	0·00	0·00	0·00	0·00	0·00	0·00	0·00
	Pregnancy, childbirth and perinatal period	0·19	0·00	0·00	0·00	0·00	0·00	0·00	0·00	0·00	0·00	0·00	0·00	0·00	0·00	0·00	0·00
Portugal	Adverse effects of medical and surgical care	0·00	0·00	0·00	0·00	0·00	0·00	0·00	0·00	0·00	0·00	0·00	0·00	0·00	0·00	0·00	0·00
	Alcohol- related and drug related deaths	0·00	0·00	0·00	0·00	0·00	0·00	0·00	0·00	0·01	0·01	0·02	0·02	0·01	0·01	0·01	0·01
	Cancer	0·00	0·00	0·00	0·00	0·00	0·00	0·01	0·02	0·04	0·07	0·11	0·14	0·17	0·18	0·19	0·19
	Cardiovascular diseases	0·00	0·00	0·00	0·00	0·00	0·00	0·00	0·00	0·01	0·01	0·02	0·02	0·03	0·04	0·06	0·10
	Cerebrovascular diseases	0·00	0·00	0·00	0·00	0·00	0·00	0·00	0·00	0·01	0·01	0·02	0·02	0·03	0·04	0·06	0·11
	Congenital malformations	0·03	0·00	0·00	0·00	0·00	0·00	0·00	0·00	0·00	0·00	0·00	0·00	0·00	0·00	0·00	0·00

Country	Cause Group	0-1	1-4	5-9	10-14	15-19	20-24	25-29	30-34	35-39	40-44	45-49	50-54	55-59	60-64	65-69	70-74
	Diseases of the digestive system	0·00	0·00	0·00	0·00	0·00	0·00	0·00	0·00	0·00	0·00	0·00	0·00	0·00	0·00	0·01	0·01
	Diseases of the genitourinary system	0·00	0·00	0·00	0·00	0·00	0·00	0·00	0·00	0·00	0·00	0·00	0·00	0·00	0·00	0·01	0·01
	Diseases of the nervous system	0·00	0·00	0·00	0·00	0·00	0·00	0·00	0·00	0·00	0·00	0·00	0·00	0·00	0·00	0·00	0·00
	Diseases of the respiratory system	0·00	0·00	0·00	0·00	0·00	0·00	0·00	0·00	0·00	0·01	0·01	0·01	0·01	0·02	0·03	0·05
	Endocrine and metabolic diseases	0·00	0·00	0·00	0·00	0·00	0·00	0·00	0·00	0·00	0·00	0·00	0·01	0·01	0·02	0·04	0·06
	Infectious diseases	0·00	0·00	0·00	0·00	0·00	0·00	0·01	0·01	0·01	0·01	0·01	0·01	0·01	0·01	0·01	0·02
	Injuries	0·00	0·01	0·01	0·01	0·02	0·03	0·02	0·02	0·02	0·02	0·02	0·02	0·02	0·02	0·02	0·03
	Nonavoidable deaths	0·00	0·00	0·00	0·00	0·00	0·00	0·00	0·00	0·00	0·00	0·00	0·00	0·00	0·00	0·00	0·00
	Pregnancy, childbirth and perinatal period	0·14	0·00	0·00	0·00	0·00	0·00	0·00	0·00	0·00	0·00	0·00	0·00	0·00	0·00	0·00	0·00
Romania	Adverse effects of medical and surgical care	0·00	0·00	0·00	0·00	0·00	0·00	0·00	0·00	0·00	0·00	0·00	0·00	0·00	0·00	0·00	0·00
	Alcohol- related and drug related deaths	0·00	0·00	0·00	0·00	0·00	0·00	0·00	0·01	0·01	0·03	0·05	0·07	0·08	0·08	0·07	0·06
	Cancer	0·00	0·00	0·00	0·00	0·00	0·01	0·01	0·03	0·05	0·09	0·14	0·19	0·22	0·23	0·22	0·21
	Cardiovascular diseases	0·00	0·00	0·00	0·00	0·00	0·00	0·01	0·01	0·02	0·03	0·06	0·09	0·15	0·23	0·32	0·47
	Cerebrovascular diseases	0·00	0·00	0·00	0·00	0·00	0·00	0·00	0·01	0·01	0·02	0·03	0·06	0·08	0·13	0·20	0·30
	Congenital malformations	0·09	0·01	0·00	0·00	0·00	0·00	0·00	0·00	0·00	0·00	0·00	0·00	0·00	0·00	0·00	0·00
	Diseases of the digestive system	0·00	0·00	0·00	0·00	0·00	0·00	0·00	0·00	0·00	0·00	0·00	0·01	0·01	0·01	0·01	0·01
	Diseases of the genitourinary system	0·00	0·00	0·00	0·00	0·00	0·00	0·00	0·00	0·00	0·00	0·01	0·01	0·01	0·01	0·02	0·02
	Diseases of the nervous system	0·00	0·00	0·00	0·00	0·00	0·00	0·00	0·00	0·00	0·00	0·00	0·00	0·00	0·00	0·00	0·00
	Diseases of the respiratory system	0·18	0·03	0·01	0·01	0·01	0·01	0·01	0·01	0·01	0·01	0·02	0·02	0·03	0·04	0·05	0·06
	Endocrine and metabolic diseases	0·00	0·00	0·00	0·00	0·00	0·00	0·00	0·00	0·00	0·00	0·00	0·01	0·01	0·02	0·02	0·02
	Infectious diseases	0·02	0·00	0·00	0·00	0·01	0·01	0·01	0·01	0·01	0·01	0·01	0·01	0·01	0·01	0·01	0·01
	Injuries	0·02	0·03	0·02	0·03	0·04	0·03	0·02	0·03	0·03	0·03	0·03	0·03	0·03	0·03	0·03	0·02

Country	Cause Group	0-1	1-4	5-9	10-14	15-19	20-24	25-29	30-34	35-39	40-44	45-49	50-54	55-59	60-64	65-69	70-74
	Nonavoidable deaths	0·00	0·00	0·00	0·00	0·00	0·00	0·00	0·00	0·00	0·00	0·00	0·00	0·00	0·00	0·00	0·00
	Pregnancy, childbirth and perinatal period	0·24	0·00	0·00	0·00	0·00	0·00	0·00	0·00	0·00	0·00	0·00	0·00	0·00	0·00	0·00	0·00
Slovakia	Adverse effects of medical and surgical care	0·00	0·00	0·00	0·00	0·00	0·00	0·00	0·00	0·00	0·00	0·00	0·00	0·00	0·00	0·00	0·00
	Alcohol- related and drug related deaths	0·00	0·00	0·00	0·00	0·00	0·00	0·00	0·01	0·02	0·03	0·05	0·05	0·06	0·05	0·03	0·02
	Cancer	0·00	0·00	0·00	0·00	0·00	0·00	0·01	0·02	0·04	0·07	0·11	0·16	0·20	0·24	0·25	0·23
	Cardiovascular diseases	0·00	0·00	0·00	0·00	0·00	0·00	0·00	0·00	0·01	0·02	0·04	0·07	0·11	0·18	0·29	0·44
	Cerebrovascular diseases	0·00	0·00	0·00	0·00	0·00	0·00	0·00	0·00	0·01	0·01	0·02	0·03	0·04	0·06	0·09	0·14
	Congenital malformations	0·05	0·01	0·00	0·00	0·00	0·00	0·00	0·00	0·00	0·00	0·00	0·00	0·00	0·00	0·00	0·00
	Diseases of the digestive system	0·00	0·00	0·00	0·00	0·00	0·00	0·00	0·00	0·00	0·00	0·01	0·01	0·01	0·01	0·01	0·02
	Diseases of the genitourinary system	0·00	0·00	0·00	0·00	0·00	0·00	0·00	0·00	0·00	0·00	0·00	0·00	0·01	0·01	0·02	0·02
	Diseases of the nervous system	0·00	0·00	0·00	0·00	0·00	0·00	0·00	0·00	0·00	0·00	0·00	0·00	0·00	0·00	0·00	0·00
	Diseases of the respiratory system	0·03	0·01	0·01	0·00	0·00	0·00	0·00	0·01	0·01	0·01	0·01	0·02	0·03	0·03	0·04	0·06
	Endocrine and metabolic diseases	0·00	0·00	0·00	0·00	0·00	0·00	0·00	0·00	0·00	0·00	0·00	0·01	0·01	0·02	0·02	0·03
	Infectious diseases	0·01	0·00	0·00	0·00	0·00	0·00	0·00	0·00	0·00	0·00	0·00	0·00	0·00	0·01	0·01	0·01
	Injuries	0·01	0·01	0·01	0·01	0·03	0·02	0·02	0·02	0·02	0·03	0·03	0·03	0·03	0·02	0·02	0·03
	Nonavoidable deaths	0·00	0·00	0·00	0·00	0·00	0·00	0·00	0·00	0·00	0·00	0·00	0·00	0·00	0·00	0·00	0·00
	Pregnancy, childbirth and perinatal period	0·17	0·00	0·00	0·00	0·00	0·00	0·00	0·00	0·00	0·00	0·00	0·00	0·00	0·00	0·00	0·00
Slovenia	Adverse effects of medical and surgical care	0·00	0·00	0·00	0·00	0·00	0·00	0·00	0·00	0·00	0·00	0·00	0·00	0·00	0·00	0·01	0·01
	Alcohol- related and drug related deaths	0·00	0·00	0·00	0·00	0·00	0·01	0·01	0·01	0·01	0·02	0·03	0·04	0·05	0·06	0·06	0·06
	Cancer	0·00	0·00	0·00	0·00	0·00	0·00	0·01	0·02	0·04	0·06	0·10	0·17	0·23	0·26	0·27	0·27
	Cardiovascular diseases	0·00	0·00	0·00	0·00	0·00	0·00	0·00	0·00	0·00	0·01	0·01	0·03	0·04	0·06	0·09	0·15
	Cerebrovascular diseases	0·00	0·00	0·00	0·00	0·00	0·00	0·00	0·00	0·00	0·01	0·02	0·02	0·02	0·04	0·06	0·10

Country	Cause Group	0-1	1-4	5-9	10-14	15-19	20-24	25-29	30-34	35-39	40-44	45-49	50-54	55-59	60-64	65-69	70-74
	Congenital malformations	0·02	0·00	0·00	0·00	0·00	0·00	0·00	0·00	0·00	0·00	0·00	0·00	0·00	0·00	0·00	0·00
	Diseases of the digestive system	0·00	0·00	0·00	0·00	0·00	0·00	0·00	0·00	0·00	0·00	0·00	0·00	0·00	0·01	0·01	0·01
	Diseases of the genitourinary system	0·00	0·00	0·00	0·00	0·00	0·00	0·00	0·00	0·00	0·00	0·00	0·00	0·00	0·00	0·01	0·01
	Diseases of the nervous system	0·00	0·00	0·00	0·00	0·00	0·00	0·00	0·00	0·00	0·00	0·00	0·00	0·00	0·00	0·00	0·00
	Diseases of the respiratory system	0·01	0·00	0·00	0·00	0·00	0·00	0·00	0·00	0·00	0·00	0·00	0·01	0·01	0·02	0·02	0·04
	Endocrine and metabolic diseases	0·00	0·00	0·00	0·00	0·00	0·00	0·00	0·00	0·00	0·00	0·00	0·00	0·00	0·01	0·01	0·02
	Infectious diseases	0·00	0·00	0·00	0·00	0·00	0·00	0·00	0·00	0·00	0·00	0·00	0·00	0·00	0·00	0·00	0·01
	Injuries	0·00	0·01	0·01	0·01	0·03	0·03	0·02	0·02	0·02	0·03	0·03	0·03	0·03	0·03	0·03	0·04
	Nonavoidable deaths	0·00	0·00	0·00	0·00	0·00	0·00	0·00	0·00	0·00	0·00	0·00	0·00	0·00	0·00	0·00	0·00
	Pregnancy, childbirth and perinatal period	0·11	0·00	0·00	0·00	0·00	0·00	0·00	0·00	0·00	0·00	0·00	0·00	0·00	0·00	0·00	0·00
Spain	Adverse effects of medical and surgical care	0·00	0·00	0·00	0·00	0·00	0·00	0·00	0·00	0·00	0·00	0·00	0·00	0·00	0·00	0·00	0·00
	Alcohol- related and drug related deaths	0·00	0·00	0·00	0·00	0·00	0·00	0·00	0·00	0·01	0·01	0·01	0·01	0·01	0·01	0·01	0·01
	Cancer	0·00	0·00	0·00	0·00	0·00	0·00	0·01	0·02	0·03	0·06	0·10	0·14	0·17	0·18	0·19	0·19
	Cardiovascular diseases	0·00	0·00	0·00	0·00	0·00	0·00	0·00	0·00	0·00	0·01	0·01	0·02	0·03	0·04	0·06	0·10
	Cerebrovascular diseases	0·00	0·00	0·00	0·00	0·00	0·00	0·00	0·00	0·00	0·01	0·01	0·01	0·02	0·02	0·03	0·06
	Congenital malformations	0·03	0·00	0·00	0·00	0·00	0·00	0·00	0·00	0·00	0·00	0·00	0·00	0·00	0·00	0·00	0·00
	Diseases of the digestive system	0·00	0·00	0·00	0·00	0·00	0·00	0·00	0·00	0·00	0·00	0·00	0·00	0·00	0·00	0·01	0·01
	Diseases of the genitourinary system	0·00	0·00	0·00	0·00	0·00	0·00	0·00	0·00	0·00	0·00	0·00	0·00	0·00	0·00	0·01	0·01
	Diseases of the nervous system	0·00	0·00	0·00	0·00	0·00	0·00	0·00	0·00	0·00	0·00	0·00	0·00	0·00	0·00	0·00	0·00
	Diseases of the respiratory system	0·00	0·00	0·00	0·00	0·00	0·00	0·00	0·00	0·00	0·01	0·01	0·01	0·01	0·02	0·02	0·04
	Endocrine and metabolic diseases	0·00	0·00	0·00	0·00	0·00	0·00	0·00	0·00	0·00	0·00	0·00	0·00	0·00	0·01	0·01	0·02
	Infectious diseases	0·00	0·00	0·00	0·00	0·00	0·00	0·00	0·00	0·01	0·01	0·01	0·01	0·01	0·01	0·01	0·02

Country	Cause Group	0-1	1-4	5-9	10-14	15-19	20-24	25-29	30-34	35-39	40-44	45-49	50-54	55-59	60-64	65-69	70-74
	Injuries	0·00	0·01	0·00	0·01	0·02	0·02	0·02	0·02	0·02	0·02	0·02	0·02	0·02	0·02	0·02	0·02
	Nonavoidable deaths	0·00	0·00	0·00	0·00	0·00	0·00	0·00	0·00	0·00	0·00	0·00	0·00	0·00	0·00	0·00	0·00
	Pregnancy, childbirth and perinatal period	0·13	0·00	0·00	0·00	0·00	0·00	0·00	0·00	0·00	0·00	0·00	0·00	0·00	0·00	0·00	0·00
Sweden	Adverse effects of medical and surgical care	0·00	0·00	0·00	0·00	0·00	0·00	0·00	0·00	0·00	0·00	0·00	0·00	0·00	0·00	0·00	0·00
	Alcohol- related and drug related deaths	0·00	0·00	0·00	0·00	0·00	0·02	0·02	0·02	0·02	0·02	0·03	0·03	0·03	0·03	0·03	0·02
	Cancer	0·00	0·00	0·00	0·00	0·00	0·00	0·01	0·02	0·03	0·05	0·08	0·12	0·17	0·22	0·26	0·28
	Cardiovascular diseases	0·00	0·00	0·00	0·00	0·00	0·00	0·00	0·00	0·00	0·01	0·02	0·02	0·04	0·07	0·10	0·15
	Cerebrovascular diseases	0·00	0·00	0·00	0·00	0·00	0·00	0·00	0·00	0·00	0·00	0·01	0·01	0·02	0·03	0·04	0·06
	Congenital malformations	0·01	0·00	0·00	0·00	0·00	0·00	0·00	0·00	0·00	0·00	0·00	0·00	0·00	0·00	0·00	0·00
	Diseases of the digestive system	0·00	0·00	0·00	0·00	0·00	0·00	0·00	0·00	0·00	0·00	0·00	0·00	0·00	0·01	0·01	0·01
	Diseases of the genitourinary system	0·00	0·00	0·00	0·00	0·00	0·00	0·00	0·00	0·00	0·00	0·00	0·00	0·00	0·00	0·00	0·01
	Diseases of the nervous system	0·00	0·00	0·00	0·00	0·00	0·00	0·00	0·00	0·00	0·00	0·00	0·00	0·00	0·00	0·00	0·00
	Diseases of the respiratory system	0·00	0·00	0·00	0·00	0·00	0·00	0·00	0·00	0·00	0·00	0·00	0·01	0·02	0·03	0·05	0·08
	Endocrine and metabolic diseases	0·00	0·00	0·00	0·00	0·00	0·00	0·00	0·00	0·00	0·00	0·00	0·01	0·01	0·01	0·01	0·02
	Infectious diseases	0·00	0·00	0·00	0·00	0·00	0·00	0·00	0·00	0·00	0·00	0·00	0·00	0·00	0·01	0·01	0·01
	Injuries	0·00	0·00	0·00	0·01	0·03	0·03	0·02	0·02	0·02	0·02	0·02	0·02	0·02	0·02	0·02	0·02
	Nonavoidable deaths	0·00	0·00	0·00	0·00	0·00	0·00	0·00	0·00	0·00	0·00	0·00	0·00	0·00	0·00	0·00	0·00
	Pregnancy, childbirth and perinatal period	0·08	0·00	0·00	0·00	0·00	0·00	0·00	0·00	0·00	0·00	0·00	0·00	0·00	0·00	0·00	0·00
United Kingdom	Adverse effects of medical and surgical care	0·00	0·00	0·00	0·00	0·00	0·00	0·00	0·00	0·00	0·00	0·00	0·00	0·00	0·00	0·00	0·00
	Alcohol- related and drug related deaths	0·00	0·00	0·00	0·00	0·01	0·01	0·01	0·02	0·03	0·04	0·04	0·04	0·04	0·03	0·02	0·01
	Cancer	0·00	0·00	0·00	0·00	0·00	0·00	0·01	0·02	0·04	0·07	0·10	0·15	0·20	0·25	0·29	0·31
	Cardiovascular diseases	0·00	0·00	0·00	0·00	0·00	0·00	0·00	0·01	0·01	0·02	0·03	0·04	0·06	0·08	0·12	0·17

Country	Cause Group	0-1	1-4	5-9	10-14	15-19	20-24	25-29	30-34	35-39	40-44	45-49	50-54	55-59	60-64	65-69	70-74
	Cerebrovascular diseases	0·00	0·00	0·00	0·00	0·00	0·00	0·00	0·00	0·01	0·01	0·01	0·02	0·02	0·03	0·04	0·07
	Congenital malformations	0·03	0·00	0·00	0·00	0·00	0·00	0·00	0·00	0·00	0·00	0·00	0·00	0·00	0·00	0·00	0·00
	Diseases of the digestive system	0·00	0·00	0·00	0·00	0·00	0·00	0·00	0·00	0·00	0·00	0·00	0·00	0·01	0·01	0·01	0·02
	Diseases of the genitourinary system	0·00	0·00	0·00	0·00	0·00	0·00	0·00	0·00	0·00	0·00	0·00	0·00	0·00	0·00	0·00	0·01
	Diseases of the nervous system	0·00	0·00	0·00	0·00	0·00	0·00	0·00	0·00	0·00	0·00	0·00	0·00	0·00	0·00	0·00	0·00
	Diseases of the respiratory system	0·00	0·00	0·00	0·00	0·00	0·00	0·00	0·00	0·01	0·01	0·02	0·03	0·04	0·07	0·10	0·14
	Endocrine and metabolic diseases	0·00	0·00	0·00	0·00	0·00	0·00	0·00	0·00	0·00	0·00	0·00	0·00	0·01	0·01	0·01	0·01
	Infectious diseases	0·00	0·00	0·00	0·00	0·00	0·00	0·00	0·00	0·00	0·00	0·00	0·01	0·01	0·01	0·01	0·01
	Injuries	0·00	0·01	0·00	0·01	0·02	0·02	0·02	0·02	0·02	0·02	0·02	0·02	0·01	0·01	0·01	0·01
	Nonavoidable deaths	0·00	0·00	0·00	0·00	0·00	0·00	0·00	0·00	0·00	0·00	0·00	0·00	0·00	0·00	0·00	0·00
	Pregnancy, childbirth and perinatal period	0·18	0·00	0·00	0·00	0·00	0·00	0·00	0·00	0·00	0·00	0·00	0·00	0·00	0·00	0·00	0·00
Established member states	Adverse effects of medical and surgical care	0·00	0·00	0·00	0·00	0·00	0·00	0·00	0·00	0·00	0·00	0·00	0·00	0·00	0·00	0·00	0·00
	Alcohol- related and drug related deaths	0·00	0·00	0·00	0·00	0·00	0·01	0·01	0·01	0·01	0·02	0·03	0·03	0·04	0·03	0·03	0·02
	Cancer	0·00	0·00	0·00	0·00	0·00	0·00	0·01	0·02	0·04	0·06	0·10	0·15	0·19	0·24	0·25	0·26
	Cardiovascular diseases	0·00	0·00	0·00	0·00	0·00	0·00	0·00	0·00	0·01	0·01	0·02	0·03	0·04	0·06	0·09	0·13
	Cerebrovascular diseases	0·00	0·00	0·00	0·00	0·00	0·00	0·00	0·00	0·00	0·01	0·01	0·02	0·02	0·03	0·04	0·07
	Congenital malformations	0·03	0·00	0·00	0·00	0·00	0·00	0·00	0·00	0·00	0·00	0·00	0·00	0·00	0·00	0·00	0·00
	Diseases of the digestive system	0·00	0·00	0·00	0·00	0·00	0·00	0·00	0·00	0·00	0·00	0·00	0·00	0·00	0·00	0·01	0·01
	Diseases of the genitourinary system	0·00	0·00	0·00	0·00	0·00	0·00	0·00	0·00	0·00	0·00	0·00	0·00	0·00	0·00	0·01	0·01
	Diseases of the nervous system	0·00	0·00	0·00	0·00	0·00	0·00	0·00	0·00	0·00	0·00	0·00	0·00	0·00	0·00	0·00	0·00
	Diseases of the respiratory system	0·00	0·00	0·00	0·00	0·00	0·00	0·00	0·00	0·00	0·00	0·01	0·01	0·02	0·04	0·05	0·07
	Endocrine and metabolic diseases	0·00	0·00	0·00	0·00	0·00	0·00	0·00	0·00	0·00	0·00	0·00	0·01	0·01	0·01	0·02	0·03

Country	Cause Group	0-1	1-4	5-9	10-14	15-19	20-24	25-29	30-34	35-39	40-44	45-49	50-54	55-59	60-64	65-69	70-74
	Infectious diseases	0·00	0·00	0·00	0·00	0·00	0·00	0·00	0·00	0·00	0·00	0·00	0·01	0·01	0·01	0·01	0·01
	Injuries	0·00	0·01	0·00	0·01	0·03	0·03	0·02	0·02	0·02	0·02	0·02	0·02	0·02	0·02	0·02	0·02
	Nonavoidable deaths	0·00	0·00	0·00	0·00	0·00	0·00	0·00	0·00	0·00	0·00	0·00	0·00	0·00	0·00	0·00	0·00
	Pregnancy, childbirth and perinatal period	0·13	0·00	0·00	0·00	0·00	0·00	0·00	0·00	0·00	0·00	0·00	0·00	0·00	0·00	0·00	0·00
New member states	Adverse effects of medical and surgical care	0·00	0·00	0·00	0·00	0·00	0·00	0·00	0·00	0·00	0·00	0·00	0·00	0·00	0·00	0·00	0·00
	Alcohol- related and drug related deaths	0·00	0·00	0·00	0·00	0·00	0·01	0·01	0·01	0·02	0·03	0·04	0·05	0·05	0·05	0·03	0·02
	Cancer	0·00	0·00	0·00	0·00	0·00	0·00	0·01	0·02	0·04	0·07	0·11	0·17	0·21	0·24	0·24	0·23
	Cardiovascular diseases	0·00	0·00	0·00	0·00	0·00	0·00	0·00	0·00	0·01	0·02	0·04	0·06	0·10	0·16	0·23	0·33
	Cerebrovascular diseases	0·00	0·00	0·00	0·00	0·00	0·00	0·00	0·00	0·01	0·01	0·02	0·03	0·05	0·07	0·11	0·17
	Congenital malformations	0·05	0·00	0·00	0·00	0·00	0·00	0·00	0·00	0·00	0·00	0·00	0·00	0·00	0·00	0·00	0·00
	Diseases of the digestive system	0·00	0·00	0·00	0·00	0·00	0·00	0·00	0·00	0·00	0·00	0·00	0·01	0·01	0·01	0·01	0·01
	Diseases of the genitourinary system	0·00	0·00	0·00	0·00	0·00	0·00	0·00	0·00	0·00	0·00	0·00	0·00	0·00	0·01	0·01	0·01
	Diseases of the nervous system	0·00	0·00	0·00	0·00	0·00	0·00	0·00	0·00	0·00	0·00	0·00	0·00	0·00	0·00	0·00	0·00
	Diseases of the respiratory system	0·03	0·01	0·00	0·00	0·00	0·00	0·00	0·00	0·01	0·01	0·01	0·02	0·02	0·03	0·03	0·04
	Endocrine and metabolic diseases	0·00	0·00	0·00	0·00	0·00	0·00	0·00	0·00	0·00	0·00	0·00	0·01	0·01	0·02	0·03	0·04
	Infectious diseases	0·01	0·00	0·00	0·00	0·00	0·00	0·00	0·00	0·01	0·01	0·01	0·01	0·01	0·01	0·01	0·01
	Injuries	0·01	0·02	0·01	0·02	0·04	0·03	0·03	0·03	0·03	0·03	0·04	0·04	0·03	0·03	0·03	0·03
	Nonavoidable deaths	0·00	0·00	0·00	0·00	0·00	0·00	0·00	0·00	0·00	0·00	0·00	0·00	0·00	0·00	0·00	0·00
	Pregnancy, childbirth and perinatal period	0·17	0·00	0·00	0·00	0·00	0·00	0·00	0·00	0·00	0·00	0·00	0·00	0·00	0·00	0·00	0·00

Supplementary Table 7: **The average contribution of cause groups to the estimated gains in male life expectancy by new member state if they were assigned average avoidable mortality rates observed across the established member states, 2005-2019**

Country	Cause Group	0-1	1-4	5-9	10-14	15-19	20-24	25-29	30-34	35-39	40-44	45-49	50-54	55-59	60-64	65-69	70-74
Bulgaria	Adverse effects of medical and surgical care	0·00	0·00	0·00	0·00	0·00	0·00	0·00	0·00	0·00	0·00	0·00	0·00	0·00	0·00	0·00	0·00
	Alcohol- related and drug related deaths	0·00	0·00	0·00	0·00	0·00	0·00	-0·01	0·00	0·00	0·01	0·02	0·03	0·04	0·03	0·02	0·01
	Cancer	0·00	0·00	0·00	0·00	0·00	0·00	0·00	0·01	0·01	0·03	0·05	0·08	0·10	0·09	0·07	0·02
	Cardiovascular diseases	0·00	0·00	0·00	0·00	0·00	0·01	0·01	0·02	0·04	0·07	0·11	0·17	0·22	0·24	0·24	0·22
	Cerebrovascular diseases	0·01	0·00	0·00	0·00	0·00	0·00	0·00	0·01	0·01	0·03	0·05	0·09	0·13	0·17	0·22	0·24
	Congenital malformations	0·04	0·00	0·00	0·00	0·00	0·00	0·00	0·00	0·00	0·00	0·00	0·00	0·00	0·00	0·00	0·00
	Diseases of the digestive system	0·00	0·00	0·00	0·00	0·00	0·00	0·00	0·00	0·00	0·00	0·00	0·01	0·00	0·01	0·01	0·00
	Diseases of the genitourinary system	0·00	0·00	0·00	0·00	0·00	0·00	0·00	0·00	0·01	0·01	0·01	0·01	0·01	0·02	0·01	0·01
	Diseases of the nervous system	0·00	0·00	0·00	0·00	0·00	0·00	0·00	0·00	0·00	0·00	0·00	0·00	0·00	0·00	0·00	0·00
	Diseases of the respiratory system	0·07	0·02	0·01	0·01	0·01	0·01	0·01	0·01	0·01	0·01	0·02	0·02	0·02	0·02	0·01	0·00
	Endocrine and metabolic diseases	0·00	0·00	0·00	0·00	0·00	0·00	0·00	0·00	0·00	0·00	0·00	0·01	0·01	0·01	0·01	0·01
	Infectious diseases	0·02	0·00	0·00	0·00	0·00	0·00	0·00	0·01	0·00	0·00	0·00	0·00	0·01	0·00	0·00	0·00
	Injuries	0·01	0·01	0·02	0·02	0·04	0·04	0·03	0·04	0·03	0·03	0·03	0·03	0·03	0·03	0·01	0·01
	Nonavoidable deaths	0·00	0·00	0·00	0·00	0·00	0·00	0·00	0·00	0·00	0·00	0·00	0·00	0·00	0·00	0·00	0·00
	Pregnancy, childbirth and perinatal period	0·14	0·00	0·00	0·00	0·00	0·00	0·00	0·00	0·00	0·00	0·00	0·00	0·00	0·00	0·00	0·00
Croatia	Adverse effects of medical and surgical care	0·00	0·00	0·00	0·00	0·00	0·00	0·00	0·00	0·00	0·00	0·00	0·00	0·00	0·00	0·00	0·00
	Alcohol- related and drug related deaths	0·00	0·00	0·00	0·00	0·00	0·00	0·00	0·01	0·01	0·02	0·03	0·05	0·06	0·05	0·05	0·04
	Cancer	0·00	0·00	0·00	0·00	0·00	0·00	0·00	0·01	0·01	0·02	0·05	0·10	0·15	0·17	0·16	0·13
	Cardiovascular diseases	0·00	0·00	0·00	0·00	0·00	0·00	0·00	0·00	0·01	0·03	0·06	0·09	0·12	0·15	0·17	0·18
	Cerebrovascular diseases	0·00	0·00	0·00	0·00	0·00	0·00	0·00	0·00	0·00	0·01	0·02	0·04	0·06	0·09	0·12	0·15

Country	Cause Group	0-1	1-4	5-9	10-14	15-19	20-24	25-29	30-34	35-39	40-44	45-49	50-54	55-59	60-64	65-69	70-74
	Congenital malformations	0·01	0·00	0·00	0·00	0·00	0·00	0·00	0·00	0·00	0·00	0·00	0·00	0·00	0·00	0·00	0·00
	Diseases of the digestive system	0·00	0·00	0·00	0·00	0·00	0·00	0·00	0·00	0·00	0·00	0·00	0·01	0·01	0·01	0·01	0·01
	Diseases of the genitourinary system	0·00	0·00	0·00	0·00	0·00	0·00	0·00	0·00	0·00	0·00	0·00	0·00	0·00	0·00	0·00	0·00
	Diseases of the nervous system	0·00	0·00	0·00	0·00	0·00	0·00	0·00	0·00	0·00	0·00	0·00	0·00	0·00	0·00	0·00	0·00
	Diseases of the respiratory system	0·00	0·00	0·00	0·00	0·00	0·00	0·00	0·00	0·00	0·00	0·00	0·00	0·00	0·01	0·01	0·02
	Endocrine and metabolic diseases	0·00	0·00	0·00	0·00	0·00	0·00	0·00	0·00	0·00	0·00	0·00	0·01	0·01	0·02	0·02	0·03
	Infectious diseases	0·00	0·00	0·00	0·00	0·00	0·00	0·00	0·00	0·00	0·00	0·00	0·00	0·00	0·00	0·00	0·00
	Injuries	0·00	0·00	0·01	0·01	0·04	0·06	0·04	0·03	0·04	0·03	0·05	0·05	0·05	0·04	0·03	0·03
	Nonavoidable deaths	0·00	0·00	0·00	0·00	0·00	0·00	0·00	0·00	0·00	0·00	0·00	0·00	0·00	0·00	0·00	0·00
	Pregnancy, childbirth and perinatal period	0·05	0·00	0·00	0·00	0·00	0·00	0·00	0·00	0·00	0·00	0·00	0·00	0·00	0·00	0·00	0·00
Cyprus	Adverse effects of medical and surgical care	0·00	0·00	0·00	0·00	0·00	0·00	0·00	0·00	0·00	0·00	0·00	0·00	0·00	0·00	0·00	0·00
	Alcohol- related and drug related deaths	0·00	0·00	0·00	0·00	0·00	0·00	-0·01	-0·01	-0·02	-0·03	-0·04	-0·05	-0·05	-0·04	-0·02	-0·01
	Cancer	0·00	0·00	0·00	0·00	0·00	0·00	0·00	0·00	0·00	-0·01	-0·03	-0·06	-0·09	-0·09	-0·09	-0·08
	Cardiovascular diseases	0·00	0·00	0·00	0·00	0·00	0·00	0·00	0·01	0·02	0·04	0·04	0·05	0·05	0·05	0·04	0·04
	Cerebrovascular diseases	0·00	0·00	0·00	0·00	0·00	0·00	0·00	0·00	0·00	0·00	0·00	0·00	0·00	0·00	-0·01	0·00
	Congenital malformations	-0·01	0·00	0·00	0·00	0·00	0·00	0·00	0·00	0·00	0·00	0·00	0·00	0·00	0·00	0·00	0·00
	Diseases of the digestive system	0·00	0·00	0·00	0·00	0·00	0·00	0·00	0·00	0·00	0·00	0·00	0·00	0·00	0·00	0·00	0·00
	Diseases of the genitourinary system	0·00	0·00	0·00	0·00	0·00	0·00	0·00	0·00	0·00	0·00	0·00	0·00	0·01	0·01	0·01	0·01
	Diseases of the nervous system	0·00	0·00	0·00	0·00	0·00	0·00	0·00	0·00	0·00	0·00	0·00	0·00	0·00	0·00	0·00	0·00
	Diseases of the respiratory system	0·00	0·00	0·00	0·00	0·00	0·00	0·00	0·00	0·00	0·00	0·00	-0·01	-0·02	-0·03	-0·03	-0·04
	Endocrine and metabolic diseases	0·00	0·00	0·00	0·00	0·00	0·00	0·00	0·00	0·00	0·00	0·00	0·00	0·01	0·02	0·04	0·06
	Infectious diseases	0·00	0·00	0·00	0·00	0·00	0·00	0·00	0·00	0·00	0·00	-0·01	0·00	0·00	0·00	0·00	0·00

Country	Cause Group	0-1	1-4	5-9	10-14	15-19	20-24	25-29	30-34	35-39	40-44	45-49	50-54	55-59	60-64	65-69	70-74
	Injuries	0·00	0·00	0·01	0·00	0·03	0·06	0·03	0·03	0·02	0·01	0·00	-0·01	-0·01	0·00	0·00	0·00
	Nonavoidable deaths	0·04	0·01	0·01	0·00	0·00	0·00	0·01	0·01	0·01	0·02	0·05	0·09	0·11	0·10	0·08	0·05
	Pregnancy, childbirth and perinatal period	-0·01	0·00	0·00	0·00	0·00	0·00	0·00	0·00	0·00	0·00	0·00	0·00	0·00	0·00	0·00	0·00
Czech Republic	Adverse effects of medical and surgical care	0·00	0·00	0·00	0·00	0·00	0·00	0·00	0·00	0·00	0·00	0·00	0·00	0·00	0·00	0·00	0·00
	Alcohol- related and drug related deaths	0·00	0·00	0·00	0·00	0·00	-0·01	-0·01	-0·01	0·00	0·01	0·02	0·03	0·03	0·03	0·01	0·00
	Cancer	0·00	0·00	0·00	0·00	0·00	0·00	0·00	0·00	0·00	0·00	0·01	0·02	0·05	0·09	0·10	0·07
	Cardiovascular diseases	0·00	0·00	0·00	0·00	0·00	0·00	0·00	0·00	0·01	0·02	0·04	0·08	0·13	0·20	0·23	0·25
	Cerebrovascular diseases	0·00	0·00	0·00	0·00	0·00	0·00	0·00	0·00	0·00	0·00	0·01	0·01	0·02	0·04	0·05	0·06
	Congenital malformations	-0·01	0·00	0·00	0·00	0·00	0·00	0·00	0·00	0·00	0·00	0·00	0·00	0·00	0·00	0·00	0·00
	Diseases of the digestive system	0·00	0·00	0·00	0·00	0·00	0·00	0·00	0·00	0·00	0·00	0·01	0·01	0·01	0·01	0·01	0·01
	Diseases of the genitourinary system	0·00	0·00	0·00	0·00	0·00	0·00	0·00	0·00	0·00	0·00	0·00	0·00	0·00	0·00	0·00	0·00
	Diseases of the nervous system	0·00	0·00	0·00	0·00	0·00	0·00	0·00	0·00	0·00	0·00	0·00	0·00	0·00	0·00	0·00	0·00
	Diseases of the respiratory system	0·00	0·00	0·00	0·00	0·00	0·00	0·00	0·00	0·00	0·01	0·01	0·02	0·02	0·03	0·03	0·03
	Endocrine and metabolic diseases	0·00	0·00	0·00	0·00	0·00	0·00	0·00	0·00	0·00	0·00	0·00	0·00	0·01	0·01	0·02	0·02
	Infectious diseases	0·00	0·00	0·00	0·00	0·00	0·00	0·00	0·00	0·00	0·00	0·00	0·00	0·00	0·00	0·00	0·00
	Injuries	0·00	0·00	0·00	0·01	0·03	0·05	0·04	0·04	0·04	0·05	0·05	0·06	0·05	0·04	0·03	0·02
	Nonavoidable deaths	0·03	0·00	0·00	0·00	0·00	0·00	0·00	0·00	0·00	0·00	0·00	0·00	0·00	0·00	0·00	0·00
	Pregnancy, childbirth and perinatal period	-0·03	0·00	0·00	0·00	0·00	0·00	0·00	0·00	0·00	0·00	0·00	0·00	0·00	0·00	0·00	0·00
Estonia	Adverse effects of medical and surgical care	0·00	0·00	0·00	0·00	0·00	0·00	0·00	0·00	0·00	0·00	0·00	0·00	0·00	0·00	0·00	0·00
	Alcohol- related and drug related deaths	0·00	0·00	0·00	0·00	0·01	0·07	0·15	0·16	0·12	0·14	0·16	0·19	0·16	0·12	0·07	0·03
	Cancer	0·00	0·00	0·00	0·00	0·00	0·00	0·00	0·00	0·01	0·01	0·02	0·03	0·07	0·11	0·12	0·11
	Cardiovascular diseases	0·00	0·00	0·00	0·00	0·00	0·00	0·00	0·01	0·02	0·04	0·10	0·17	0·27	0·37	0·41	0·43

Country	Cause Group	0-1	1-4	5-9	10-14	15-19	20-24	25-29	30-34	35-39	40-44	45-49	50-54	55-59	60-64	65-69	70-74
	Cerebrovascular diseases	0·00	0·00	0·00	0·00	0·00	0·00	0·00	0·01	0·01	0·02	0·02	0·03	0·05	0·07	0·09	0·08
	Congenital malformations	0·01	0·00	0·00	0·00	0·00	0·00	0·00	0·00	0·00	0·00	0·00	0·00	0·00	0·00	0·00	0·00
	Diseases of the digestive system	0·00	0·00	0·00	0·00	0·00	0·00	0·00	0·01	0·01	0·01	0·01	0·02	0·02	0·01	0·01	0·01
	Diseases of the genitourinary system	0·00	0·00	0·00	0·00	0·00	0·00	0·00	0·00	0·00	0·00	0·00	0·00	0·00	0·00	0·00	0·00
	Diseases of the nervous system	0·00	0·00	0·00	0·00	0·00	0·00	0·00	0·00	0·00	0·01	0·01	0·01	0·01	0·01	0·00	0·00
	Diseases of the respiratory system	0·00	0·00	0·00	0·00	0·00	0·00	0·00	0·01	0·01	0·02	0·03	0·03	0·03	0·02	0·02	0·00
	Endocrine and metabolic diseases	0·00	0·00	0·00	0·00	0·00	0·00	0·00	0·00	0·00	0·00	0·01	0·01	0·00	0·00	0·00	0·00
	Infectious diseases	0·00	0·00	0·00	0·00	0·00	0·01	0·04	0·04	0·03	0·02	0·01	0·01	0·01	0·00	0·00	0·00
	Injuries	0·02	0·01	0·02	0·02	0·07	0·13	0·12	0·11	0·12	0·13	0·14	0·15	0·13	0·09	0·06	0·03
	Nonavoidable deaths	0·02	0·00	0·00	0·00	0·00	0·00	0·00	0·00	0·00	0·00	0·00	0·00	0·00	0·00	0·00	0·00
	Pregnancy, childbirth and perinatal period	-0·03	0·00	0·00	0·00	0·00	0·00	0·00	0·00	0·00	0·00	0·00	0·00	0·00	0·00	0·00	0·00
Hungary	Adverse effects of medical and surgical care	0·00	0·00	0·00	0·00	0·00	0·00	0·00	0·00	0·00	0·00	0·00	0·00	0·00	0·00	0·00	0·00
	Alcohol- related and drug related deaths	0·00	0·00	0·00	0·00	0·00	-0·01	0·00	0·00	0·02	0·06	0·11	0·14	0·13	0·11	0·08	0·04
	Cancer	0·00	0·00	0·00	0·00	0·00	0·00	0·00	0·01	0·01	0·04	0·11	0·21	0·29	0·28	0·22	0·14
	Cardiovascular diseases	0·00	0·00	0·00	0·00	0·00	0·00	0·00	0·01	0·02	0·06	0·12	0·20	0·27	0·32	0·34	0·34
	Cerebrovascular diseases	0·00	0·00	0·00	0·00	0·00	0·00	0·00	0·00	0·01	0·02	0·03	0·05	0·07	0·09	0·10	0·11
	Congenital malformations	0·02	0·00	0·00	0·00	0·00	0·00	0·00	0·00	0·00	0·00	0·00	0·00	0·00	0·00	0·00	0·00
	Diseases of the digestive system	0·00	0·00	0·00	0·00	0·00	0·00	0·00	0·00	0·00	0·01	0·01	0·02	0·02	0·02	0·02	0·01
	Diseases of the genitourinary system	0·00	0·00	0·00	0·00	0·00	0·00	0·00	0·00	0·00	0·00	0·00	0·00	0·00	0·00	0·00	0·00
	Diseases of the nervous system	0·00	0·00	0·00	0·00	0·00	0·00	0·00	0·00	0·00	0·00	0·00	0·00	0·00	0·00	0·00	0·00
	Diseases of the respiratory system	0·00	0·00	0·00	0·00	0·00	0·00	0·00	0·00	0·01	0·01	0·03	0·04	0·05	0·06	0·05	0·04
	Endocrine and metabolic diseases	0·00	0·00	0·00	0·00	0·00	0·00	0·00	0·00	0·00	0·00	0·01	0·01	0·02	0·02	0·02	0·02

Country	Cause Group	0-1	1-4	5-9	10-14	15-19	20-24	25-29	30-34	35-39	40-44	45-49	50-54	55-59	60-64	65-69	70-74
	Infectious diseases	0·00	0·00	0·00	0·00	0·00	0·00	0·00	0·00	0·00	0·00	0·00	0·00	0·00	0·00	0·00	0·00
	Injuries	0·00	0·00	0·00	0·01	0·01	0·02	0·03	0·04	0·05	0·07	0·09	0·09	0·08	0·06	0·04	0·03
	Nonavoidable deaths	0·00	0·00	0·00	0·00	0·00	0·00	0·00	0·00	0·00	0·00	0·00	0·00	0·00	0·00	0·00	0·00
	Pregnancy, childbirth and perinatal period	0·07	0·00	0·00	0·00	0·00	0·00	0·00	0·00	0·00	0·00	0·00	0·00	0·00	0·00	0·00	0·00
Latvia	Adverse effects of medical and surgical care	0·00	0·00	0·00	0·00	0·00	0·00	0·00	0·00	0·00	0·00	0·00	0·00	0·00	0·00	0·00	0·00
	Alcohol- related and drug related deaths	0·00	0·00	0·00	0·00	0·00	0·02	0·05	0·09	0·12	0·15	0·16	0·15	0·11	0·09	0·05	0·02
	Cancer	0·00	0·00	0·00	0·00	0·00	0·00	0·00	0·01	0·01	0·02	0·03	0·07	0·11	0·14	0·13	0·11
	Cardiovascular diseases	0·00	0·00	0·00	0·00	0·00	0·00	0·00	0·02	0·04	0·09	0·15	0·26	0·36	0·45	0·47	0·42
	Cerebrovascular diseases	0·00	0·00	0·00	0·00	0·00	0·00	0·00	0·01	0·01	0·02	0·04	0·07	0·11	0·16	0·19	0·20
	Congenital malformations	0·02	0·00	0·00	0·00	0·00	0·00	0·00	0·00	0·00	0·00	0·00	0·00	0·00	0·00	0·00	0·00
	Diseases of the digestive system	0·00	0·00	0·00	0·00	0·00	0·00	0·01	0·01	0·01	0·02	0·02	0·02	0·02	0·02	0·01	0·01
	Diseases of the genitourinary system	0·00	0·00	0·00	0·00	0·00	0·00	0·00	0·00	0·00	0·00	0·00	0·00	0·00	0·01	0·01	0·00
	Diseases of the nervous system	0·00	0·00	0·00	0·00	0·00	0·00	0·00	0·00	0·01	0·01	0·01	0·01	0·00	0·00	0·00	0·00
	Diseases of the respiratory system	0·01	0·00	0·00	0·00	0·00	0·00	0·01	0·02	0·02	0·04	0·04	0·04	0·04	0·04	0·02	0·00
	Endocrine and metabolic diseases	0·00	0·00	0·00	0·00	0·00	0·00	0·00	0·00	0·00	0·01	0·00	0·01	0·01	0·01	0·01	0·00
	Infectious diseases	0·00	0·00	0·00	0·00	0·00	0·00	0·02	0·03	0·04	0·04	0·03	0·03	0·02	0·01	0·01	0·00
	Injuries	0·01	0·02	0·03	0·02	0·09	0·13	0·17	0·17	0·19	0·21	0·21	0·19	0·16	0·11	0·07	0·03
	Nonavoidable deaths	0·01	0·00	0·00	0·00	0·00	0·00	0·00	0·00	0·00	0·00	0·00	0·00	0·00	0·00	0·00	0·00
	Pregnancy, childbirth and perinatal period	0·07	0·00	0·00	0·00	0·00	0·00	0·00	0·00	0·00	0·00	0·00	0·00	0·00	0·00	0·00	0·00
Lithuania	Adverse effects of medical and surgical care	0·00	0·00	0·00	0·00	0·00	0·00	0·00	0·00	0·00	0·00	0·00	0·00	0·00	0·00	0·00	0·00
	Alcohol- related and drug related deaths	0·00	0·00	0·00	0·00	0·00	0·02	0·05	0·09	0·13	0·14	0·14	0·13	0·09	0·07	0·03	0·01
	Cancer	0·00	0·00	0·00	0·00	0·00	0·00	0·00	0·01	0·01	0·02	0·04	0·07	0·11	0·14	0·13	0·08

Country	Cause Group	0-1	1-4	5-9	10-14	15-19	20-24	25-29	30-34	35-39	40-44	45-49	50-54	55-59	60-64	65-69	70-74
	Cardiovascular diseases	0·00	0·00	0·00	0·00	0·00	0·00	0·01	0·03	0·05	0·09	0·16	0·24	0·35	0·45	0·47	0·43
	Cerebrovascular diseases	0·00	0·00	0·00	0·00	0·00	0·00	0·00	0·01	0·02	0·02	0·04	0·05	0·08	0·11	0·14	0·13
	Congenital malformations	0·02	0·00	0·00	0·00	0·00	0·00	0·00	0·00	0·00	0·00	0·00	0·00	0·00	0·00	0·00	0·00
	Diseases of the digestive system	0·00	0·00	0·00	0·00	0·00	0·00	0·01	0·01	0·02	0·02	0·02	0·02	0·02	0·02	0·02	0·01
	Diseases of the genitourinary system	0·00	0·00	0·00	0·00	0·00	0·00	0·00	0·00	0·00	0·00	0·00	0·00	0·00	0·00	0·00	0·00
	Diseases of the nervous system	0·00	0·00	0·00	0·00	0·00	0·00	0·00	0·00	0·01	0·01	0·01	0·01	0·00	0·00	0·00	0·00
	Diseases of the respiratory system	0·01	0·00	0·00	0·00	0·00	0·00	0·01	0·02	0·02	0·03	0·04	0·04	0·05	0·05	0·04	0·02
	Endocrine and metabolic diseases	0·00	0·00	0·00	0·00	0·00	0·00	0·00	0·00	0·00	0·00	0·01	0·00	0·00	0·00	0·00	0·00
	Infectious diseases	0·00	0·00	0·00	0·00	0·00	0·00	0·01	0·03	0·03	0·04	0·03	0·03	0·03	0·02	0·02	0·01
	Injuries	0·02	0·02	0·03	0·03	0·13	0·21	0·22	0·24	0·26	0·26	0·27	0·26	0·22	0·17	0·10	0·05
	Nonavoidable deaths	0·01	0·00	0·00	0·00	0·00	0·00	0·00	0·00	0·00	0·00	0·00	0·00	0·00	0·00	0·00	0·00
	Pregnancy, childbirth and perinatal period	0·00	0·00	0·00	0·00	0·00	0·00	0·00	0·00	0·00	0·00	0·00	0·00	0·00	0·00	0·00	0·00
Malta	Adverse effects of medical and surgical care	0·00	0·00	0·00	0·00	0·00	0·00	0·00	0·00	0·00	0·00	0·00	0·00	0·00	0·00	0·00	0·00
	Alcohol- related and drug related deaths	0·00	0·00	0·00	0·00	0·00	0·00	0·00	0·00	-0·02	-0·03	-0·03	-0·05	-0·04	-0·04	-0·02	-0·01
	Cancer	0·00	0·00	0·00	0·00	0·00	0·01	0·00	0·00	0·00	-0·01	-0·03	-0·04	-0·05	-0·04	-0·04	-0·01
	Cardiovascular diseases	0·00	0·00	0·00	0·00	0·00	0·00	0·00	0·01	0·01	0·01	0·01	0·03	0·04	0·05	0·10	0·12
	Cerebrovascular diseases	0·01	0·00	0·00	0·00	0·00	0·00	0·00	0·00	0·00	0·00	-0·01	0·00	0·00	0·00	0·01	0·01
	Congenital malformations	0·04	0·00	0·00	0·00	0·00	0·00	0·00	0·00	0·00	0·00	0·00	0·00	0·00	0·00	0·00	0·00
	Diseases of the digestive system	0·00	0·00	0·00	0·00	0·00	0·00	0·00	0·00	0·00	0·00	0·00	0·00	0·00	0·00	0·00	0·00
	Diseases of the genitourinary system	0·00	0·00	0·00	0·00	0·00	0·00	0·00	0·00	0·00	0·00	0·00	0·00	0·00	0·00	0·00	0·01
	Diseases of the nervous system	0·00	0·00	0·00	0·00	0·00	0·00	0·00	0·00	0·00	0·00	0·00	0·00	0·00	0·00	0·00	0·00
	Diseases of the respiratory system	0·01	0·00	0·00	0·00	0·01	0·00	0·00	0·00	0·00	0·00	0·00	0·00	-0·02	-0·01	0·00	0·01

Country	Cause Group	0-1	1-4	5-9	10-14	15-19	20-24	25-29	30-34	35-39	40-44	45-49	50-54	55-59	60-64	65-69	70-74
	Endocrine and metabolic diseases	0·00	0·00	0·00	0·00	0·00	0·00	0·00	0·00	0·00	0·00	0·01	0·01	0·01	0·02	0·02	0·02
	Infectious diseases	0·00	0·00	0·00	0·00	0·00	0·00	0·00	0·00	0·00	-0·01	-0·01	-0·01	-0·01	-0·01	-0·01	-0·01
	Injuries	-0·01	0·00	-0·01	0·00	-0·01	-0·03	-0·01	0·00	0·00	0·01	-0·02	-0·01	-0·01	-0·01	-0·01	-0·01
	Nonavoidable deaths	0·00	0·02	0·01	0·01	0·02	0·03	0·02	0·02	0·02	0·04	0·07	0·08	0·09	0·05	0·02	0·01
	Pregnancy, childbirth and perinatal period	0·08	0·00	0·00	0·00	0·00	0·00	0·00	0·00	0·00	0·00	0·00	0·00	0·00	0·00	0·00	0·00
Poland	Adverse effects of medical and surgical care	0·00	0·00	0·00	0·00	0·00	0·00	0·00	0·00	0·00	0·00	0·00	0·00	0·00	0·00	0·00	0·00
	Alcohol- related and drug related deaths	0·00	0·00	0·00	0·00	0·00	0·00	0·00	0·02	0·03	0·05	0·06	0·06	0·05	0·04	0·02	0·00
	Cancer	0·00	0·00	0·00	0·00	0·00	0·00	0·00	0·00	0·00	0·01	0·03	0·06	0·11	0·14	0·15	0·12
	Cardiovascular diseases	0·00	0·00	0·00	0·00	0·00	0·00	0·00	0·00	0·01	0·02	0·04	0·08	0·11	0·14	0·15	0·16
	Cerebrovascular diseases	0·00	0·00	0·00	0·00	0·00	0·00	0·00	0·00	0·01	0·02	0·03	0·04	0·05	0·07	0·08	0·08
	Congenital malformations	0·03	0·01	0·00	0·00	0·00	0·00	0·00	0·00	0·00	0·00	0·00	0·00	0·00	0·00	0·00	0·00
	Diseases of the digestive system	0·00	0·00	0·00	0·00	0·00	0·00	0·00	0·01	0·01	0·01	0·01	0·01	0·01	0·01	0·01	0·01
	Diseases of the genitourinary system	0·00	0·00	0·00	0·00	0·00	0·00	0·00	0·00	0·00	0·00	0·00	0·00	0·00	0·00	0·00	0·00
	Diseases of the nervous system	0·00	0·00	0·00	0·00	0·00	0·00	0·00	0·00	0·00	0·00	0·00	0·00	0·00	0·00	0·00	0·00
	Diseases of the respiratory system	0·01	0·00	0·00	0·00	0·00	0·00	0·00	0·00	0·01	0·01	0·01	0·02	0·02	0·02	0·03	0·03
	Endocrine and metabolic diseases	0·00	0·00	0·00	0·00	0·00	0·00	0·00	0·00	0·00	0·00	0·01	0·01	0·01	0·01	0·01	0·01
	Infectious diseases	0·00	0·00	0·00	0·00	0·00	0·00	0·00	0·00	0·00	0·00	0·00	0·00	0·00	0·00	0·00	0·00
	Injuries	0·00	0·00	0·01	0·01	0·06	0·09	0·07	0·07	0·08	0·08	0·09	0·09	0·07	0·05	0·03	0·02
	Nonavoidable deaths	0·00	0·00	0·00	0·00	0·00	0·00	0·00	0·00	0·00	0·00	0·00	0·00	0·00	0·00	0·00	0·00
	Pregnancy, childbirth and perinatal period	0·06	0·00	0·00	0·00	0·00	0·00	0·00	0·00	0·00	0·00	0·00	0·00	0·00	0·00	0·00	0·00
Romania	Adverse effects of medical and surgical care	0·00	0·00	0·00	0·00	0·00	0·00	0·00	0·00	0·00	0·00	0·00	0·00	0·00	0·00	0·00	0·00
	Alcohol- related and drug related deaths	0·00	0·00	0·00	0·00	0·00	-0·01	-0·01	0·00	0·01	0·03	0·06	0·09	0·09	0·09	0·07	0·05

Country	Cause Group	0-1	1-4	5-9	10-14	15-19	20-24	25-29	30-34	35-39	40-44	45-49	50-54	55-59	60-64	65-69	70-74
	Cancer	0·00	0·00	0·00	0·00	0·00	0·00	0·00	0·01	0·01	0·04	0·08	0·13	0·17	0·15	0·11	0·06
	Cardiovascular diseases	0·00	0·00	0·00	0·00	0·00	0·01	0·01	0·02	0·03	0·07	0·12	0·18	0·24	0·29	0·32	0·35
	Cerebrovascular diseases	0·00	0·00	0·00	0·00	0·00	0·00	0·00	0·01	0·01	0·03	0·05	0·08	0·12	0·16	0·20	0·23
	Congenital malformations	0·07	0·00	0·00	0·00	0·00	0·00	0·00	0·00	0·00	0·00	0·00	0·00	0·00	0·00	0·00	0·00
	Diseases of the digestive system	0·00	0·00	0·00	0·00	0·00	0·00	0·00	0·00	0·01	0·01	0·01	0·01	0·01	0·01	0·01	0·00
	Diseases of the genitourinary system	0·00	0·00	0·00	0·00	0·00	0·00	0·00	0·00	0·00	0·00	0·00	0·01	0·01	0·01	0·01	0·01
	Diseases of the nervous system	0·00	0·00	0·00	0·00	0·00	0·00	0·00	0·00	0·00	0·00	0·00	0·00	0·00	0·00	0·00	0·00
	Diseases of the respiratory system	0·19	0·03	0·01	0·01	0·01	0·01	0·01	0·01	0·02	0·03	0·05	0·05	0·06	0·05	0·05	0·04
	Endocrine and metabolic diseases	0·00	0·00	0·00	0·00	0·00	0·00	0·00	0·00	0·00	0·00	0·00	0·00	0·00	0·00	0·00	0·00
	Infectious diseases	0·01	0·00	0·00	0·00	0·01	0·01	0·01	0·01	0·02	0·02	0·02	0·02	0·02	0·01	0·01	0·00
	Injuries	0·02	0·03	0·03	0·04	0·05	0·05	0·04	0·05	0·07	0·08	0·09	0·09	0·08	0·06	0·04	0·02
	Nonavoidable deaths	0·00	0·00	0·00	0·00	0·00	0·00	0·00	0·00	0·00	0·00	0·00	0·00	0·00	0·00	0·00	0·00
	Pregnancy, childbirth and perinatal period	0·14	0·00	0·00	0·00	0·00	0·00	0·00	0·00	0·00	0·00	0·00	0·00	0·00	0·00	0·00	0·00
Slovakia	Adverse effects of medical and surgical care	0·00	0·00	0·00	0·00	0·00	0·00	0·00	0·00	0·00	0·00	0·00	0·00	0·00	0·00	0·00	0·00
	Alcohol- related and drug related deaths	0·00	0·00	0·00	0·00	0·00	-0·01	0·00	0·00	0·02	0·04	0·06	0·08	0·07	0·08	0·05	0·02
	Cancer	0·00	0·00	0·00	0·00	0·00	0·00	0·00	0·00	0·01	0·01	0·04	0·08	0·11	0·14	0·14	0·11
	Cardiovascular diseases	0·00	0·00	0·00	0·00	0·00	0·00	0·00	0·01	0·02	0·04	0·08	0·14	0·21	0·28	0·32	0·35
	Cerebrovascular diseases	0·00	0·00	0·00	0·00	0·00	0·00	0·00	0·00	0·00	0·01	0·02	0·04	0·05	0·08	0·11	0·11
	Congenital malformations	0·03	0·00	0·00	0·00	0·00	0·00	0·00	0·00	0·00	0·00	0·00	0·00	0·00	0·00	0·00	0·00
	Diseases of the digestive system	0·00	0·00	0·00	0·00	0·00	0·00	0·00	0·00	0·01	0·01	0·01	0·01	0·01	0·02	0·01	0·01
	Diseases of the genitourinary system	0·00	0·00	0·00	0·00	0·00	0·00	0·00	0·00	0·00	0·00	0·00	0·00	0·01	0·01	0·01	0·01
	Diseases of the nervous system	0·00	0·00	0·00	0·00	0·00	0·00	0·00	0·00	0·00	0·00	0·01	0·01	0·00	0·00	0·00	0·00

Country	Cause Group	0-1	1-4	5-9	10-14	15-19	20-24	25-29	30-34	35-39	40-44	45-49	50-54	55-59	60-64	65-69	70-74
	Diseases of the respiratory system	0·03	0·01	0·00	0·00	0·00	0·00	0·00	0·00	0·01	0·01	0·02	0·03	0·03	0·04	0·03	0·04
	Endocrine and metabolic diseases	0·00	0·00	0·00	0·00	0·00	0·00	0·00	0·00	0·00	0·00	0·00	0·00	0·00	0·01	0·01	0·01
	Infectious diseases	0·01	0·00	0·00	0·00	0·00	0·00	0·00	0·00	0·00	0·00	0·00	0·00	0·00	0·00	0·00	0·00
	Injuries	0·01	0·01	0·01	0·01	0·03	0·05	0·03	0·04	0·06	0·07	0·09	0·10	0·08	0·06	0·04	0·03
	Nonavoidable deaths	0·00	0·00	0·00	0·00	0·00	0·00	0·00	0·00	0·00	0·00	0·00	0·00	0·00	0·00	0·00	0·00
	Pregnancy, childbirth and perinatal period	0·04	0·00	0·00	0·00	0·00	0·00	0·00	0·00	0·00	0·00	0·00	0·00	0·00	0·00	0·00	0·00
Slovenia	Adverse effects of medical and surgical care	0·00	0·00	0·00	0·00	0·00	0·00	0·00	0·00	0·00	0·00	0·00	0·00	0·01	0·01	0·01	0·01
	Alcohol- related and drug related deaths	0·00	0·00	0·00	0·00	0·00	0·01	0·00	0·01	0·02	0·03	0·05	0·07	0·09	0·10	0·09	0·08
	Cancer	0·00	0·00	0·00	0·00	0·00	0·00	0·00	0·00	0·00	0·01	0·02	0·04	0·08	0·08	0·09	0·08
	Cardiovascular diseases	0·00	0·00	0·00	0·00	0·00	0·00	0·00	0·00	0·00	0·00	0·01	0·02	0·02	0·04	0·03	0·04
	Cerebrovascular diseases	0·00	0·00	0·00	0·00	0·00	0·00	0·00	0·00	0·00	0·00	0·00	0·01	0·01	0·03	0·04	0·06
	Congenital malformations	-0·01	0·00	0·00	0·00	0·00	0·00	0·00	0·00	0·00	0·00	0·00	0·00	0·00	0·00	0·00	0·00
	Diseases of the digestive system	0·00	0·00	0·00	0·00	0·00	0·00	0·00	0·00	0·00	0·00	0·00	0·00	0·00	0·00	0·00	0·00
	Diseases of the genitourinary system	0·00	0·00	0·00	0·00	0·00	0·00	0·00	0·00	0·00	0·00	0·00	0·00	0·00	0·00	0·00	0·00
	Diseases of the nervous system	0·00	0·00	0·00	0·00	0·00	0·00	0·00	0·00	0·00	0·00	0·00	0·00	0·00	0·00	0·00	0·00
	Diseases of the respiratory system	0·00	0·00	0·00	0·00	0·00	0·00	0·00	0·00	0·00	0·00	0·00	-0·01	-0·01	-0·01	-0·01	-0·01
	Endocrine and metabolic diseases	0·00	0·00	0·00	0·00	0·00	0·00	0·00	0·00	0·00	0·00	0·00	0·00	0·00	0·00	0·00	0·00
	Infectious diseases	0·00	0·00	0·00	0·00	0·00	0·00	0·00	0·00	0·00	-0·01	-0·01	-0·01	-0·01	-0·01	-0·01	-0·01
	Injuries	0·00	0·00	0·00	0·00	0·03	0·05	0·04	0·03	0·03	0·05	0·05	0·06	0·05	0·05	0·04	0·04
	Nonavoidable deaths	0·06	0·01	0·00	0·00	0·00	0·00	0·00	0·00	0·00	0·00	0·00	0·00	0·00	0·00	0·00	0·00
	Pregnancy, childbirth and perinatal period	-0·04	0·00	0·00	0·00	0·00	0·00	0·00	0·00	0·00	0·00	0·00	0·00	0·00	0·00	0·00	0·00
New member states	Adverse effects of medical and surgical care	0·00	0·00	0·00	0·00	0·00	0·00	0·00	0·00	0·00	0·00	0·00	0·00	0·00	0·00	0·00	0·00

Country	Cause Group	0-1	1-4	5-9	10-14	15-19	20-24	25-29	30-34	35-39	40-44	45-49	50-54	55-59	60-64	65-69	70-74
	Alcohol- related and drug related deaths	0·00	0·00	0·00	0·00	0·00	0·01	0·02	0·03	0·03	0·05	0·06	0·07	0·06	0·06	0·04	0·02
	Cancer	0·00	0·00	0·00	0·00	0·00	0·00	0·00	0·00	0·01	0·01	0·03	0·06	0·09	0·11	0·10	0·07
	Cardiovascular diseases	0·00	0·00	0·00	0·00	0·00	0·00	0·00	0·01	0·02	0·04	0·08	0·13	0·18	0·23	0·25	0·25
	Cerebrovascular diseases	0·00	0·00	0·00	0·00	0·00	0·00	0·00	0·00	0·01	0·01	0·02	0·04	0·06	0·08	0·10	0·11
	Congenital malformations	0·02	0·00	0·00	0·00	0·00	0·00	0·00	0·00	0·00	0·00	0·00	0·00	0·00	0·00	0·00	0·00
	Diseases of the digestive system	0·00	0·00	0·00	0·00	0·00	0·00	0·00	0·00	0·01	0·01	0·01	0·01	0·01	0·01	0·01	0·01
	Diseases of the genitourinary system	0·00	0·00	0·00	0·00	0·00	0·00	0·00	0·00	0·00	0·00	0·00	0·00	0·00	0·00	0·00	0·00
	Diseases of the nervous system	0·00	0·00	0·00	0·00	0·00	0·00	0·00	0·00	0·00	0·00	0·00	0·00	0·00	0·00	0·00	0·00
	Diseases of the respiratory system	0·03	0·00	0·00	0·00	0·00	0·00	0·00	0·01	0·01	0·01	0·02	0·02	0·02	0·02	0·02	0·01
	Endocrine and metabolic diseases	0·00	0·00	0·00	0·00	0·00	0·00	0·00	0·00	0·00	0·00	0·00	0·00	0·01	0·01	0·01	0·01
	Infectious diseases	0·00	0·00	0·00	0·00	0·00	0·00	0·01	0·01	0·01	0·01	0·01	0·01	0·01	0·00	0·00	0·00
	Injuries	0·01	0·01	0·01	0·02	0·05	0·07	0·07	0·07	0·08	0·08	0·09	0·09	0·08	0·06	0·04	0·02
	Nonavoidable deaths	0·01	0·00	0·00	0·00	0·00	0·00	0·00	0·00	0·00	0·00	0·01	0·01	0·01	0·01	0·01	0·00
	Pregnancy, childbirth and perinatal period	0·04	0·00	0·00	0·00	0·00	0·00	0·00	0·00	0·00	0·00	0·00	0·00	0·00	0·00	0·00	0·00

Supplementary Table 8: **The average contribution of cause groups to the estimated gains in female life expectancy by new member state if they were assigned average avoidable mortality rates observed across the established member states, 2005-2019**

Country	Cause Group	0-1	1-4	5-9	10-14	15-19	20-24	25-29	30-34	35-39	40-44	45-49	50-54	55-59	60-64	65-69	70-74
Bulgaria	Adverse effects of medical and surgical care	0·00	0·00	0·00	0·00	0·00	0·00	0·00	0·00	0·00	0·00	0·00	0·00	0·00	0·00	0·00	0·00
	Alcohol- related and drug related deaths	0·00	0·00	0·00	0·00	0·00	0·00	0·00	0·00	0·00	0·00	0·00	-0·01	0·00	0·00	0·00	0·00
	Cancer	0·00	0·00	0·00	0·00	0·00	0·00	0·00	0·01	0·02	0·03	0·03	0·04	0·03	0·01	0·00	-0·01
	Cardiovascular diseases	0·00	0·00	0·00	0·00	0·00	0·00	0·00	0·00	0·01	0·02	0·04	0·06	0·08	0·12	0·16	0·21
	Cerebrovascular diseases	0·01	0·00	0·00	0·00	0·00	0·00	0·00	0·00	0·01	0·01	0·03	0·04	0·07	0·10	0·16	0·23
	Congenital malformations	0·03	0·00	0·00	0·00	0·00	0·00	0·00	0·00	0·00	0·00	0·00	0·00	0·00	0·00	0·00	0·00
	Diseases of the digestive system	0·00	0·00	0·00	0·00	0·00	0·00	0·00	0·00	0·00	0·00	0·00	0·00	0·00	0·00	0·00	0·00
	Diseases of the genitourinary system	0·00	0·00	0·00	0·00	0·00	0·00	0·00	0·00	0·00	0·00	0·01	0·01	0·01	0·01	0·01	0·01
	Diseases of the nervous system	0·00	0·00	0·00	0·00	0·00	0·00	0·00	0·00	0·00	0·00	0·00	0·00	0·00	0·00	0·00	0·00
	Diseases of the respiratory system	0·07	0·02	0·01	0·01	0·01	0·00	0·00	0·00	0·00	0·01	0·00	0·00	0·00	-0·01	-0·01	-0·01
	Endocrine and metabolic diseases	0·00	0·00	0·00	0·00	0·00	0·00	0·00	0·00	0·00	0·00	0·00	0·01	0·01	0·01	0·02	0·02
	Infectious diseases	0·01	0·00	0·00	0·00	0·00	0·00	0·00	0·00	0·00	0·00	0·00	0·00	0·00	0·00	0·00	0·00
	Injuries	0·01	0·01	0·01	0·01	0·02	0·01	0·00	0·01	0·01	0·01	0·01	0·01	0·00	0·00	0·00	0·00
	Nonavoidable deaths	0·00	0·00	0·00	0·00	0·00	0·00	0·00	0·00	0·00	0·00	0·00	0·00	0·00	0·00	0·00	0·00
	Pregnancy, childbirth and perinatal period	0·11	0·00	0·00	0·00	0·00	0·00	0·00	0·00	0·00	0·00	0·00	0·00	0·00	0·00	0·00	0·00
Croatia	Adverse effects of medical and surgical care	0·00	0·00	0·00	0·00	0·00	0·00	0·00	0·00	0·00	0·00	0·00	0·00	0·00	0·00	0·00	0·00
	Alcohol- related and drug related deaths	0·00	0·00	0·00	0·00	0·00	0·00	0·00	0·00	0·00	-0·01	0·00	0·00	0·00	0·01	0·01	0·01
	Cancer	0·00	0·00	0·00	0·00	0·00	0·00	0·00	0·00	0·01	0·01	0·02	0·03	0·04	0·04	0·04	0·04
	Cardiovascular diseases	0·00	0·00	0·00	0·00	0·00	0·00	0·00	0·00	0·00	0·00	0·01	0·02	0·04	0·06	0·10	0·16
	Cerebrovascular diseases	0·00	0·00	0·00	0·00	0·00	0·00	0·00	0·00	0·00	0·01	0·01	0·02	0·03	0·05	0·08	0·14
	Congenital malformations	0·01	0·00	0·00	0·00	0·00	0·00	0·00	0·00	0·00	0·00	0·00	0·00	0·00	0·00	0·00	0·00

Country	Cause Group	0-1	1-4	5-9	10-14	15-19	20-24	25-29	30-34	35-39	40-44	45-49	50-54	55-59	60-64	65-69	70-74
	Diseases of the digestive system	0·00	0·00	0·00	0·00	0·00	0·00	0·00	0·00	0·00	0·00	0·00	0·00	0·00	0·00	0·00	0·00
	Diseases of the genitourinary system	0·00	0·00	0·00	0·00	0·00	0·00	0·00	0·00	0·00	0·00	0·00	0·00	0·00	0·00	0·00	0·00
	Diseases of the nervous system	0·00	0·00	0·00	0·00	0·00	0·00	0·00	0·00	0·00	0·00	0·00	0·00	0·00	0·00	0·00	0·00
	Diseases of the respiratory system	0·00	0·00	0·00	0·00	0·00	0·00	0·00	0·00	0·00	0·00	0·00	0·00	0·00	-0·01	-0·01	-0·01
	Endocrine and metabolic diseases	0·00	0·00	0·00	0·00	0·00	0·00	0·00	0·00	0·00	0·00	0·00	0·00	0·00	0·01	0·02	0·03
	Infectious diseases	0·00	0·00	0·00	0·00	0·00	0·00	0·00	0·00	0·00	0·00	0·00	0·00	0·00	0·00	0·00	0·00
	Injuries	0·00	0·00	0·01	0·00	0·01	0·01	0·00	0·00	0·01	0·00	0·01	0·01	0·01	0·01	0·01	0·02
	Nonavoidable deaths	0·00	0·00	0·00	0·00	0·00	0·00	0·00	0·00	0·00	0·00	0·00	0·00	0·00	0·00	0·00	0·00
	Pregnancy, childbirth and perinatal period	0·05	0·00	0·00	0·00	0·00	0·00	0·00	0·00	0·00	0·00	0·00	0·00	0·00	0·00	0·00	0·00
Cyprus	Adverse effects of medical and surgical care	0·00	0·00	0·00	0·00	0·00	0·00	0·00	0·00	0·00	0·00	0·00	0·00	0·00	0·00	0·00	0·00
	Alcohol- related and drug related deaths	0·00	0·00	0·00	0·00	0·00	0·00	0·00	-0·01	-0·01	-0·01	-0·02	-0·02	-0·02	-0·02	-0·01	-0·01
	Cancer	0·00	0·00	0·00	0·00	0·00	0·00	0·00	0·00	-0·01	-0·01	-0·03	-0·04	-0·04	-0·06	-0·07	-0·07
	Cardiovascular diseases	0·00	0·00	0·00	0·00	0·00	0·00	0·00	0·00	0·00	0·01	0·00	0·01	0·00	0·00	0·01	-0·01
	Cerebrovascular diseases	0·00	0·00	0·00	0·00	0·00	0·00	0·00	0·00	0·00	0·00	0·00	0·00	0·00	0·00	-0·01	0·00
	Congenital malformations	-0·01	0·00	0·00	0·00	0·00	0·00	0·00	0·00	0·00	0·00	0·00	0·00	0·00	0·00	0·00	0·00
	Diseases of the digestive system	0·00	0·00	0·00	0·00	0·00	0·00	0·00	0·00	0·00	0·00	0·00	0·00	0·00	0·00	0·00	0·00
	Diseases of the genitourinary system	0·00	0·00	0·00	0·00	0·00	0·00	0·00	0·00	0·00	0·00	0·00	0·00	0·00	0·00	0·00	0·00
	Diseases of the nervous system	0·00	0·00	0·00	0·00	0·00	0·00	0·00	0·00	0·00	0·00	0·00	0·00	0·00	0·00	0·00	0·00
	Diseases of the respiratory system	0·00	0·00	0·00	0·00	0·00	0·00	0·00	0·00	0·00	0·00	0·00	0·00	-0·01	-0·02	-0·03	-0·03
	Endocrine and metabolic diseases	0·00	0·00	0·00	0·00	0·00	0·00	0·00	0·00	0·00	0·00	0·00	0·00	0·00	0·01	0·02	0·06
	Infectious diseases	0·00	0·00	0·00	0·00	0·00	0·00	0·00	0·00	0·00	0·00	0·00	0·00	0·00	0·00	0·00	0·00
	Injuries	0·00	0·01	0·01	0·01	0·00	0·00	0·01	0·01	0·01	0·00	0·00	-0·01	0·00	0·00	0·00	0·00

Country	Cause Group	0-1	1-4	5-9	10-14	15-19	20-24	25-29	30-34	35-39	40-44	45-49	50-54	55-59	60-64	65-69	70-74
	Nonavoidable deaths	0·05	0·01	0·00	0·01	0·01	0·02	0·01	0·01	0·02	0·02	0·07	0·07	0·08	0·10	0·10	0·07
	Pregnancy, childbirth and perinatal period	-0·03	0·00	0·00	0·00	0·00	0·00	0·00	0·00	0·00	0·00	0·00	0·00	0·00	0·00	0·00	0·00
Czech Republic	Adverse effects of medical and surgical care	0·00	0·00	0·00	0·00	0·00	0·00	0·00	0·00	0·00	0·00	0·00	0·00	0·00	0·00	0·00	0·00
	Alcohol- related and drug related deaths	0·00	0·00	0·00	0·00	0·00	0·00	0·00	0·00	0·00	0·01	0·01	0·02	0·02	0·01	0·00	0·00
	Cancer	0·00	0·00	0·00	0·00	0·00	0·00	0·00	0·00	0·00	0·00	-0·01	-0·01	0·00	0·03	0·04	0·03
	Cardiovascular diseases	0·00	0·00	0·00	0·00	0·00	0·00	0·00	0·00	0·00	0·01	0·01	0·02	0·04	0·08	0·12	0·19
	Cerebrovascular diseases	0·00	0·00	0·00	0·00	0·00	0·00	0·00	0·00	0·00	0·00	0·00	0·00	0·01	0·02	0·03	0·05
	Congenital malformations	-0·01	0·00	0·00	0·00	0·00	0·00	0·00	0·00	0·00	0·00	0·00	0·00	0·00	0·00	0·00	0·00
	Diseases of the digestive system	0·00	0·00	0·00	0·00	0·00	0·00	0·00	0·00	0·00	0·00	0·00	0·00	0·00	0·00	0·01	0·01
	Diseases of the genitourinary system	0·00	0·00	0·00	0·00	0·00	0·00	0·00	0·00	0·00	0·00	0·00	0·00	0·00	0·00	0·00	0·00
	Diseases of the nervous system	0·00	0·00	0·00	0·00	0·00	0·00	0·00	0·00	0·00	0·00	0·00	0·00	0·00	0·00	0·00	0·00
	Diseases of the respiratory system	0·00	0·00	0·00	0·00	0·00	0·00	0·00	0·00	0·00	0·00	0·00	0·00	0·00	0·01	0·01	0·01
	Endocrine and metabolic diseases	0·00	0·00	0·00	0·00	0·00	0·00	0·00	0·00	0·00	0·00	0·00	0·00	0·00	0·01	0·01	0·02
	Infectious diseases	0·00	0·00	0·00	0·00	0·00	0·00	0·00	0·00	0·00	0·00	0·00	0·00	0·00	0·00	0·00	0·00
	Injuries	0·01	0·00	0·00	0·00	0·01	0·01	0·01	0·01	0·01	0·01	0·01	0·01	0·01	0·01	0·01	0·01
	Nonavoidable deaths	0·04	0·00	0·00	0·00	0·00	0·00	0·00	0·00	0·00	0·00	0·00	0·00	0·00	0·00	0·00	0·00
	Pregnancy, childbirth and perinatal period	-0·03	0·00	0·00	0·00	0·00	0·00	0·00	0·00	0·00	0·00	0·00	0·00	0·00	0·00	0·00	0·00
Estonia	Adverse effects of medical and surgical care	0·00	0·00	0·00	0·00	0·00	0·00	0·00	0·00	0·00	0·00	0·00	0·00	0·00	0·00	0·00	0·00
	Alcohol- related and drug related deaths	0·00	0·00	0·00	0·00	0·01	0·02	0·03	0·04	0·03	0·04	0·06	0·07	0·07	0·05	0·03	0·01
	Cancer	0·00	0·00	0·00	0·00	0·00	0·00	0·00	0·00	0·01	0·01	0·01	0·01	0·01	0·03	0·01	0·02
	Cardiovascular diseases	0·00	0·00	0·00	0·00	0·00	0·00	0·00	0·00	0·00	0·01	0·02	0·04	0·08	0·13	0·20	0·31
	Cerebrovascular diseases	0·00	0·00	0·00	0·00	0·00	0·00	0·00	0·00	0·00	0·00	0·01	0·02	0·02	0·04	0·05	0·06

Country	Cause Group	0-1	1-4	5-9	10-14	15-19	20-24	25-29	30-34	35-39	40-44	45-49	50-54	55-59	60-64	65-69	70-74
	Congenital malformations	0·00	0·00	0·00	0·00	0·00	0·00	0·00	0·00	0·00	0·00	0·00	0·00	0·00	0·00	0·00	0·00
	Diseases of the digestive system	0·00	0·00	0·00	0·00	0·00	0·00	0·00	0·00	0·00	0·00	0·00	0·00	0·00	0·01	0·00	0·00
	Diseases of the genitourinary system	0·00	0·00	0·00	0·00	0·00	0·00	0·00	0·00	0·00	0·00	0·00	0·00	0·00	0·00	0·00	-0·01
	Diseases of the nervous system	0·00	0·00	0·00	0·00	0·00	0·00	0·00	0·00	0·00	0·00	0·00	0·00	0·00	0·00	0·00	0·00
	Diseases of the respiratory system	0·00	0·00	0·00	0·00	0·00	0·00	0·00	0·00	0·01	0·00	0·00	0·00	0·00	-0·01	-0·02	-0·03
	Endocrine and metabolic diseases	0·00	0·00	0·00	0·00	0·00	0·00	0·00	0·00	0·00	0·00	0·00	0·00	0·00	0·00	0·01	0·00
	Infectious diseases	0·00	0·00	0·00	0·00	0·00	0·01	0·02	0·01	0·01	0·01	0·00	0·00	0·00	0·00	-0·01	-0·01
	Injuries	0·02	0·02	0·01	0·02	0·03	0·02	0·01	0·02	0·02	0·02	0·02	0·03	0·03	0·02	0·01	0·01
	Nonavoidable deaths	0·02	0·00	0·00	0·00	0·00	0·00	0·00	0·00	0·00	0·00	0·00	0·00	0·00	0·00	0·00	0·00
	Pregnancy, childbirth and perinatal period	-0·03	0·00	0·00	0·00	0·00	0·00	0·00	0·00	0·00	0·00	0·00	0·00	0·00	0·00	0·00	0·00
Hungary	Adverse effects of medical and surgical care	0·00	0·00	0·00	0·00	0·00	0·00	0·00	0·00	0·00	0·00	0·00	0·00	0·00	0·00	0·00	0·00
	Alcohol- related and drug related deaths	0·00	0·00	0·00	0·00	0·00	0·00	0·00	0·00	0·00	0·01	0·03	0·05	0·05	0·04	0·03	0·01
	Cancer	0·00	0·00	0·00	0·00	0·00	0·00	0·00	0·01	0·02	0·03	0·07	0·12	0·15	0·15	0·12	0·09
	Cardiovascular diseases	0·00	0·00	0·00	0·00	0·00	0·00	0·00	0·00	0·01	0·02	0·04	0·07	0·11	0·16	0·22	0·30
	Cerebrovascular diseases	0·00	0·00	0·00	0·00	0·00	0·00	0·00	0·00	0·00	0·01	0·02	0·02	0·03	0·05	0·06	0·08
	Congenital malformations	0·02	0·00	0·00	0·00	0·00	0·00	0·00	0·00	0·00	0·00	0·00	0·00	0·00	0·00	0·00	0·00
	Diseases of the digestive system	0·00	0·00	0·00	0·00	0·00	0·00	0·00	0·00	0·00	0·00	0·00	0·01	0·01	0·01	0·01	0·01
	Diseases of the genitourinary system	0·00	0·00	0·00	0·00	0·00	0·00	0·00	0·00	0·00	0·00	0·00	0·00	0·00	0·00	0·00	0·00
	Diseases of the nervous system	0·00	0·00	0·00	0·00	0·00	0·00	0·00	0·00	0·00	0·00	0·00	0·00	0·00	0·00	0·00	0·00
	Diseases of the respiratory system	0·00	0·00	0·00	0·00	0·00	0·00	0·00	0·00	0·00	0·01	0·01	0·03	0·03	0·03	0·03	0·02
	Endocrine and metabolic diseases	0·00	0·00	0·00	0·00	0·00	0·00	0·00	0·00	0·00	0·00	0·00	0·01	0·01	0·02	0·02	0·03
	Infectious diseases	0·00	0·00	0·00	0·00	0·00	0·00	0·00	0·00	0·00	0·00	0·00	0·00	0·00	0·00	0·00	-0·01

Country	Cause Group	0-1	1-4	5-9	10-14	15-19	20-24	25-29	30-34	35-39	40-44	45-49	50-54	55-59	60-64	65-69	70-74
	Injuries	0·01	0·00	0·00	0·00	0·01	0·00	0·00	0·01	0·01	0·01	0·02	0·02	0·02	0·01	0·01	0·01
	Nonavoidable deaths	0·00	0·00	0·00	0·00	0·00	0·00	0·00	0·00	0·00	0·00	0·00	0·00	0·00	0·00	0·00	0·00
	Pregnancy, childbirth and perinatal period	0·07	0·00	0·00	0·00	0·00	0·00	0·00	0·00	0·00	0·00	0·00	0·00	0·00	0·00	0·00	0·00
Latvia	Adverse effects of medical and surgical care	0·00	0·00	0·00	0·00	0·00	0·00	0·00	0·00	0·00	0·00	0·00	0·00	0·00	0·00	0·00	0·00
	Alcohol- related and drug related deaths	0·00	0·00	0·00	0·00	0·00	0·00	0·01	0·02	0·04	0·05	0·06	0·06	0·05	0·04	0·02	0·01
	Cancer	0·00	0·00	0·00	0·00	0·00	0·00	0·01	0·01	0·02	0·03	0·03	0·03	0·04	0·03	0·02	0·02
	Cardiovascular diseases	0·00	0·00	0·00	0·00	0·00	0·00	0·00	0·00	0·01	0·02	0·03	0·07	0·12	0·18	0·25	0·31
	Cerebrovascular diseases	0·00	0·00	0·00	0·00	0·00	0·00	0·00	0·00	0·01	0·01	0·02	0·04	0·06	0·10	0·14	0·20
	Congenital malformations	0·01	0·00	0·00	0·00	0·00	0·00	0·00	0·00	0·00	0·00	0·00	0·00	0·00	0·00	0·00	0·00
	Diseases of the digestive system	0·00	0·00	0·00	0·00	0·00	0·00	0·00	0·00	0·00	0·00	0·01	0·00	0·01	0·01	0·01	0·01
	Diseases of the genitourinary system	0·00	0·00	0·00	0·00	0·00	0·00	0·00	0·00	0·00	0·00	0·00	0·00	0·00	0·00	0·00	0·00
	Diseases of the nervous system	0·00	0·00	0·00	0·00	0·00	0·00	0·00	0·00	0·00	0·00	0·00	0·00	0·00	0·00	0·00	0·00
	Diseases of the respiratory system	0·00	0·00	0·00	0·00	0·00	0·00	0·01	0·00	0·01	0·01	0·01	0·01	0·00	0·00	-0·02	-0·03
	Endocrine and metabolic diseases	0·00	0·00	0·00	0·00	0·00	0·00	0·00	0·00	0·00	0·00	0·00	0·01	0·01	0·01	0·02	0·02
	Infectious diseases	0·00	0·00	0·00	0·00	0·00	0·00	0·01	0·02	0·02	0·01	0·01	0·01	0·01	0·00	0·00	0·00
	Injuries	0·01	0·02	0·02	0·02	0·03	0·02	0·02	0·03	0·04	0·04	0·04	0·04	0·04	0·03	0·02	0·01
	Nonavoidable deaths	0·00	0·00	0·00	0·00	0·00	0·00	0·00	0·00	0·00	0·00	0·00	0·00	0·00	0·00	0·00	0·00
	Pregnancy, childbirth and perinatal period	0·08	0·00	0·00	0·00	0·00	0·00	0·00	0·00	0·00	0·00	0·00	0·00	0·00	0·00	0·00	0·00
Lithuania	Adverse effects of medical and surgical care	0·00	0·00	0·00	0·00	0·00	0·00	0·00	0·00	0·00	0·00	0·00	0·00	0·00	0·00	0·00	0·00
	Alcohol- related and drug related deaths	0·00	0·00	0·00	0·00	0·00	0·00	0·01	0·03	0·04	0·04	0·05	0·06	0·06	0·05	0·02	0·00
	Cancer	0·00	0·00	0·00	0·00	0·00	0·00	0·01	0·01	0·03	0·03	0·03	0·03	0·03	0·01	0·00	-0·01
	Cardiovascular diseases	0·00	0·00	0·00	0·00	0·00	0·00	0·00	0·00	0·01	0·02	0·03	0·06	0·11	0·17	0·24	0·32

Country	Cause Group	0-1	1-4	5-9	10-14	15-19	20-24	25-29	30-34	35-39	40-44	45-49	50-54	55-59	60-64	65-69	70-74
	Cerebrovascular diseases	0·00	0·00	0·00	0·00	0·00	0·00	0·00	0·00	0·01	0·01	0·02	0·03	0·04	0·07	0·10	0·15
	Congenital malformations	0·02	0·00	0·00	0·00	0·00	0·00	0·00	0·00	0·00	0·00	0·00	0·00	0·00	0·00	0·00	0·00
	Diseases of the digestive system	0·00	0·00	0·00	0·00	0·00	0·00	0·00	0·00	0·00	0·01	0·01	0·01	0·01	0·01	0·01	0·01
	Diseases of the genitourinary system	0·00	0·00	0·00	0·00	0·00	0·00	0·00	0·00	0·00	0·00	0·00	0·00	0·00	0·00	0·00	0·00
	Diseases of the nervous system	0·00	0·00	0·00	0·00	0·00	0·00	0·00	0·00	0·00	0·00	0·00	0·00	0·00	0·00	0·00	0·00
	Diseases of the respiratory system	0·01	0·00	0·00	0·00	0·00	0·00	0·00	0·01	0·01	0·01	0·01	0·00	0·00	-0·01	-0·02	-0·03
	Endocrine and metabolic diseases	0·00	0·00	0·00	0·00	0·00	0·00	0·00	0·00	0·00	0·00	0·00	0·00	0·00	0·00	0·01	0·00
	Infectious diseases	0·01	0·00	0·00	0·00	0·00	0·00	0·01	0·01	0·01	0·01	0·01	0·01	0·01	0·01	0·01	0·00
	Injuries	0·02	0·01	0·02	0·02	0·03	0·03	0·03	0·04	0·05	0·06	0·06	0·07	0·06	0·05	0·03	0·02
	Nonavoidable deaths	0·01	0·00	0·00	0·00	0·00	0·00	0·00	0·00	0·00	0·00	0·00	0·00	0·00	0·00	0·00	0·00
	Pregnancy, childbirth and perinatal period	0·00	0·00	0·00	0·00	0·00	0·00	0·00	0·00	0·00	0·00	0·00	0·00	0·00	0·00	0·00	0·00
Malta	Adverse effects of medical and surgical care	0·00	0·00	0·00	0·00	0·00	0·00	0·00	0·00	0·00	0·00	0·00	0·00	0·00	0·00	0·00	0·00
	Alcohol- related and drug related deaths	0·00	0·00	0·00	0·00	0·00	0·00	0·00	0·00	-0·01	-0·02	-0·02	-0·02	-0·02	-0·01	-0·02	-0·01
	Cancer	0·00	0·00	0·00	0·00	0·00	0·00	0·00	0·00	0·00	-0·01	-0·01	-0·02	-0·01	-0·01	-0·02	-0·02
	Cardiovascular diseases	0·00	0·00	0·00	0·00	0·00	0·00	0·00	0·00	0·00	0·00	0·01	0·01	0·02	0·04	0·05	0·09
	Cerebrovascular diseases	0·00	0·00	0·00	0·01	0·00	0·00	0·00	0·00	0·00	0·00	0·00	0·00	0·00	0·01	0·01	0·03
	Congenital malformations	0·05	0·00	0·00	0·00	0·00	0·00	0·00	0·00	0·00	0·00	0·00	0·00	0·00	0·00	0·00	0·00
	Diseases of the digestive system	0·00	0·00	0·00	0·00	0·00	0·00	0·00	0·00	0·00	0·00	0·00	0·00	0·00	0·00	0·00	0·00
	Diseases of the genitourinary system	0·00	0·00	0·00	0·00	0·00	0·00	0·00	0·00	0·00	0·00	0·00	0·00	0·00	0·00	0·01	0·00
	Diseases of the nervous system	0·00	0·00	0·00	0·00	0·00	0·00	0·00	0·00	0·00	0·00	0·00	0·00	0·00	0·00	0·00	0·00
	Diseases of the respiratory system	0·01	0·00	0·00	0·00	0·00	0·00	0·00	0·00	0·00	0·00	0·00	0·00	-0·01	-0·01	-0·02	-0·02
	Endocrine and metabolic diseases	0·00	0·00	0·00	0·00	0·00	0·00	0·00	0·00	0·00	0·00	0·01	0·00	0·01	0·01	0·02	0·02

Country	Cause Group	0-1	1-4	5-9	10-14	15-19	20-24	25-29	30-34	35-39	40-44	45-49	50-54	55-59	60-64	65-69	70-74
	Infectious diseases	0·01	0·01	0·00	0·00	0·00	0·00	0·00	0·00	0·00	0·00	0·00	0·00	-0·01	-0·01	0·00	-0·01
	Injuries	0·00	0·00	0·00	0·01	0·00	-0·01	-0·01	-0·01	0·00	-0·01	-0·01	-0·01	-0·01	-0·01	0·00	0·00
	Nonavoidable deaths	0·00	0·01	0·01	0·01	0·01	0·02	0·02	0·02	0·04	0·05	0·04	0·05	0·05	0·02	0·01	0·00
	Pregnancy, childbirth and perinatal period	0·09	0·00	0·00	0·00	0·00	0·00	0·00	0·00	0·00	0·00	0·00	0·00	0·00	0·00	0·00	0·00
Poland	Adverse effects of medical and surgical care	0·00	0·00	0·00	0·00	0·00	0·00	0·00	0·00	0·00	0·00	0·00	0·00	0·00	0·00	0·00	0·00
	Alcohol- related and drug related deaths	0·00	0·00	0·00	0·00	0·00	0·00	0·00	0·00	0·00	0·01	0·01	0·01	0·01	0·01	0·00	0·00
	Cancer	0·00	0·00	0·00	0·00	0·00	0·00	0·00	0·00	0·00	0·01	0·02	0·04	0·07	0·08	0·07	0·05
	Cardiovascular diseases	0·00	0·00	0·00	0·00	0·00	0·00	0·00	0·00	0·00	0·00	0·01	0·02	0·03	0·05	0·08	0·12
	Cerebrovascular diseases	0·00	0·00	0·00	0·00	0·00	0·00	0·00	0·00	0·00	0·01	0·01	0·02	0·02	0·04	0·05	0·07
	Congenital malformations	0·03	0·01	0·00	0·00	0·00	0·00	0·00	0·00	0·00	0·00	0·00	0·00	0·00	0·00	0·00	0·00
	Diseases of the digestive system	0·00	0·00	0·00	0·00	0·00	0·00	0·00	0·00	0·00	0·00	0·00	0·00	0·01	0·01	0·01	0·01
	Diseases of the genitourinary system	0·00	0·00	0·00	0·00	0·00	0·00	0·00	0·00	0·00	0·00	0·00	0·00	0·00	0·00	0·00	0·00
	Diseases of the nervous system	0·00	0·00	0·00	0·00	0·00	0·00	0·00	0·00	0·00	0·00	0·00	0·00	0·00	0·00	0·00	0·00
	Diseases of the respiratory system	0·01	0·00	0·00	0·00	0·00	0·00	0·00	0·00	0·00	0·00	0·00	0·00	0·00	0·00	0·00	0·00
	Endocrine and metabolic diseases	0·00	0·00	0·00	0·00	0·00	0·00	0·00	0·00	0·00	0·00	0·00	0·00	0·00	0·01	0·01	0·01
	Infectious diseases	0·00	0·00	0·00	0·00	0·00	0·00	0·00	0·00	0·00	0·00	0·00	0·00	0·00	0·00	0·00	-0·01
	Injuries	0·00	0·00	0·00	0·01	0·02	0·01	0·00	0·01	0·01	0·01	0·01	0·01	0·01	0·01	0·00	0·00
	Nonavoidable deaths	0·00	0·00	0·00	0·00	0·00	0·00	0·00	0·00	0·00	0·00	0·00	0·00	0·00	0·00	0·00	0·00
	Pregnancy, childbirth and perinatal period	0·05	0·00	0·00	0·00	0·00	0·00	0·00	0·00	0·00	0·00	0·00	0·00	0·00	0·00	0·00	0·00
Romania	Adverse effects of medical and surgical care	0·00	0·00	0·00	0·00	0·00	0·00	0·00	0·00	0·00	0·00	0·00	0·00	0·00	0·00	0·00	0·00
	Alcohol- related and drug related deaths	0·00	0·00	0·00	0·00	0·00	0·00	0·00	0·00	0·00	0·01	0·02	0·04	0·05	0·05	0·05	0·04
	Cancer	0·00	0·00	0·00	0·00	0·00	0·00	0·00	0·01	0·02	0·03	0·05	0·05	0·05	0·03	0·02	0·02

Country	Cause Group	0-1	1-4	5-9	10-14	15-19	20-24	25-29	30-34	35-39	40-44	45-49	50-54	55-59	60-64	65-69	70-74
	Cardiovascular diseases	0·00	0·00	0·00	0·00	0·00	0·00	0·00	0·01	0·01	0·02	0·04	0·07	0·11	0·17	0·24	0·35
	Cerebrovascular diseases	0·00	0·00	0·00	0·00	0·00	0·00	0·00	0·00	0·01	0·01	0·02	0·04	0·07	0·11	0·16	0·24
	Congenital malformations	0·06	0·01	0·00	0·00	0·00	0·00	0·00	0·00	0·00	0·00	0·00	0·00	0·00	0·00	0·00	0·00
	Diseases of the digestive system	0·00	0·00	0·00	0·00	0·00	0·00	0·00	0·00	0·00	0·00	0·00	0·00	0·00	0·00	0·00	0·00
	Diseases of the genitourinary system	0·00	0·00	0·00	0·00	0·00	0·00	0·00	0·00	0·00	0·00	0·00	0·01	0·01	0·01	0·01	0·01
	Diseases of the nervous system	0·00	0·00	0·00	0·00	0·00	0·00	0·00	0·00	0·00	0·00	0·00	0·00	0·00	0·00	0·00	0·00
	Diseases of the respiratory system	0·18	0·03	0·01	0·01	0·01	0·01	0·01	0·01	0·01	0·01	0·01	0·01	0·01	0·01	0·01	0·00
	Endocrine and metabolic diseases	0·00	0·00	0·00	0·00	0·00	0·00	0·00	0·00	0·00	0·00	0·00	0·00	0·00	0·01	0·01	0·00
	Infectious diseases	0·01	0·00	0·00	0·00	0·01	0·01	0·01	0·00	0·00	0·00	0·00	0·00	0·00	0·00	0·00	0·00
	Injuries	0·02	0·03	0·02	0·02	0·02	0·01	0·01	0·01	0·01	0·01	0·02	0·02	0·01	0·01	0·01	0·01
	Nonavoidable deaths	0·00	0·00	0·00	0·00	0·00	0·00	0·00	0·00	0·00	0·00	0·00	0·00	0·00	0·00	0·00	0·00
	Pregnancy, childbirth and perinatal period	0·10	0·00	0·00	0·00	0·00	0·00	0·00	0·00	0·00	0·00	0·00	0·00	0·00	0·00	0·00	0·00
Slovakia	Adverse effects of medical and surgical care	0·00	0·00	0·00	0·00	0·00	0·00	0·00	0·00	0·00	0·00	0·00	0·00	0·00	0·00	0·00	0·00
	Alcohol- related and drug related deaths	0·00	0·00	0·00	0·00	0·00	0·00	0·00	0·00	0·00	0·01	0·02	0·03	0·03	0·03	0·01	0·01
	Cancer	0·00	0·00	0·00	0·00	0·00	0·00	0·00	0·00	0·01	0·01	0·01	0·02	0·02	0·03	0·04	0·03
	Cardiovascular diseases	0·00	0·00	0·00	0·00	0·00	0·00	0·00	0·00	0·00	0·01	0·02	0·04	0·07	0·13	0·20	0·32
	Cerebrovascular diseases	0·00	0·00	0·00	0·00	0·00	0·00	0·00	0·00	0·00	0·00	0·01	0·01	0·02	0·04	0·06	0·09
	Congenital malformations	0·02	0·00	0·00	0·00	0·00	0·00	0·00	0·00	0·00	0·00	0·00	0·00	0·00	0·00	0·00	0·00
	Diseases of the digestive system	0·00	0·00	0·00	0·00	0·00	0·00	0·00	0·00	0·00	0·00	0·00	0·00	0·01	0·01	0·01	0·01
	Diseases of the genitourinary system	0·00	0·00	0·00	0·00	0·00	0·00	0·00	0·00	0·00	0·00	0·00	0·00	0·00	0·01	0·01	0·01
	Diseases of the nervous system	0·00	0·00	0·00	0·00	0·00	0·00	0·00	0·00	0·00	0·00	0·00	0·00	0·00	0·00	0·00	0·00
	Diseases of the respiratory system	0·03	0·01	0·00	0·00	0·00	0·00	0·00	0·00	0·00	0·01	0·01	0·01	0·01	0·00	0·00	0·00

Country	Cause Group	0-1	1-4	5-9	10-14	15-19	20-24	25-29	30-34	35-39	40-44	45-49	50-54	55-59	60-64	65-69	70-74
	Endocrine and metabolic diseases	0·00	0·00	0·00	0·00	0·00	0·00	0·00	0·00	0·00	0·00	0·00	0·00	0·00	0·01	0·01	0·01
	Infectious diseases	0·00	0·00	0·00	0·00	0·00	0·00	0·00	0·00	0·00	0·00	0·00	0·00	0·00	0·00	0·00	0·00
	Injuries	0·01	0·00	0·01	0·01	0·01	0·00	0·00	0·00	0·01	0·01	0·01	0·01	0·01	0·01	0·01	0·01
	Nonavoidable deaths	0·00	0·00	0·00	0·00	0·00	0·00	0·00	0·00	0·00	0·00	0·00	0·00	0·00	0·00	0·00	0·00
	Pregnancy, childbirth and perinatal period	0·03	0·00	0·00	0·00	0·00	0·00	0·00	0·00	0·00	0·00	0·00	0·00	0·00	0·00	0·00	0·00
Slovenia	Adverse effects of medical and surgical care	0·00	0·00	0·00	0·00	0·00	0·00	0·00	0·00	0·00	0·00	0·00	0·00	0·00	0·00	0·01	0·01
	Alcohol- related and drug related deaths	0·00	0·00	0·00	0·00	0·00	0·00	0·00	0·00	0·00	0·00	0·00	0·01	0·02	0·03	0·03	0·04
	Cancer	0·00	0·00	0·00	0·00	0·00	0·00	0·00	0·00	0·00	0·00	0·00	0·02	0·03	0·03	0·03	0·03
	Cardiovascular diseases	0·00	0·00	0·00	0·00	0·00	0·00	0·00	0·00	0·00	0·00	0·00	0·00	0·00	0·00	0·00	0·02
	Cerebrovascular diseases	0·00	0·00	0·00	0·00	0·00	0·00	0·00	0·00	0·00	0·00	0·00	0·00	0·00	0·01	0·02	0·03
	Congenital malformations	-0·01	0·00	0·00	0·00	0·00	0·00	0·00	0·00	0·00	0·00	0·00	0·00	0·00	0·00	0·00	0·00
	Diseases of the digestive system	0·00	0·00	0·00	0·00	0·00	0·00	0·00	0·00	0·00	0·00	0·00	0·00	0·00	0·00	0·00	0·00
	Diseases of the genitourinary system	0·00	0·00	0·00	0·00	0·00	0·00	0·00	0·00	0·00	0·00	0·00	0·00	0·00	0·00	0·00	0·00
	Diseases of the nervous system	0·00	0·00	0·00	0·00	0·00	0·00	0·00	0·00	0·00	0·00	0·00	0·00	0·00	0·00	0·00	0·00
	Diseases of the respiratory system	0·00	0·00	0·00	0·00	0·00	0·00	0·00	0·00	0·00	0·00	0·00	-0·01	-0·01	-0·02	-0·03	-0·03
	Endocrine and metabolic diseases	0·00	0·00	0·00	0·00	0·00	0·00	0·00	0·00	0·00	0·00	0·00	0·00	0·00	0·00	0·00	0·00
	Infectious diseases	0·00	0·00	0·00	0·00	0·00	0·00	0·00	0·00	0·00	0·00	0·00	0·00	0·00	-0·01	-0·01	-0·01
	Injuries	0·00	0·01	0·00	0·00	0·01	0·01	0·00	0·00	0·00	0·01	0·01	0·01	0·01	0·01	0·01	0·02
	Nonavoidable deaths	0·05	0·00	0·00	0·00	0·00	0·01	0·01	0·01	0·01	0·01	0·01	0·00	0·00	0·00	0·00	0·00
	Pregnancy, childbirth and perinatal period	-0·03	0·00	0·00	0·00	0·00	0·00	0·00	0·00	0·00	0·00	0·00	0·00	0·00	0·00	0·00	0·00
New member states	Adverse effects of medical and surgical care	0·00	0·00	0·00	0·00	0·00	0·00	0·00	0·00	0·00	0·00	0·00	0·00	0·00	0·00	0·00	0·00
	Alcohol- related and drug related deaths	0·00	0·00	0·00	0·00	0·00	0·00	0·00	0·00	0·01	0·01	0·02	0·02	0·02	0·02	0·01	0·01

Country	Cause Group	0-1	1-4	5-9	10-14	15-19	20-24	25-29	30-34	35-39	40-44	45-49	50-54	55-59	60-64	65-69	70-74
	Cancer	0·00	0·00	0·00	0·00	0·00	0·00	0·00	0·00	0·01	0·01	0·02	0·02	0·03	0·03	0·02	0·02
	Cardiovascular diseases	0·00	0·00	0·00	0·00	0·00	0·00	0·00	0·00	0·00	0·01	0·02	0·04	0·06	0·10	0·14	0·21
	Cerebrovascular diseases	0·00	0·00	0·00	0·00	0·00	0·00	0·00	0·00	0·00	0·01	0·01	0·02	0·03	0·05	0·07	0·11
	Congenital malformations	0·02	0·00	0·00	0·00	0·00	0·00	0·00	0·00	0·00	0·00	0·00	0·00	0·00	0·00	0·00	0·00
	Diseases of the digestive system	0·00	0·00	0·00	0·00	0·00	0·00	0·00	0·00	0·00	0·00	0·00	0·00	0·00	0·00	0·00	0·00
	Diseases of the genitourinary system	0·00	0·00	0·00	0·00	0·00	0·00	0·00	0·00	0·00	0·00	0·00	0·00	0·00	0·00	0·00	0·00
	Diseases of the nervous system	0·00	0·00	0·00	0·00	0·00	0·00	0·00	0·00	0·00	0·00	0·00	0·00	0·00	0·00	0·00	0·00
	Diseases of the respiratory system	0·03	0·00	0·00	0·00	0·00	0·00	0·00	0·00	0·00	0·00	0·00	0·00	0·00	0·00	-0·01	-0·01
	Endocrine and metabolic diseases	0·00	0·00	0·00	0·00	0·00	0·00	0·00	0·00	0·00	0·00	0·00	0·00	0·00	0·01	0·01	0·02
	Infectious diseases	0·00	0·00	0·00	0·00	0·00	0·00	0·00	0·00	0·00	0·00	0·00	0·00	0·00	0·00	0·00	0·00
	Injuries	0·01	0·01	0·01	0·01	0·01	0·01	0·01	0·01	0·01	0·02	0·02	0·02	0·02	0·01	0·01	0·01
	Nonavoidable deaths	0·01	0·00	0·00	0·00	0·00	0·00	0·00	0·00	0·01	0·01	0·01	0·01	0·01	0·01	0·01	0·01
	Pregnancy, childbirth and perinatal period	0·03	0·00	0·00	0·00	0·00	0·00	0·00	0·00	0·00	0·00	0·00	0·00	0·00	0·00	0·00	0·00
